# Mapping Future Energy Journeys Through Modeling the Global Transport of Synthetic Energy Carriers

**DOI:** 10.1038/s41467-026-78710-7

**Published:** 2026-11-07

**Authors:** Uwe Langenmayr, Julia Schuler, Viktor Slednev, Wolf Fichtner

**Affiliations:** Institute for Industrial Production (IIP), Karlsruhe Institute of Technology (KIT), Karlsruhe, Germany

**Keywords:** Energy economics, Energy policy, Energy supply and demand

## Abstract

Synthetic energy carriers can connect renewable-energy-rich potential production regions with distant demand centers, but the emergence of trade depends on total supply costs. Here, we develop a spatially resolved heuristic combining hydrogen production costs with ports, pipelines, and shipping routes, as well as syntheses, cracking, and reformation processes, to identify key import corridors. Across 10,883 production locations and seven different scenarios, we find that export regions connected to the importer through retrofit-ready pipelines remain competitive despite only moderate renewable potentials. Meanwhile, for remote exporters competitiveness depends on port availability and coastal access through pipeline networks. Gaseous hydrogen dominates short pipeline routes, and ammonia is preferred for maritime trade, whereas methanol and methane emerge in long multimodal chains. Additional results show that low-cost heat availability is required for the competitiveness of liquid organic hydrogen carriers, while accounting for market values of conventional counterparts boosts ammonia and methanol trade.

## Abstract (web summary)

This study maps cost-efficient global supply routes for renewable hydrogen and related fuels. It shows that infrastructure is as important as low hydrogen production costs, because pipelines, ports and coastal access enable affordable imports.

## Introduction

The Paris Agreement established a global consensus to limit the average global surface temperature increase to below 2 °C. While the increase of renewable electricity generation can be a first option to cover electricity demand, direct electrification is not possible or cost-efficient in many sectors. Especially for shipping, aviation, heavy-duty transport, and industrial applications, the need for liquid and gaseous fuels with high energy content persists^1–3^. Emissions in these sectors can be reduced using fossil fuels combined with carbon capture and storage, biogenic feedstocks, and Power-to-X (PtX) technologies. Using PtX, electricity is applied to power processes that provide the feedstock (e.g., hydrogen, carbon dioxide, nitrogen) for subsequent syntheses. If powered by renewables or nuclear, the synthesis products have a low carbon intensity^4,5^.

The cost-efficient, large-scale, and renewable production of PtX-based synthetic energy carriers (SECs) requires regions with ample land and favorable meteorological conditions for renewable electricity generation, surpassing local energy demands. As these regions are often far from major energy demand centers, electrical interconnectors are economically unviable, and pipeline or ship-based transport of SECs is required. Ammonia^2^, methane^6,7^, Fischer-Tropsch crude (FTC) or methanol (MeOH)^8–10^ are proposed as hydrogen-based SECs, with their fossil-based counterparts already traded globally on a megaton scale^11^. This established trade supports the adoption of SECs, as initial smaller-scale projects can utilize existing infrastructure. The high technology readiness further facilitates infrastructure scaling to accommodate potential large-scale SEC trade, leveraging existing regulatory frameworks and vast expertise^12,13^. Unlike ammonia or methanol, there is currently no globally established large-scale fossil-based hydrogen trade infrastructure comparable to existing fossil commodity markets. However, existing pipeline infrastructures could be retrofitted to enable hydrogen transport^1^. Sea-based liquid hydrogen transport is possible, but liquid hydrogen carrier ships are not expected to become widely available before the 2030 s^14^. In summary, the large-scale deployment of SECs will depend on the ability to produce them globally and to transport them using existing or adapted infrastructure. This development suggests a potential transformation of the global energy trading system, as new export regions may emerge, and raises critical questions regarding the future geography of global energy flows, the suitability and availability of transport infrastructure, and the types of SECs that will dominate long-distance trade.

The transport of SECs is included in numerous publications, using energy system models like Balmorel, PyPSA, Enertile, FINE, COMPETES, or ESDP, where PtX transport routes are typically aggregated as connections between model regions rather than representing individual ports or pipelines^15–22^. Publications with greater emphasis on the transport of SECs are often case studies focusing on specific bi-national energy trade routes^11,23–28^. While some studies consider multiple countries as potential exporters^29,30^, they typically remain limited to country-level resolution, overlooking intra-national energy transport, local infrastructure, and renewable electricity generation potentials. Additionally, the scope of transport means and SEC conversion options is often restricted. Other publications investigate multimodal energy transport between abstract, undefined energy exporting and importing regions, identifying optimal energy carrier and transport mode choices, but without consideration of local contexts^13,31–33^.

Based on the literature, we identify a research gap: existing studies on global PtX commodity trade and SEC transport are limited by aggregated spatial representations, narrow case-study perspectives, or restricted consideration of transport and conversion options. Most approaches neglect detailed intra-national renewable energy potentials, local infrastructure availability, and geographically explicit multimodal transport networks, while studies addressing transport optimization often rely on abstract regions without real-world spatial context. Consequently, there remains a lack of an integrated, high-resolution modeling framework that combines detailed infrastructure representation, multiple SEC pathways, and realistic global transport dynamics to comprehensively assess future large-scale SEC trade.

We develop a heuristic based on a mixed-integer optimization model to calculate the most cost-efficient transport routes and transport costs from 10,883 globally distributed energy export locations to the import countries Germany, Japan, China, India, and the United States by 2030, representing major prospective importers of renewable hydrogen products according to the Hydrogen Council^34^. Rather than assessing only readily available infrastructure, this study employs a forward-looking “what-if” perspective to identify infrastructure retrofit options and investment needs to realize future SEC trade potentials. The accompanying dataset includes global shipping and pipeline infrastructure, as well as geographically high-resolved hydrogen production and electricity generation costs from solar PV and onshore wind. Our methodological approach contributes to large-scale hydrogen transport network modeling, particularly in terms of computational tractability, spatial resolution, and infrastructure representation. Based on a literature review, we calculated transport and conversion costs as well as boil-off for the target year 2030 for seven different SECs (hydrogen, ammonia, methanol, synthetic natural gas, Fischer-Tropsch crude, Methylcyclohexane (MCH), and Dibenzyltoluene (DBT)) and four transport means (ship, truck, and existing and newly built pipelines).

Our analysis shows that existing pipeline infrastructure is the key driver of cost-efficient global trade in SECs, allowing Europe, North Africa, and Central Asia to remain competitive despite higher hydrogen production costs. Furthermore, the choice of transport carrier depends strongly on the transport distance and the availability of infrastructure: gaseous hydrogen is preferred for short pipeline-based transport, while ammonia, methanol, and methane become more economical for long multimodal supply chains involving road and maritime transport. Meanwhile, regions with high renewable energy potential are only attractive export locations if sufficient transport infrastructure, namely pipelines or ports, is available; otherwise, inland transport costs rise significantly. Retrofitting existing natural gas pipelines for hydrogen transport substantially lowers import costs and increases supply potential by avoiding costly conversion and reconversion steps. Finally, commodity prices and process conditions strongly influence carrier competitiveness: ammonia and methanol are gaining importance in scenarios where the market value of their conventional, fossil-based production in the importing country is taken into account, and liquid organic hydrogen carriers (LOHCs, represented here by DBT and MCH) become considerably more competitive when low-cost heat is available. These results aim to provide decision support for different stakeholders: the ability to calculate the most cost-efficient transport routes supports PtX plant investors in their choice of location and produced commodity. Policy makers can identify attractive export regions, key infrastructure, and necessary repurposing. Additionally, researchers and market analysts may utilize the open-access data, the model, and supply curves for in-depth investigations, such as regional case studies or the development of comprehensive market models.

## Results

Based on the analysis of seven scenarios summarized in Table 1, the following subsections discuss the main findings.

**Table 1:** Overview of applied scenarios

Scenario	Commodity at destination?	Retrofit of natural gas pipelines?	Free heat supply at all nodes?	Commodity prices considered?	Consider water availability?
H2-Retrofit	H_2_	Yes	No	No	No
H2-No-Retrofit	H_2_	No	No	No	No
All-Retrofit	Any	Yes	No	No	No
All-No-Retrofit	Any	No	No	No	No
All-Retrofit-Heat	Any	Yes	Yes	No	No
All-Retrofit-Prices	Any	Yes	No	Yes	No
All-Retrofit-Water	Any	Yes	No	No	Yes

Scenario explanation: All scenarios consider 10,883 equally distributed energy export locations around the globe. In three of these scenarios (“All” scenarios), we do not define which SEC is supplied to the final destination (hydrogen (gaseous G-H2 and liquid L-H2), ammonia (NH3), methanol (MeOH), synthetic methane (G-CH4, L-CH4), DBT, MCH, and FTC). In the other two scenarios (“H2” scenarios), gaseous hydrogen is set as the target commodity at the destination. Furthermore, three scenarios allow for the utilization of natural gas pipelines not only for synthetic methane but also for gaseous hydrogen, with associated retrofit costs (“Retrofit” scenarios). In the main paper, Germany is selected as the import destination. To demonstrate the broader applicability of our approach, results for Japan, China, India, and the United States as import destinations^34^ are provided in the Supplementary Figs. 1–8. Moreover, the underlying database covers all countries, enabling users to run these calculations without additional data collection by specifying the desired import country in the model configuration. We further investigate the impact of waste heat availability for conversion processes (All-Retrofit-Heat). As the model does not cover the end-use application of the SECs at the destination, supply costs are calculated on an energy content basis to ensure comparability across pathways. To acknowledge potential non-energetic, material uses of these commodities, commodity prices are included in the All-Retrofit-Prices scenario to compare their value at the destination. Finally, locations are assessed and restricted based on water availability in the All-Retrofit-Water scenario (Supplementary Note 2).

### Existing infrastructure shapes global SEC trade

**Fig. 1:**
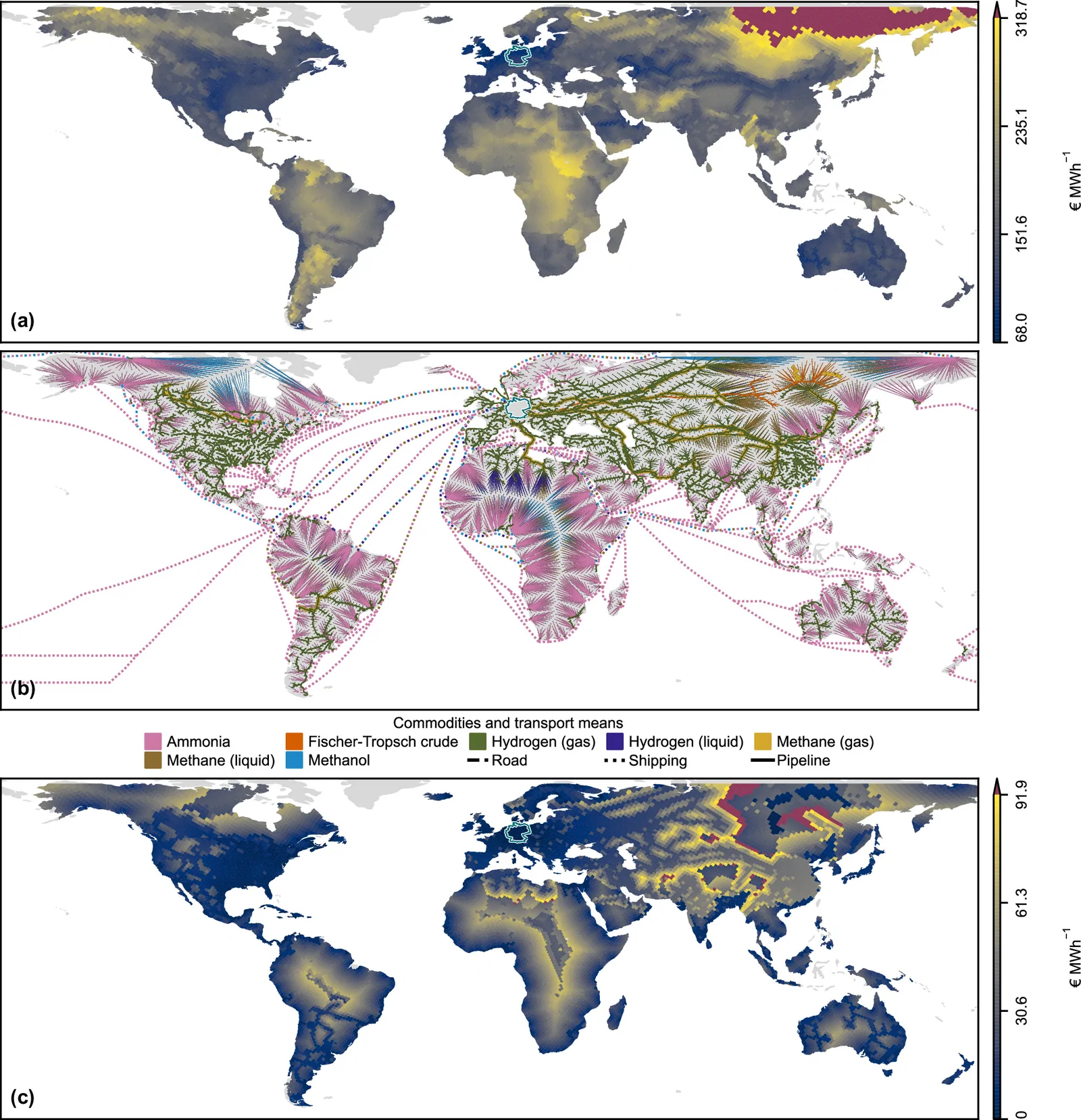
Supply costs, transport routes, and transport costs for synthetic energy carriers from global export regions to Germany under the All-Retrofit scenario. **a** Map of total supply costs including transport and conversion, showing that despite comparatively high hydrogen production costs, European regions remain competitive due to the availability of existing pipeline infrastructure and avoided conversions. **b** Most frequently selected transport modes and synthetic energy carriers across export locations, highlighting the strong role of pipelines for hydrogen transport from proximate regions, while ammonia, methanol, and methane are selected for longer, multimodal supply chains involving road and maritime transport. A high-resolution version of this map is available in Supplementary Fig. 10. **c** The transport cost component of total supply costs indicates that low costs can still be achieved from distant regions such as Patagonia or North America, where shipping dominates or combines effectively with pipeline infrastructure. Areas without nearby infrastructure rely on more costly road transport and additional conversion steps. A comparison of production, conversion, transport, and total supply costs is shown in Supplementary Fig. 9.

Our first key finding stems from the analysis of the All-Retrofit scenario. The results show that unfavorable hydrogen production costs in Europe are compensated with low-cost transport due to existing pipeline infrastructure and the avoidance of conversions and boil-off, achieving competitive supply costs (Fig. 1a) due to lower losses along the supply chain (Supplementary Note 1). This circumstance allows European production locations to compete with locations outside Europe, where renewable electricity generation conditions might be much more favorable. Available pipeline infrastructure or proximity to ports affects supply costs, which increase when road transport, with its associated conversions, is needed.

Our results further show the choice of transport mode, transport route, and energy carrier for all export locations (Fig. 1b). If pipeline infrastructure is available in the exporting region, we see its utilization to transport gaseous hydrogen either directly to Germany or to the coast, with subsequent conversion to ammonia and shipping to Germany. With increasing distance from the pipeline network that has to be covered through road transport, liquid hydrogen (e.g., MENA) or liquid methane (e.g., Mongolia, Northern Canada, MENA) are produced and transported by truck, followed by regasification and pipeline transportation. Compared to hydrogen, methane has the advantage of cheaper road and pipeline transport (Supplementary Table 5), and is used as soon as the combined road and pipeline distances are far. Liquid hydrogen is used for medium road distances since the conversion from gaseous to liquid hydrogen is more efficient than the conversion to methane. Gaseous hydrogen implies high road transport costs and is only used near close pipelines. If no pipeline infrastructure is nearby, depending on the distance to the coast, either ammonia or methanol is produced and transported via road to the next port, then shipped to Germany. Methanol has lower road transport costs but higher conversion costs (Supplementary Table 3) than ammonia, making it attractive for inland regions with long road transport to the coast, without pipeline transport (Fig. 1a). In addition to cost jumps resulting from changes in transported commodities, Fig. 1c shows that transport costs can remain notably low despite great transport distances, e.g., when cost-efficient shipping is the only required mean of transport (e.g., Patagonia) or shipping can effectively be combined with existing pipeline infrastructure (e.g., North America). Our results indicate that from the German point of view, European neighbors should be the first address for energy trade since import costs are low compared to other countries due to proximity and existing transport infrastructure. Next, countries in Northern Africa, Central Asia, and the Middle East connected to the European oil or natural gas pipelines allow for low-cost imports. Countries and regions with high renewable electricity generation potential without connection to the European natural gas and oil infrastructure could supply SECs beyond hydrogen, since conversion is needed for cost-efficient ship transport. These regions and countries include North America, Patagonia, coastal areas of Africa, and Australia. Low inland supply costs depend on the availability of pipeline infrastructure in these regions. However, with increasing distances between Germany and the producing countries, other SEC-demanding countries might have the advantage of achieving lower import costs due to closer proximity. Supplementary Figs. 9,10 are high-resolution versions of these figures.

**Fig. 2:**
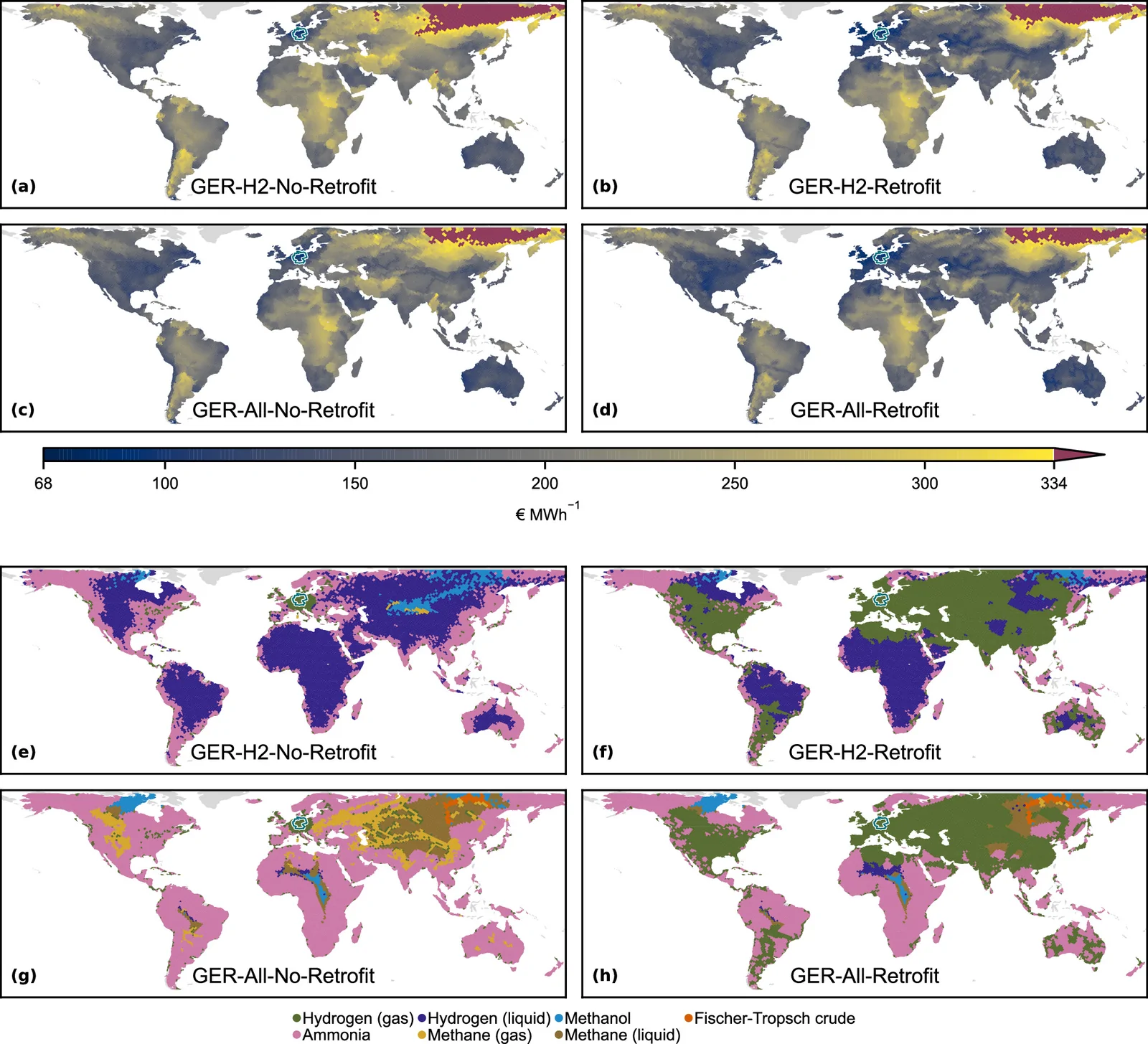
Impact of pipeline retrofitting and target commodity specification on energy supply costs and synthetic energy carrier (SEC) selection. **a–d** Total supply costs from global export regions to Germany. **e–h** Preferred SEC produced under the four scenarios. **a, e** GER-H2-No-Retrofit. **b, f** GER-H2-Retrofit. **c, g** GER-All-No-Retrofit. **d**, **h** GER-All-Retrofit. Allowing pipeline retrofitting significantly reduces supply costs, especially in Europe, North Africa, and Central Asia. When the imported commodity is fixed to gaseous hydrogen **e, f** ammonia dominates coastal routes, while liquid hydrogen is used in inland areas. In scenarios with flexible commodity choice **g, h** ammonia and methanol are favored depending on road distance to ports, and methane or Fischer-Tropsch crude are selected when pipeline infrastructure is available or when long road transport is required. Gaseous hydrogen is produced when pipeline networks can be retrofitted.

A comparison of results for the first four scenarios is presented to analyze the impact of retrofitting the natural gas infrastructure and the choice of target commodity at the destination. The observation that costs are lower for regions with existing pipeline infrastructure in scenarios allowing for their reuse for hydrogen transport (Fig. 2b, d) compared to scenarios without reuse (Fig. 2a, c) is unsurprising. This is particularly apparent for locations within Europe and adjacent regions, and underlines the importance of preparing the natural gas infrastructure to transport gaseous hydrogen. Not allowing for the reuse requires the transport of hydrogen derivatives, which results in higher costs due to conversions.

In scenarios where the delivered commodity to Germany remains unspecified, average costs are lower as no conversion to gaseous hydrogen is necessary at the destination (Fig. 2c, d), and the variety of used SECs is much broader (Fig. 2g, h). If gaseous hydrogen is the target commodity, mainly ammonia with low conversion and reconversion costs to gaseous hydrogen is applied for coastal regions, whereas inland regions lacking pipeline infrastructure start with liquid hydrogen due to lower road transport costs than ammonia (Fig. 2e, f).

If the target commodity is not defined and the existing infrastructure is not hydrogen-ready (Fig. 2c, g), ammonia plays the most crucial role due to low conversion, road transport, and shipping costs. Locations with higher distances to coastal regions use methanol if no pipeline infrastructure exists and shipping is needed in a subsequent transport step. Inland areas with available natural gas infrastructure (Central Asia, North America, and North Africa) utilize gaseous (for pipeline) and liquid (for road and shipping) methane. Finally, FTC is used if only oil pipelines exist in combination with longer road distances.

### Infrastructure availability affects attractive locations

**Fig. 3:**
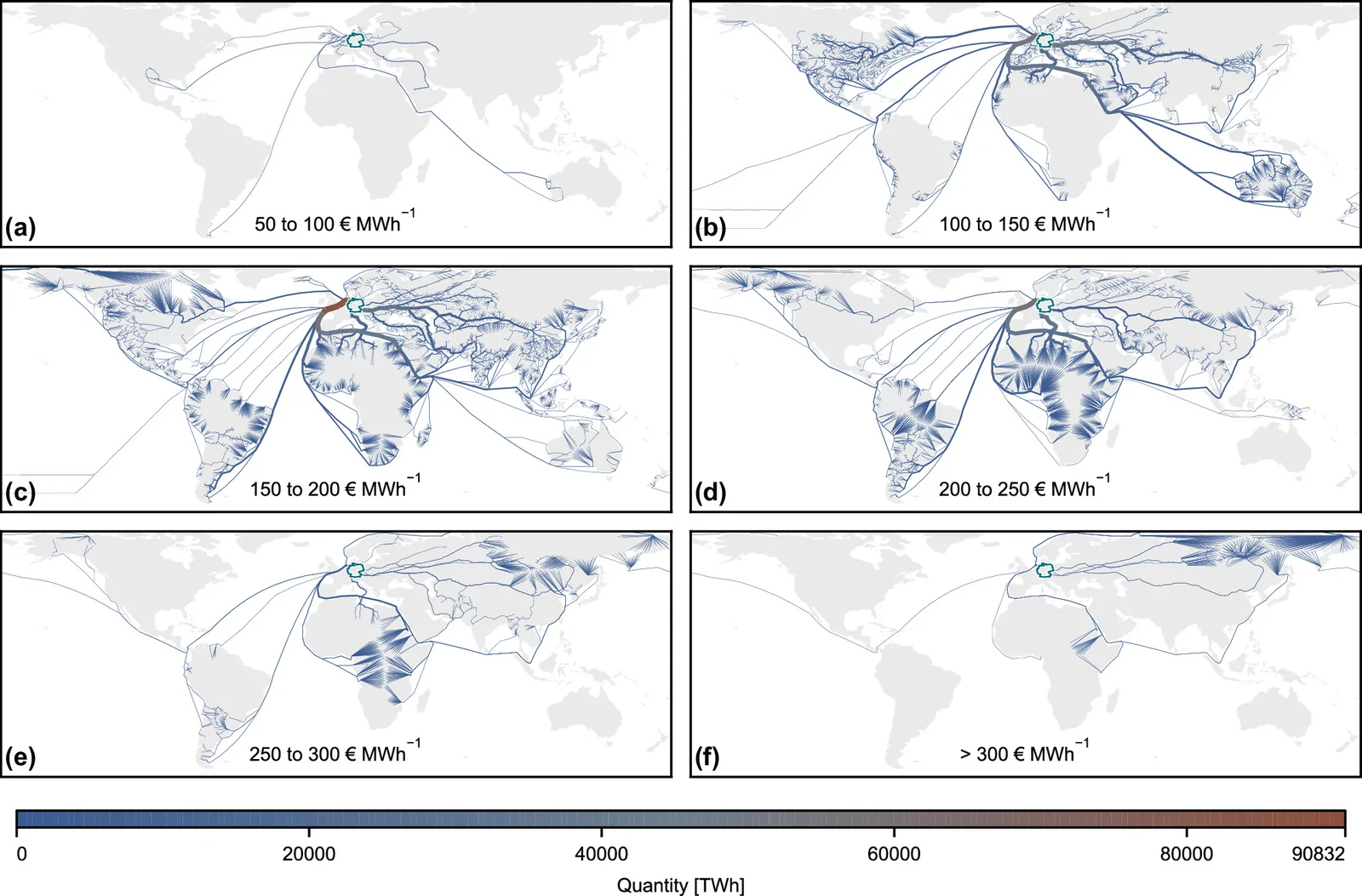
Quantity-weighted transport routes in the GER-All-Retrofit scenario, categorized by total supply costs. **a** 50 to 100 € per MWh. **b** 100 to 150 € per MWh. **c** 150 to 200 € per MWh. **d** 200 to 250 € per MWh. **e** 250 to 300 € per MWh. ** f** More than 300 € per MWh. Central Asia, the Middle East, and Northern Africa combine low supply costs with high production potentials, making them prime candidates for future synthetic energy carriers imports. Australia and North America offer competitive supply options, mainly sea-based routes. In contrast, inland regions without infrastructure exhibit higher supply costs and are less likely to contribute significantly to international trade.

Total supply costs are insufficient to assess transport routes and potential export regions, as SEC production facilities are developed only if sufficient quantities can be produced at low supply costs. To determine both, we use the total supply costs in Fig. 1a and weight each location’s most cost-efficient transport route by the location’s potential export quantity. We cluster the locations by their total supply costs into six cost ranges and sum potential export quantities of shared infrastructure for each cluster (Fig. 3).

Next to low total supply costs, Central Asia, the Middle East, and Northern Africa show high SEC production potentials, underlining their favorable properties for future imports to Germany (Fig. 3a). Combined with the existing transport infrastructure, these regions could secure the future supply of SECs to Germany. Other locations with low total supply costs, like Australia and North America, are also attractive (Fig. 3b), but the sea-based transport of SECs is less tied to infrastructure, allowing global supply and opening international competition (Supplementary Figs. 1–8). Hinterland locations often show high renewable potential but at higher total supply cost since transport infrastructure is unavailable (Fig. 3c–f). These locations will probably not be utilized due to the sufficient potential of other locations, but could be used for local supply.

**Fig. 4:**
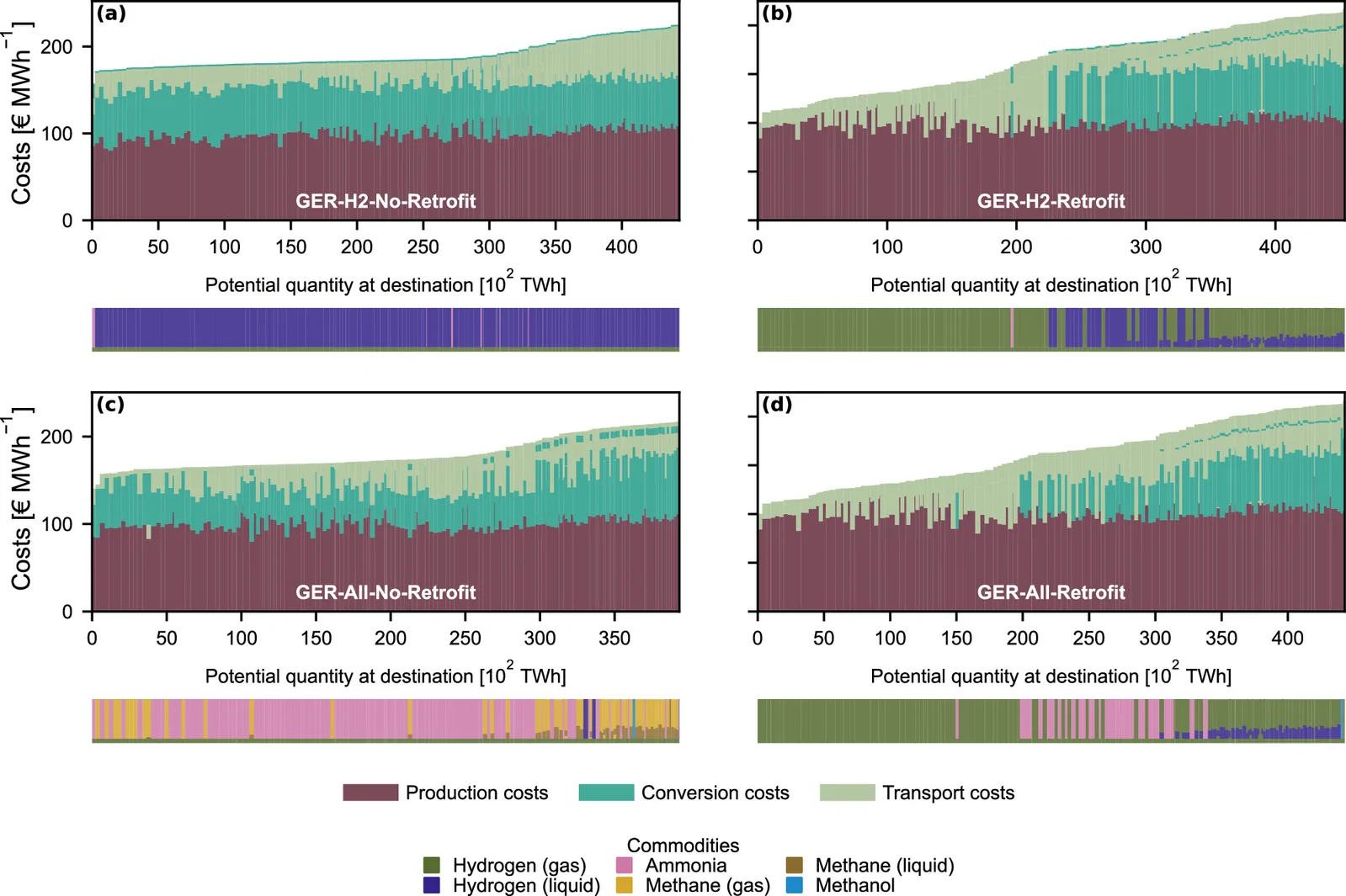
Supply curves and commodity bands for synthetic energy carrier import from Algeria to Germany under four scenarios. Supply curves display the cumulative quantities and associated costs of imports from Algeria, allowing assessment of how infrastructure availability and target commodity specification affect supply potential. Below the supply curves, commodity bands show the transported commodities and their share of the total transport distance. In scenarios enabling retrofitting existing pipeline infrastructure **b, d** supply costs are significantly lower and quantities are higher, as hydrogen is transported in gaseous form in the majority of cases. In contrast, scenarios without retrofitting **a, c** lead to sharp cost increases and reduced supply volumes due to additional conversion and reconversion requirements.

Supply curves give insights into export regions’ cost and quantity structure, allow comparison between the different export locations, and show in which order locations should be exploited to achieve the most cost-efficient supply. Furthermore, they will enable the impact assessment of various scenarios on costs and quantities of the region, and changes on the left-hand side of each supply curve are especially of interest since these potentials would be exploited first. Countries with pipeline infrastructure connected to the destination, such as Algeria, profit from low transport costs and the direct transport of hydrogen, visible in the All-Retrofit (Fig. 4d) and H2-Retrofit (Fig. 4b) scenarios of the Algeria-to-Germany supply curve. However, as soon as existing pipeline infrastructure is unavailable (All-No-Retrofit (Fig. 4c) and H2-No-Retrofit (Fig. 4a) scenarios), costs increase significantly, especially for locations that achieve low total supply costs in the All-Retrofit setting due to necessary conversions and reconversions. Besides increasing costs, quantities decrease due to efficiency losses from conversions and reconversions.

**Fig. 5:**
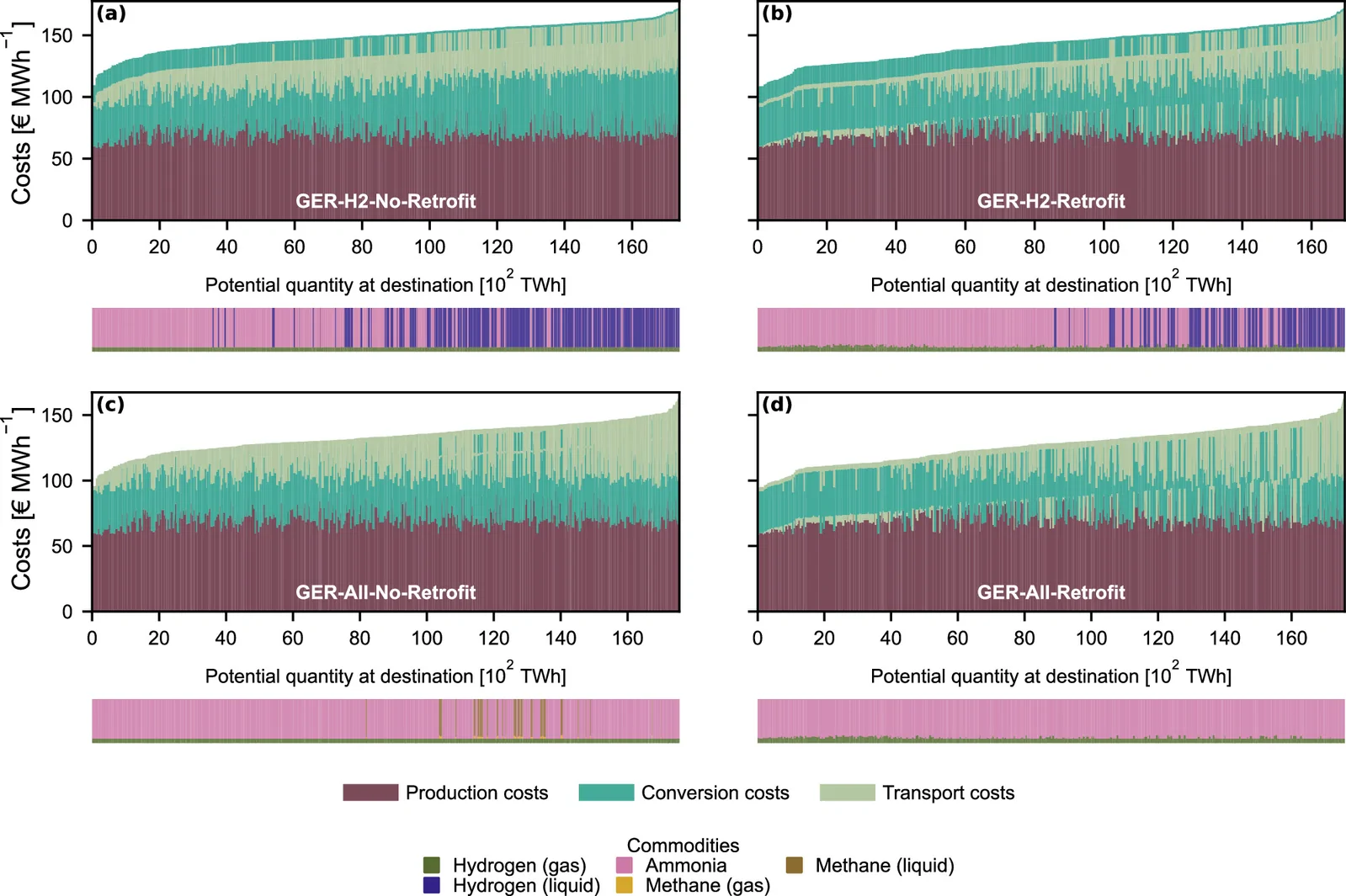
Supply curves and commodity bands for synthetic energy carrier import from Australia to Germany under four scenarios. In contrast to pipeline-connected regions, Australia’s supply curves show relatively small differences between scenarios, as long-distance transport requires conversions to hydrogen derivatives regardless of infrastructure retrofitting. Nonetheless, slight cost advantages are visible in the retrofit **b, d** scenarios compared to the scenarios forbidding pipeline retrofitting **a, c** due to the use of domestic pipelines within Australia for initial hydrogen transport before conversion.

If no pipeline connection between the exporting country and the destination exists, supply curves are less affected by changes in the availability of infrastructure. In such cases, conversions to hydrogen derivatives are necessary to bridge large road and shipping distances, reducing the impact of infrastructure availability. Such a behavior can be seen by looking at the Australia-to-Germany supply curve (Fig. 5). However, even here, a slight cost increase can be seen comparing the left-hand sides of the different scenarios, since domestic pipeline transport of hydrogen in Australia plays a role in the Retrofit (Fig. 5b, d) scenarios.

### Different infrastructure settings create carrier niches

**Fig. 6:**
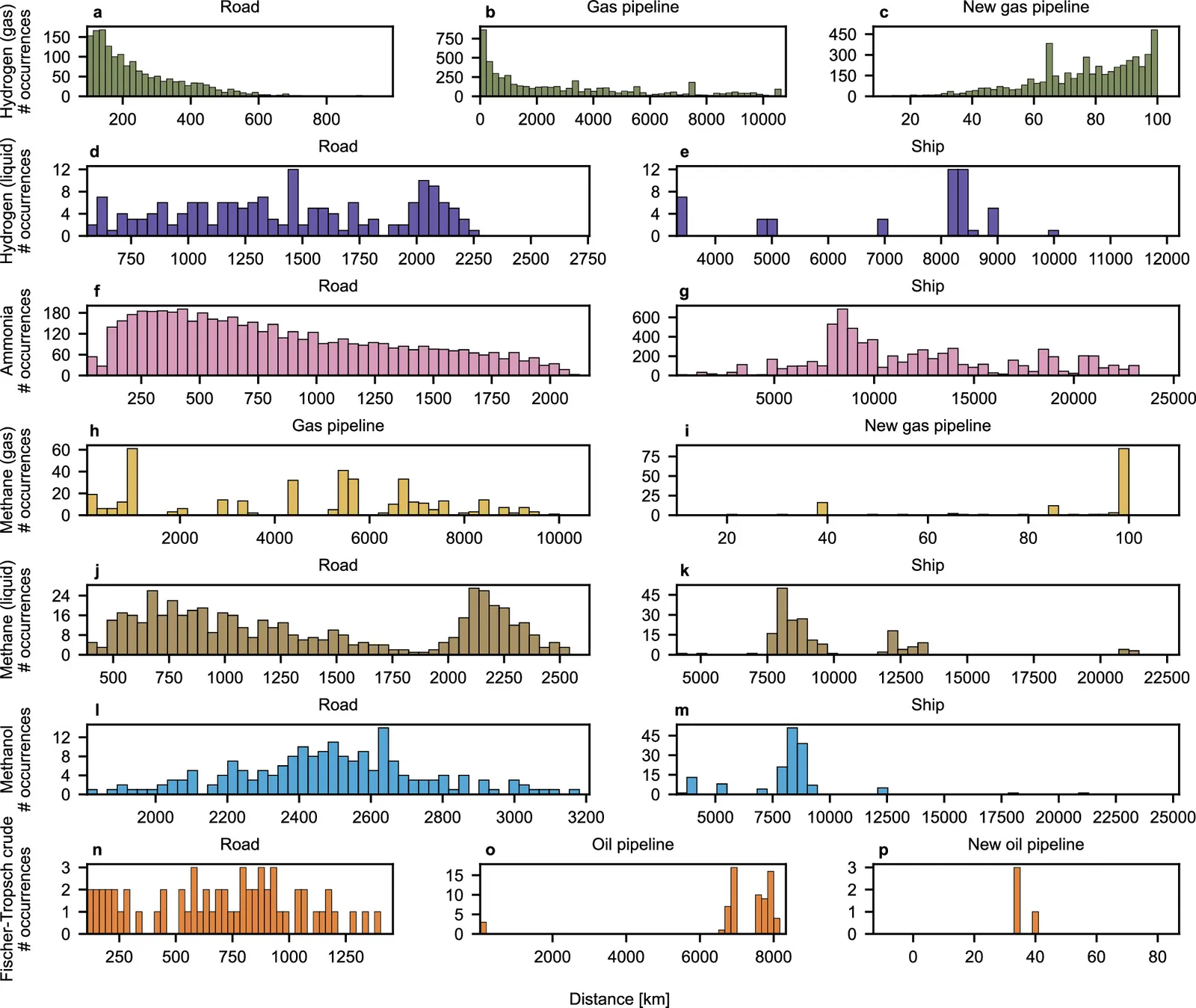
Transport distances and selected transport means for various synthetic energy carriers under the All-Retrofit scenario. Histograms show the distribution of transport mode deployment across transport distances for each synthetic energy carrier. Gaseous hydrogen is primarily transported via pipeline **b, d** or short-range trucking ( < 500 km) **a**, while liquid hydrogen covers up to 2250 km of road transport (**d**) with subsequent shipping **e**. Ammonia is used where moderate road transport to ports (**f**) is followed by shipping **g**, as it benefits from low synthesis and conversion costs. Depending on pipeline availability, methane is transported in gaseous **h, i** or liquefied **j, k** form. Methanol becomes favorable for remote inland regions with long road distances (**l**) and subsequent shipping (**k**) due to its lower transport costs despite higher synthesis costs compared to ammonia. Fischer-Tropsch crude is selected for multimodal routes with oil pipeline usage **o, p** and road transport to the pipeline (**n**) where long transport distances justify its high production costs.

Returning to the All-Retrofit scenario, Fig. 6 analyzes the selection of SECs and transport means as a function of transport distance. The transport of hydrogen is possible via truck, ship, and pipeline. However, due to the low volumetric energy density and energy-intensive liquefaction, only transport via pipeline is economically feasible for long distances. Road and new gas pipelines allow transport to existing pipelines. However, new gas pipelines are limited to 100 km distances, hence the abrupt limit at 100 km (Fig. 6, Hydrogen (Gas)). While gaseous hydrogen road transport is used for distances of up to 500 km, longer distances of up to 2000 km are covered with liquid hydrogen trucks, as the energy savings during transport outweigh the energy losses from liquefaction (Fig. 6, Hydrogen (Liquid)).

Lower conversion costs significantly advantage ammonia over other SECs (Supplementary Table 3). The combination of lower conversion and moderate transport costs makes ammonia a prioritized SEC as soon as pipeline infrastructure is not available. The main application of ammonia is at locations where distances up to 2000 km have to be covered via road transport to the next port, with subsequent shipping over a broad range of distances (Fig. 6, Ammonia). Liquid methane is used for road transport, with subsequent shipping or pipeline transport of gaseous methane (Fig. 6, Methane (Gas) and Methane (Liquid)). Methanol closes the gap if long-distance onshore transport is necessary to reach a port, but no pipeline infrastructure exists. It has lower road and shipping transport costs than ammonia and liquid methane, but its production is more expensive. This circumstance leads to the utilization of methanol if shipping transport is necessary and preceded by long-distance ( > 1750 km) road transport (Fig. 6, Methanol). The high conversion costs make FTC only viable if large distances must be covered by different means of transport, including existing oil pipelines (Fig. 6, FTC).

### LOHC viability driven by heat available for dehydrogenation

**Fig. 7:**
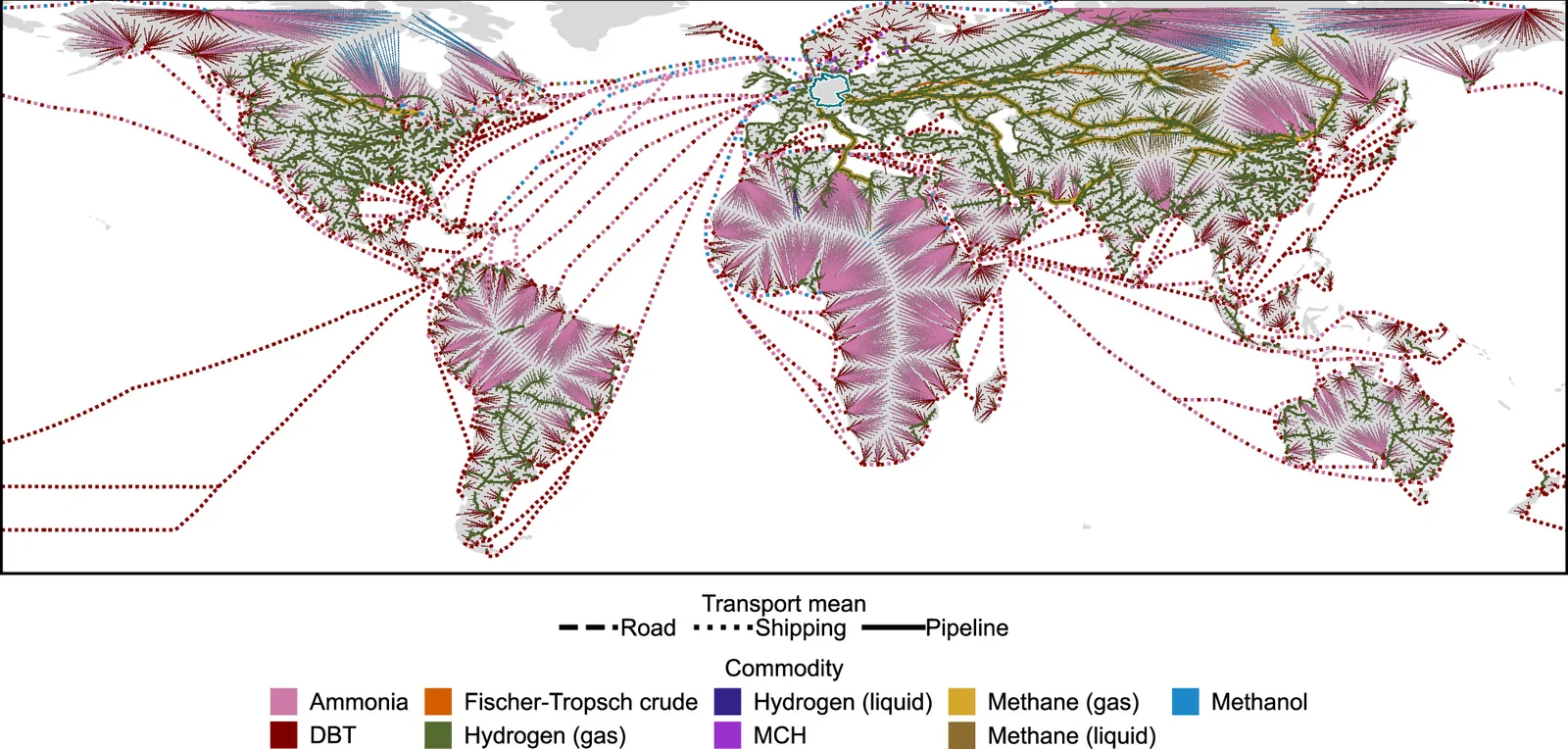
Routes and selected synthetic energy carriers in the GER-All-Retrofit-Heat scenario. The routes show that Dibenzyltoluene predominantly replaces short-distance ammonia road transport with subsequent shipping. Methylcyclohexane is rarely used due to the higher electricity demand of the dehydrogenation process.

LOHCs are under investigation as intermediate hydrogen carriers that use specific chemical solvents to bind hydrogen during hydrogenation, enabling cost-efficient hydrogen transport due to their increased volumetric energy density and liquid form, with subsequent dehydrogenation at the import location to release hydrogen. However, the dehydrogenation of LOHCs is very heat-intensive: The efficiency of the process for DBT is about 65%, and without a low-cost heat supply, autothermal processes are necessary (Supplementary Method 4). The All-Retrofit-Heat scenario assumes a free heat supply at the start location, all ports, and the destination. This scenario leads to using DBT for routes containing shipping transport. Figure 7 shows all the taken routes and applied SECs in the All-Retrofit-Heat scenario. In contrast to Fig. 1b, DBT instead of ammonia is chosen for short-distance road transport and subsequent shipping. DBT, in comparison to ammonia, offers the advantage of a very efficient conversion from hydrogen. Although the hydrogenation of MCH is less expensive than that of DBT, and transportation costs by ship and truck are comparable, MCH is rarely utilized as the electricity demand for hydrogen release is significantly higher. The use of LOHCs in this scenario alone highlights the central challenge of LOHCs: without a free or low-cost heat supply, they are at an economic disadvantage compared to other SECs when used to supply hydrogen.

### Market-value adjustment favors high-revenue energy carriers

**Fig. 8:**
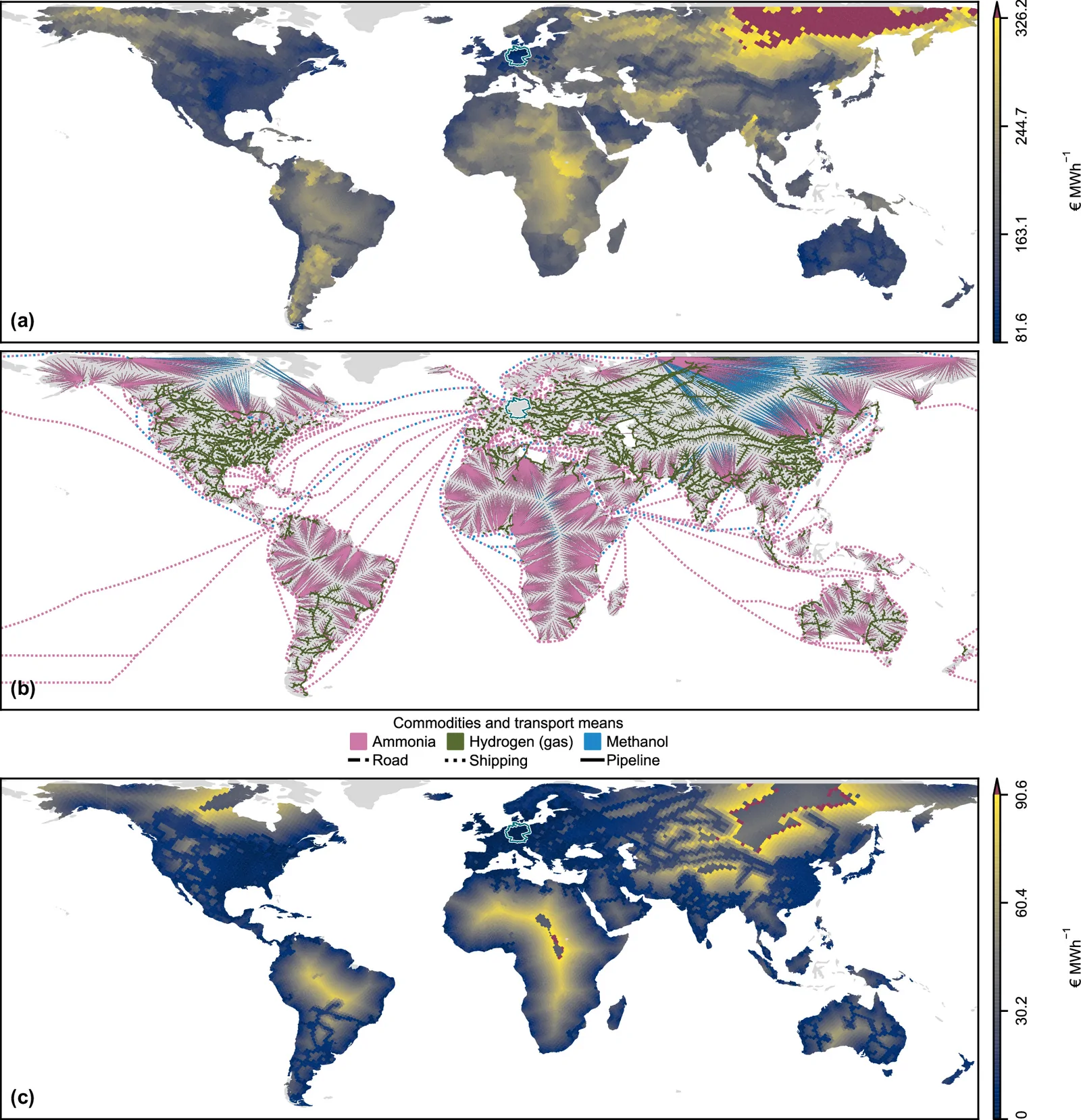
Supply costs, transport routes, and transport costs for synthetic energy carriers from global export regions to Germany under the GER-All-Retrofit-Prices scenario. **a** Total supply costs. **b** Selected routes. **c** Transport costs. In this scenario, high-value synthetic energy carriers dominate the solution since their commodity prices are considered when calculating the most cost-efficient solutions. Especially ammonia becomes more significant, replacing liquid hydrogen and methane in inland regions. Methane and Fischer-Tropsch crude are replaced by methanol even when pipeline infrastructure exists.

By accounting for differences in the market value of the considered energy carriers in the import region through exogenous, carrier-specific commodity prices (Supplementary Method 6), the GER-All-Retrofit-Prices scenario extends the pure supply-cost minimization approach. Specifically, the total supply costs at the destination are reduced by the respective commodity price, thereby representing carrier-specific revenue streams and increasing the attractiveness of energy carriers with higher market values. Under this net-cost perspective, high-value SECs, such as ammonia and methanol, dominate all other SECs except gaseous hydrogen (Fig. 8b). In the case of gaseous hydrogen, low-cost pipeline supply and the avoidance of conversion costs offset its lower commodity price compared to ammonia and methanol. The economic advantage of ammonia and methanol is sufficiently pronounced that, even in the GER-All-Retrofit scenario where pipeline transport is available, pipeline-based supply routes from northern Africa to Europe or from Siberia to Europe are replaced by road transport and shipping of ammonia and methanol. Consequently, these routes may exhibit higher gross total supply costs than alternative SEC pathways, but they become more attractive once the higher commodity-price-related revenues of ammonia and methanol are taken into account.

**Fig. 9:**
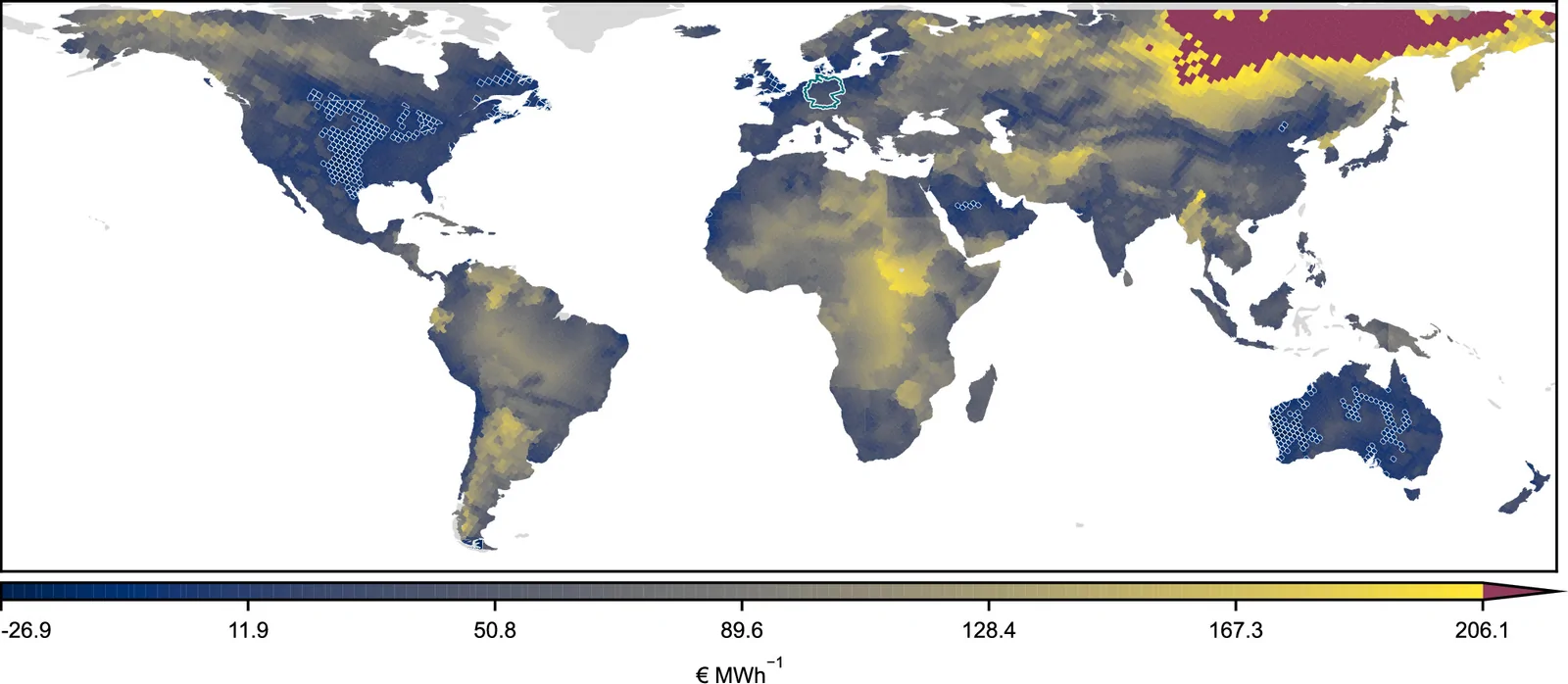
Delta between total supply costs of synthetic energy carriers and costs of fossil production in the GER-All-Retrofit-Prices scenario. A negative objective function value (white outlined locations) is achieved if the total supply costs of synthetic energy carriers are lower than those of their fossil counterparts, including fossil energy carrier price, conversion costs, and CO_2_ pricing costs. The map shows that only a few locations achieve a negative objective function value, indicating that at the assumed fossil energy and CO_2_ prices, low-carbon synthetic energy carriers are not highly competitive.

Commodity prices are based on fossil energy carriers, namely natural gas and crude oil, conversion costs to the target commodities, and costs occurring based on the emitted CO_2_ of the conversion processes (Supplementary Method 6). Since the assessed SECs are modeled to be produced in accordance with the European regulation on renewable fuels of non-biological origin, the SECs can be classified as low-carbon, and the respective objective function value interpreted as the profits or losses of a low-carbon SEC supply. Hence, SECs from locations with negative objective function values are more attractive than fossil energy carriers, as their total supply costs are lower than those of their fossil counterparts. However, this is true only for a few locations, with the majority in North America and Australia, and mainly for the import of Ammonia (Fig. 9). To increase the number of locations with lower SEC total supply costs than total costs of their fossil counterparts, SEC costs need to decrease or the total costs of fossil energy carriers need to increase. Especially the latter can be controlled through CO_2_ pricing mechanisms, and investments into SEC production could be triggered if this mechanism is ambitious and reliable.

## Discussion

We present an integrated approach to assess future global transport of SECs, considering global production sites, multiple energy commodities, their interconversion, and various means of transport to calculate the most cost-efficient routes. The model features highly resolved infrastructure datasets to consider global gas and oil pipelines and ports, and can be applied to any combination of import and export locations worldwide. In addition, we supplied a mixed-integer optimization problem, which can assess the optimality of our solutions and the computational advantages of the heuristic. It is publicly available on GitHub, along with comprehensive global input data.

We demonstrate the model’s functionality through case studies examining the supply of SECs from 10,883 locations worldwide to Germany, the US, China, Japan, and India. Analyzing seven scenarios with Germany as the import region in our main paper, we identify conditions under which specific SECs constitute efficient energy transport options. Our results underscore that the availability of transport infrastructure is decisive in enabling cost-efficient transport. For instance, Europe’s extensive pipeline network allows for a low-cost supply of gaseous hydrogen, which is competitive with derivative imports from regions with lower renewable electricity costs. Yet, distant exporters can also be cost-effective if located near coastlines (e.g., Patagonia) or have a well-established, retrofit-ready pipeline infrastructure (e.g., North America, Australia). Without retrofitting natural gas pipeline infrastructure, low-cost hydrogen may be limited to a few coastal locations while the production of derivatives becomes more attractive. For ship-based energy transport, our results support the utilization of ammonia. SECs with higher conversion costs from hydrogen, namely methanol and methane, are selected only for long transport distances, as their transport is cheaper than that of hydrogen and ammonia. These results align with recent research^11,20,21,24,29,35^. Liquid hydrogen is used when shipping is required and hydrogen is the demanded commodity at the import location. DBT and MCH are only utilized in scenarios with costless heat available. In this case, especially short-distance road and shipping routes use these LOHCs. Finally, when considering commodity prices in calculating total supply costs, ammonia and methanol dominate other SECs because their higher prices significantly reduce total supply costs. However, compared to their fossil counterparts, they are still more expensive in most cases, which could spark a discussion about setting more ambitious CO_2_ prices to encourage the replacement of fossil fuels.

The modeling results suggest several policy implications. First, countries should accelerate the retrofit of natural gas infrastructure to enable hydrogen transport. This will allow a low-cost supply of SECs and the initiation of a SEC market since such requires the transport of commodities. Second, pipeline networks should be extended to connect major demand and supply locations, e.g., Europe with neighboring countries in Northern Africa, the Middle East, and Central Asia. Third, terminals must be retrofitted to allow the import of sufficient quantities of SECs, mainly ammonia and methanol. Fourth, the selection of ammonia and methanol instead of hydrogen for maritime trade encourages the concentration on higher-value chain products for sea-based imports that require the development of a basic chemical feedstock industry in exporting regions.

The developed approach is subject to the following limitations. Our analyses present a snapshot for 2030 with critical input parameters of transport infrastructure and conversion plants, such as conversion efficiencies, CAPEX, and OPEX, based on a thorough literature review. Some of these technologies have a low technology readiness level, and their availability and development by 2030 remain uncertain. For example, only one demonstration carrier, one import, and one export terminal exist worldwide in the case of liquid hydrogen. Other key processes, including hydrogen liquefaction, ammonia cracking, LOHC dehydrogenation, and DAC, are currently available at smaller scales but have yet to achieve industrial-scale implementation^36–38^. However, sensitivity analyses of technology parameters were omitted to maintain focus and clarity in the presented results. Greenhouse gases are not considered, but would enrich the analysis and help derive more accurate carbon footprints, which could further support the analysis of carbon pricing mechanisms. Our global scope further limits the possibility of including more detailed transport and conversion data, such as capacities of ports, pipelines, and ships, loading and unloading processes, waste heat availability in ports, canal and port fees, or economies of scale for conversion plants. Compared with hydrogen supply models using dynamic time-series optimization, our approach cannot assess infrastructure operation and, therefore, cannot answer questions about temporal aspects such as loading and unloading, storage, or infrastructure occupancy. This limitation is necessary to handle the high spatial resolution of production and infrastructure data. Nevertheless, we see significant potential to combine the two modeling approaches. For example, our approach could be used to analyze distinct locations and provide data, such as supply curves, for energy system models with a regional focus, models analyzing the crowding-out effects of conventional energy carriers, or models identifying congestion and bottlenecks in transport infrastructure. Road transport routes and new pipeline segments are calculated using as-the-crow-flies distances adjusted by a detour factor, since more detailed information like geographical or street map data would substantially increase computational complexity. However, this additional effort would enable the consideration of larger infrastructure investments.

## Methods

The applied methodology in this work aims to solve a mixed-integer optimization problem to find the most cost-efficient energy transport route from pre-defined starting points to desired locations. The input data includes hydrogen production and electricity generation costs as well as hydrogen production potentials for each cell of the grid, techno-economic data for all conversion units and transport modes, and infrastructure data of ports and oil and gas pipelines. Based on these data, the model decides on transport routes, transport modes, and conversions to achieve the most cost-efficient transport route. Considering the overwhelming number of conversion and transport options in spatially highly resolved real-world applications, a closed solution of the previously defined mixed-integer problem is out of scope, even for the most advanced solver and computational resources. Therefore, we developed an iterative branching algorithm, which allows for a good approximation of the initial problem. The modeling of the renewable profiles and the calculation method of the costs of hydrogen production, electricity generation, conversion, transport, and commodity prices are described in the Supplementary Methods 2–6. An illustration of the method is given in the Supplementary Fig. 11.

### General problem

The following subsection provides the mixed-integer optimization problem, and Table 2 provides the required nomenclature. Given a set of commodities $c\in {C}_{{n}^{s}}^{s}$ at any start node ${n}^{s}\in N$, the goal of the developed approach is to find a cost-optimal conversion and transport path such that the total costs for delivering a SEC to one node of the subset of potential end nodes ${n}^{e}\in {N}^{e}$ are minimized. In this context, multiple target commodities $c\in {C}_{{n}^{e}}^{e}$ for any node of a specific target region ${n}^{e}\in {N}^{e}$ can be specified, which might be either directly delivered via multiple transport systems or converted from multiple intermediate transport commodities, which themselves are allowed to be transported by multiple means. In a closed optimization, this problem can be formulated as a mixed integer problem with Eqs. (1–9):

1 $$\min {\sum}_{{n}^{e}\in {N}^{e}}{\sum}_{c\in {C}_{{n}^{e}}^{e}}{u}_{{n}^{s},{n}^{e},c}$$

2 $$s.t\begin{array}{cc}\frac{{u}_{{n}^{s},n,{c}^{{\prime} }}+{\vartheta }_{{c}^{{\prime} },c}^{{CC}}}{{\eta }_{{c}^{{\prime} },c}}-{u}_{{n}^{s},n,c}\le \left(1-{x}_{n,{c}^{{\prime} },c}\right)*{BigM} & \forall n\in N,c\in C,(c{\prime},c)\in {\varOmega }_{n}^{C}\end{array}$$

3 $$\frac{{u}_{{n}^{s},n{\prime},c}+{\vartheta }_{{n}^{{\prime} },n,c,t}^{{gTC}}}{{\eta }_{t}}-{u}_{{n}^{s},n,c}\le \left(1-{x}_{{n}^{{\prime} },n,c,t}\right)*{BigM} \\ \forall n\in N,c\in C,t\in {T}^{g},(n{\prime},n)\in {\varOmega }_{t,c}^{g}$$

4 $${u}_{{n}^{s},n{\prime},c}+{\vartheta }_{{n}^{{\prime} },n,c,t}^{{aTC}}-{u}_{{n}^{s},n,c}\le \left(1-{x}_{{n}^{{\prime} },n,c,t}\right)*{BigM} \\ \forall n\in N,c\in C,t\in {T}^{a},(n{\prime},n)\in {\varOmega }_{n,c,t}^{a}$$

5 $$\begin{array}{cc}\begin{array}{c}{\sum }_{\left({n}^{{\prime} },n\right)\in {\varOmega }_{t,c}^{g}}{x}_{{n}^{{\prime} },n,c,t}+{\sum }_{\left({n}^{{\prime} },n\right)\in {\varOmega }_{n,t,c}^{a}}{x}_{{n}^{{\prime} },n,c,t}+{\sum }_{\left(n,{c}^{{\prime} },c\right)\in {\varOmega }_{n}^{C}}{x}_{n,{c}^{{\prime} },c}=\\ {\sum }_{\left(n,{n}^{{\prime} }\right)\in {\varOmega }_{t,c}^{g}}{x}_{n,n{\prime},c,t}+{\sum }_{\left(n,{n}^{{\prime} }\right)\in {\varOmega }_{n,t,c}^{a}}{x}_{n,{n}^{{\prime} },c,t}+{\sum }_{\left(n,c,{c}^{{\prime} }\right)\in {\varOmega }_{n}^{C}}{x}_{n,c,c{\prime} }\end{array} \\ \begin{array}{c}\forall n\in N/\{{n}^{s},{n}^{e}\},c\in C\\ ,t\in T\end{array}\end{array}$$

6 $$\begin{array}{cc}{\sum }_{t\in T}{\sum }_{c\in C}{\sum }_{({n}^{{\prime} },n)\in ({\varOmega }_{t,c}^{g}\cup {\varOmega }_{n,t,c}^{a})}{x}_{{n}^{{\prime} },n,c,t}+{\sum }_{(c{\prime},c)\in {\varOmega }_{n}^{C}}{x}_{n,{c}^{{\prime} },c}\le 1 & \forall n\in N\end{array}$$

7 $$\begin{array}{cc}{\sum }_{t\in T}{\sum }_{c\in C}{\sum }_{(n,{n}^{{\prime} })\in ({\varOmega }_{t,c}^{g}\cup {\varOmega }_{n,t,c}^{a})}{x}_{n,{n}^{{\prime} },c,t}+{\sum }_{(c,c{\prime} )\in {\varOmega }_{n}^{C}}{x}_{n,c,{c}^{{\prime} }}\le 1 & \forall n\in N\end{array}$$

8 $${\sum }_{c\in {C}_{{n}^{s}}^{S}}{\sum }_{t\in T}{\sum }_{\left({n}^{s},{n}^{{\prime} }\right)\in ({\varOmega }_{t,{c}^{s}}^{g}\cup {\varOmega }_{{n}^{s},t,{c}^{s}}^{a})}{x}_{{n}^{s},{n}^{{\prime} },{c}^{s},t}+{\sum }_{\left({c}^{s},{c}^{{\prime} }\right)\in {\varOmega }_{{n}^{s}}^{C}}{x}_{{n}^{s},{c}^{s},c{\prime} }=1$$

9 $${\sum }_{t\in {T}^{{gr}}}{\sum }_{c\in {C}_{{n}^{e}}^{e}}{\sum }_{\left({n}^{{\prime} },{n}^{e}\right)\in ({\varOmega }_{t,c}^{g}\cup {\varOmega }_{{n}^{e},t,c}^{a})}{x}_{{n}^{{\prime} },{n}^{e},c,t}+{\sum }_{\left({c}^{{\prime} },c\right)\in {\varOmega }_{{n}^{e}}^{C}\bigwedge c\in {C}_{{n}^{e}}^{e}}{x}_{{n}^{e},{c}^{{\prime} },c}=1$$

**Table 2:** Nomenclature of indexes and sets, parameters, and variables

Indexes and sets	
$n,{n}^{{\prime} }\in N$	Nodes of the transport system
${n}^{s},{n}^{e}\in N$	Start node (${n}^{s}$)
${n}^{e}\in {N}^{e}$	Subset of potential end nodes $({n}^{e})$ (e.g., nodes of a target country)
$c,{c}^{{\prime} }\in C$	Commodities for transport and conversion
t∈T	Transport systems
$t\in {T}^{g}$	Subset of transport systems based on a graph for modeling existing pipelines and shipping routes
$t\in {T}^{a}$	Subset of transport systems based on an air distance point-to-point connection for modeling new pipelines and road transport
$(c{\prime},c)\in {\Omega }_{n}^{C}$	Set of edges for modeling the available conversion processes at node n from $c{\prime}$ to c
$(n{\prime},n)\in {\Omega }_{t,c}^{g}$	Edges of the transport graph of transport system $t\in {T}^{g}$ for commodity c∈C
$\left({n}^{{\prime} },n\right),(n,n{\prime} )\in {\Omega }_{n,t,c}^{a}$	Edges the transport system $t\in {T}^{a}$ for the air distance point-to-point transport of commodity c∈C to or from node n∈N, which are defined based on the distance matrix ${D}_{n,n{\prime} }$ with ${D}_{n,n{\prime} }\le \hat{D}$
i∈I	Iteration index
b∈B	Set of branches
$(n,c)\in {\Omega }_{b}^{{NC}}$	Set of node-commodity tuples included in branch b∈B
Parameters	
${D}_{n,n{\prime} }$	Distance Matrix for modeling the point-to-point transport of new pipelines and roads
$\hat{D}$	Maximal distance
${U}_{{n}^{s},{n}^{s},{c}^{s}}$	Initial costs for producing the start commodity ${c}^{s}\in C$ at start node ${n}^{s}\in N$
${\vartheta }_{{c}^{{\prime} },c}^{{CC}}$	Conversion costs [€ MWh^−1^] for producing commodity c∈C from $c{\prime} \in C$
${\eta }_{{c}^{{\prime} },c}$	Conversion efficiency for modeling the material loss of a conversion process
${\eta }_{t}$	Transport efficiency for modeling the material loss of a transport process
${\vartheta }_{{n}^{{\prime} },n,c,t}^{{gTC}}$	Costs for the transport of commodity c∈C over an edge $({n}^{{\prime} },n)$ of the graph distance-based transport system $t\in {T}^{g}$
${\vartheta }_{{n}^{{\prime} },n,c,t}^{{aTC}}$	Costs for transporting commodity c∈C over an edge $({n}^{{\prime} },n)$ of the air distance-based transport system $t\in {T}^{a}$
${p}_{c}$	Commodity price of commodity c
Variables	
${u}_{{n}^{s},n,c}$	Cumulated conversion and transport costs for providing commodity c∈C at node n∈N starting from node ${n}^{s}$
${x}_{n,{c}^{{\prime} },c}$	Binary for modeling the existence of a conversion process at node n from $c{\prime}$ to c
${x}_{{n}^{{\prime} },n,c,t}$	Binary for modeling the existence of a transport route of commodity c from node ${n}^{{\prime} }$ to node n via the transport system t∈T

Equation (1) minimizes the total conversion and transport costs for multiple target commodities $c\in {C}_{{n}^{e}}^{e}$ at the optimal end node within a target region. Conversion and transport cost restrictions are modeled in Eqs. (2) - (4) based on Big-M inequalities. The conversion costs restriction of Eq. (2) states that in case of a conversion from commodity $c{\prime} \in C$ to c∈C at node n∈N (${x}_{n,{c}^{{\prime} },c}=1$), the current total costs is lower bounded by the previous total costs for delivering commodity $c{\prime} \in C$ to the current node, adjusted by the conversion efficiency, plus the costs for the actual conversion. In case of a transport of commodity c∈C from node $n{\prime} \in N$ to node n∈N (${x}_{{n}^{{\prime} },n,c,t}=1$) via existing pipelines or shipping routes, Eq. (3) ensures that the current total costs are lower bounded by the previous total cost for delivering commodity c∈C to the predecessor node $n{\prime}$, adjusted by the transport efficiency, plus the transport costs ${\vartheta }_{{n}^{{\prime} },n,c,t}^{{gTC}}$. Transport efficiencies are based on boil-off gases and the application of the commodity as marine fuel. If neither applies, the transport efficiency equals 1. Analogously, Eq. (4) demands that in case of a transport of commodity c∈C from node $n{\prime} \in N$ to node n∈N (${x}_{{n}^{{\prime} },n,c,t}=1$) based on an air distance point-to-point connection for modeling new pipelines and road transport, the current total costs are lower bounded by the previous total costs for delivering commodity c∈C to the predecessor node $n{\prime}$, plus the transport costs ${\vartheta }_{{n}^{{\prime} },n,c,t}^{{aTC}}$. For all nodes except of the start and end node, Eq. (5) ensures a nodal balance of conversion paths by demanding that the number of binaries for indicating incoming active transport and conversion options equal the number of outgoing binaries. To ensure that each node is visited only once on the route from the start to the end node, Eqs. (6) and (7) restrict the number of incoming and outgoing conversion and transport options to one, respectively. Finally, Eq. (8) defines the starting and Eq. (9) the ending condition that exactly one conversion or transport option is chosen at the start and end node. The methods to calculate transport and conversion costs are given in the Supplementary Methods 4 and 5.

In the All-Retrofit-Prices scenario, commodity prices ${{{\rm{p}}}}_{{{\rm{c}}}}$ are included in the calculation of the most cost-efficient solution. In this case, the objective function as in Eq. (10) is applied:

10 $$\min {\sum}_{{n}^{e}\in {N}^{e}}{\sum}_{c\in {C}_{{n}^{e}}^{e}}{u}_{{n}^{s},{n}^{e},c}-{p}_{c}$$

### Iterative processing of branches

Our approach is a multi-commodity multi-mean transport algorithm capable of finding the most cost-efficient transport route from pre-defined starting points to a desired location. If only one commodity is specified as the target commodity in the input parameter set, the algorithm will find the most cost-efficient route for that commodity. Specifying multiple eligible commodities and respective conversion processes allows the algorithm to select the route and the commodities to be transported. This reduces transport costs as the algorithm can take advantage of low transport costs for certain commodities. Finally, suppose the production costs of the different commodities at the starting location are specified. In that case, the algorithm will provide the total supply costs, considering production, transport, and conversion costs.

The algorithm generates possible solutions for transport from a starting point to the final destination through an iterative process. Decisions are continuously taken based on eligible conversions and routes, leading to a decision tree where each branch is created based on the choice of conversion or transport. Branches ${{\rm{b}}}$ include information on the current geographic position or node ${{\rm{n}}}$, the commodity ${{\rm{c}}},$ and the total costs ${{{\rm{u}}}}_{{{{\rm{n}}}}^{{{\rm{s}}}},{{\rm{n}}},{{\rm{c}}}}$ for delivering the commodity c to the current node. Furthermore, information on past nodes is also stored to avoid circulating between nodes. After each conversion or transport step, each branch is assessed to remove branches that violate set benchmarks. The overall objective of the approach is the minimization of the total costs at the final destination $\hat{{{\rm{n}}}}$ for all branches whose commodity is one of the target commodities in ${{\rm{c}}}\in {{{\rm{C}}}}_{{{{\rm{n}}}}^{{{\rm{e}}}}}^{{{\rm{e}}}}$. More information on the branching procedure can be found in Supplementary Method 1.

### Computational methods

All computational analyses and visualizations were performed using the custom HERMES software (Hydrogen Economy Routing Model for Cost-Efficient Supply), version 1.0.0, implemented in Python 3.11. HERMES was used to preprocess spatial infrastructure data, generate transport networks, calculate shortest paths, identify cost-efficient multimodal transport and conversion routes, aggregate supply potentials, and produce the reported figures. The software environment included NumPy v1.26.4, pandas v2.2.0, SciPy v1.15.3, Joblib v1.3.2, NetworkX v3.2.1, PyTables v3.9.2, openpyxl v3.1.5, Shapely v2.0.3, GeoPandas v0.14.3, Fiona v1.9.6, PyProj v3.7.1, Rtree v1.4.0, GeoPy v2.4.1, geovoronoi v0.4.0, GeoJSON v3.1.0, vincenty v0.1.4, SeaRoute v1.3.1, Matplotlib v3.8.3, Seaborn v0.13.2, Plotly v6.7.0, CairoSVG v2.7.1, tqdm v4.66.2, psutil v5.9–7.x, PyYAML v6.0, gurobipy v12.0.3, and Gurobi Optimizer v12.0.3. OpenAI Codex and ChatGPT were used to support code development and language editing, as disclosed in the Acknowledgments. Geographical boundaries and base-map geometries used in the reported figures were obtained from Natural Earth^39^. The complete dependency specification is provided with the archived source code. The main calculations were conducted on Ubuntu 20.04.6 LTS.

### Reporting summary

Further information on research design is available in the Nature Portfolio Reporting Summary linked to this article.

## Supplementary information

Supplementary Information

Description of Additional Supplementary Files

Supplementary Data 1

Reporting Summary

Transparent Peer Review file

## Source data

Source Data

## Data Availability

The data generated and used in this study have been deposited in Zenodo^40^. The data underlying all manuscript figures are provided in the accompanying Source Data file. Additional figures, notes, tables, methods, and data required for the analyses are provided in the Supplementary Information and Supplementary Data files. No private or restricted-access data were used in this study. Source data are provided with this paper.

## Code availability

The model HERMES – **H**ydrogen **E**conomy **R**outing **M**odel for cost-**e**fficient **S**upply^41^ is implemented in Python and available here: https://github.com/ulicious/hermes. Version 1.0.0, used for calculating the results in this publication, has been archived^42^. This repository also contains the configuration files used to calculate the results, as well as all processed data, starting locations, and results for the minimal example. Further documentation can be found here: https://hermes-h2.readthedocs.io/en/main/
