# Supplementary material for: Mapping Future Energy Journeys Through Modeling the Global Transport of Synthetic Energy Carriers
Source: Nat Commun. 2026 Nov 7;17:11500. doi: 10.1038/s41467-026-78710-7 (PMC13743743; doi:10.1038/s41467-026-78710-7)
Supplement: Supplementary file 1 [file 41467_2026_78710_MOESM1_ESM.pdf]

Mapping Future Energy Journeys Through Modeling the Global Transport of Synthetic Energy Carriers

SUPPLEMENTARY INFORMATION

Uwe Langenmayr[1,*], Julia Schuler[1], Viktor Slednev[1] & Wolf Fichtner[1]

[1] Institute for Industrial Production (IIP), Karlsruhe Institute of Technology (KIT), Karlsruhe, Germany

* Corresponding author: uwe.langenmayr@kit.edu

Contents

Supplementary Figures 2

Supplementary Note 1 13

Supplementary Note 2 15

Supplementary Note 3 20

Supplementary Note 4 22

Supplementary Note 5 23

Supplementary Method 1 24

Supplementary Method 2 27

Supplementary Method 3 31

Supplementary Method 4 34

Supplementary Method 5 38

Supplementary Method 6 43

Supplementary References 44

Supplementary Figures

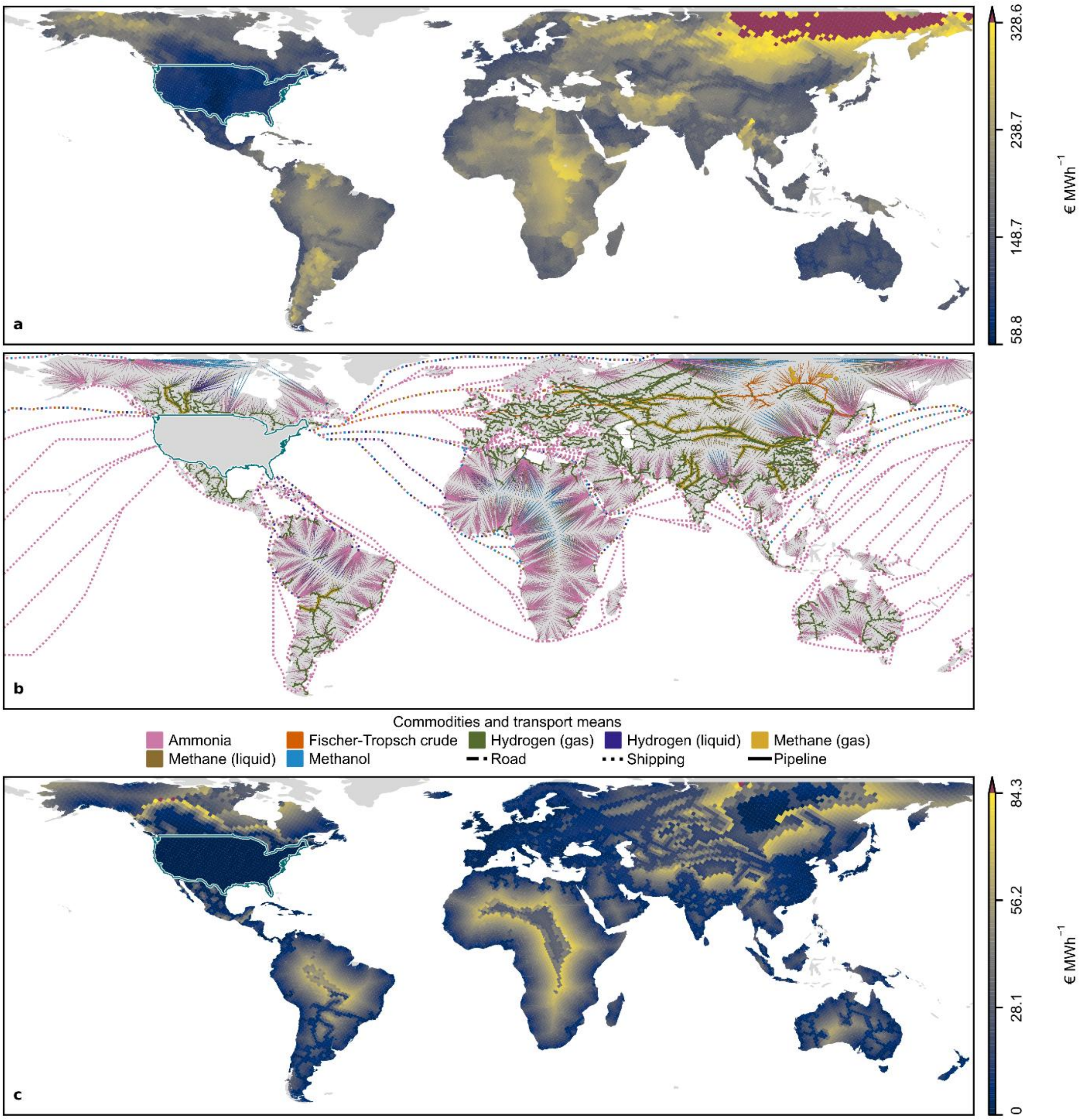

Supplementary Fig. 1. Breakdown of supply costs, logistics routes, and transport economics for the All-Retrofit scenario with the destination USA (without Hawaii, Alaska, and other overseas territories) under the All-Retrofit scenario. (a) Total supply costs. (b) Routes and transported synthetic energy carriers. (c) Transport costs.

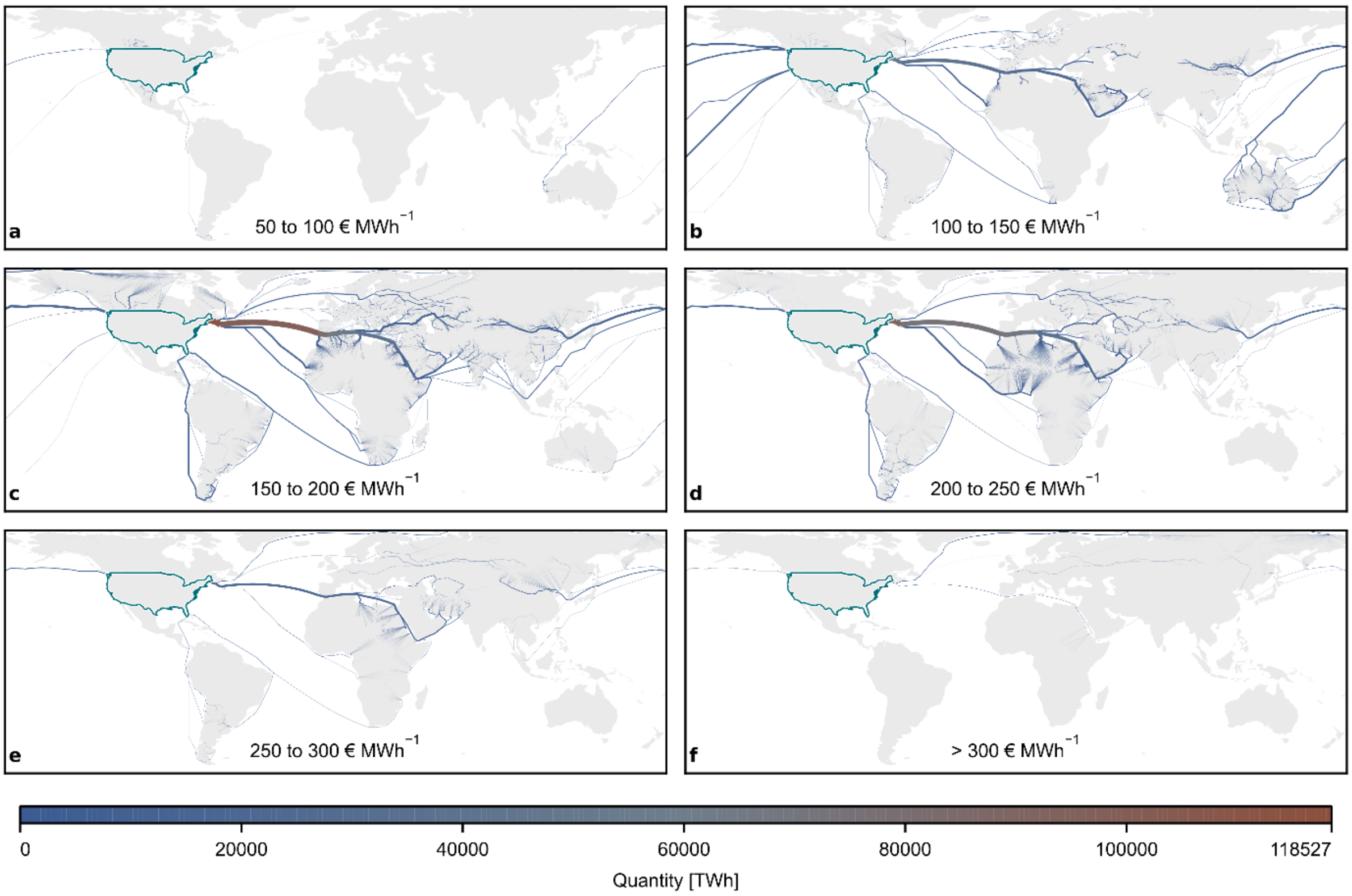

Supplementary Fig. 2. Quantity-weighted transport routes to the USA in the All-Retrofit scenario, categorized by total supply costs. (a) 50 to 100 € per MWh. (b) 100 to 150 € per MWh. (c) 150 to 200 € per MWh. (d) 200 to 250 € per MWh. (e) 250 to 300 € per MWh. (f) More than 300 € per MWh.

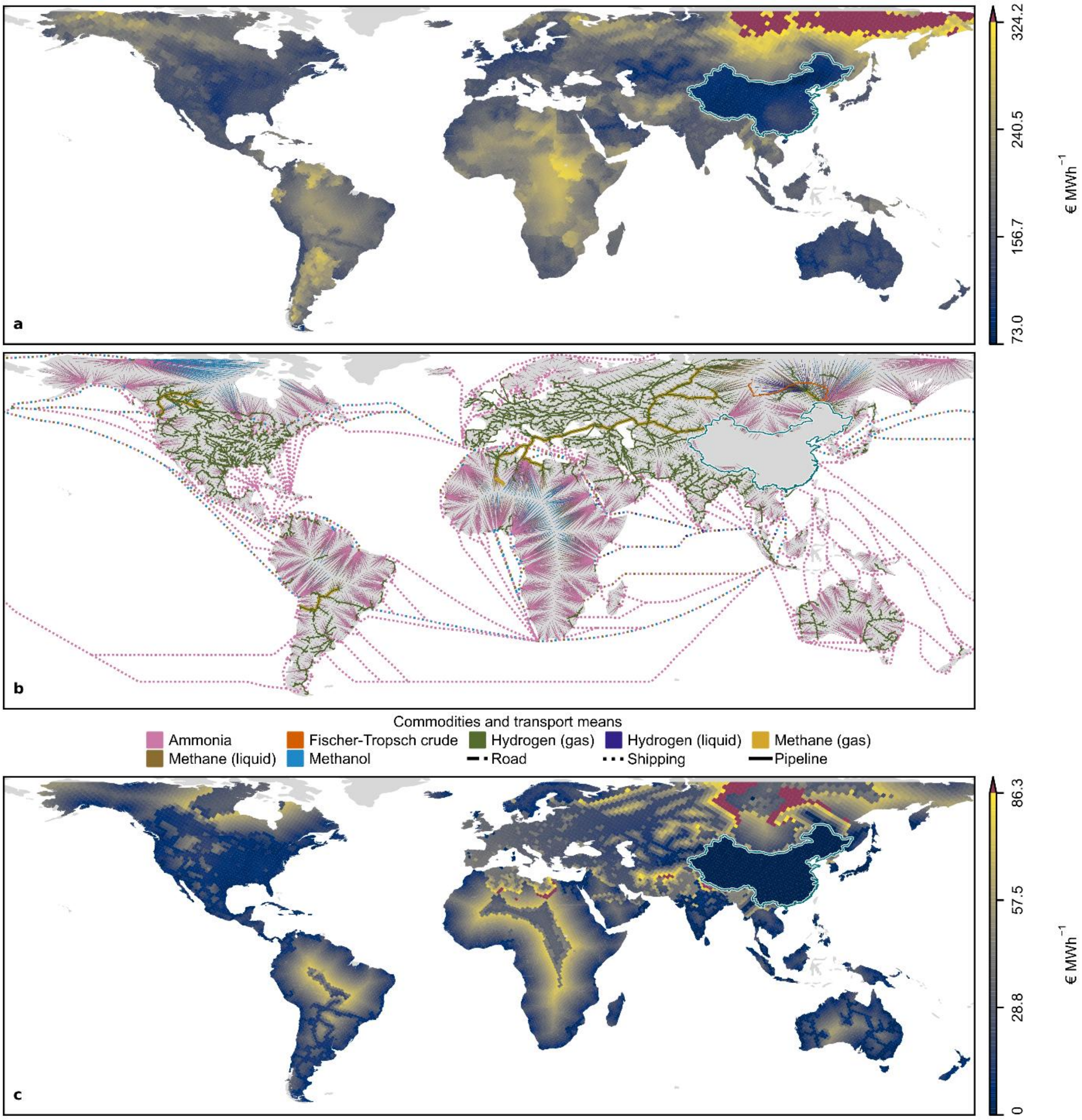

Supplementary Fig. 3. Breakdown of supply costs, logistics routes, and transport economics for the All-Retrofit scenario with the destination China. (a) Total supply costs. (b) Routes and transported synthetic energy carriers. (c) Transport costs.

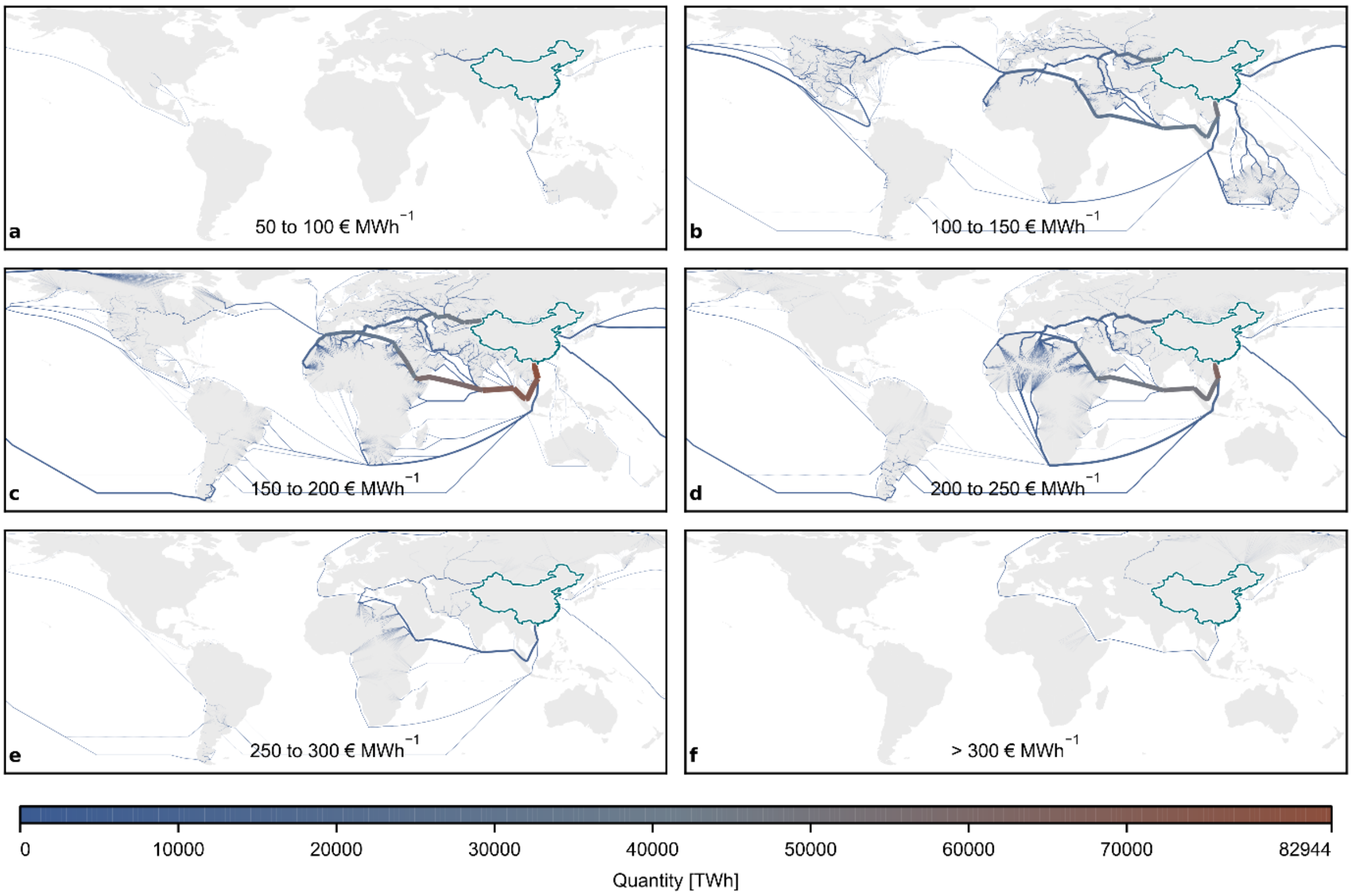

Supplementary Fig. 4. Quantity-weighted transport routes to China in the All-Retrofit scenario, categorized by total supply costs. (a) 50 to 100 € per MWh. (b) 100 to 150 € per MWh. (c) 150 to 200 € per MWh. (d) 200 to 250 € per MWh. (e) 250 to 300 € per MWh. (f) More than 300 € per MWh.

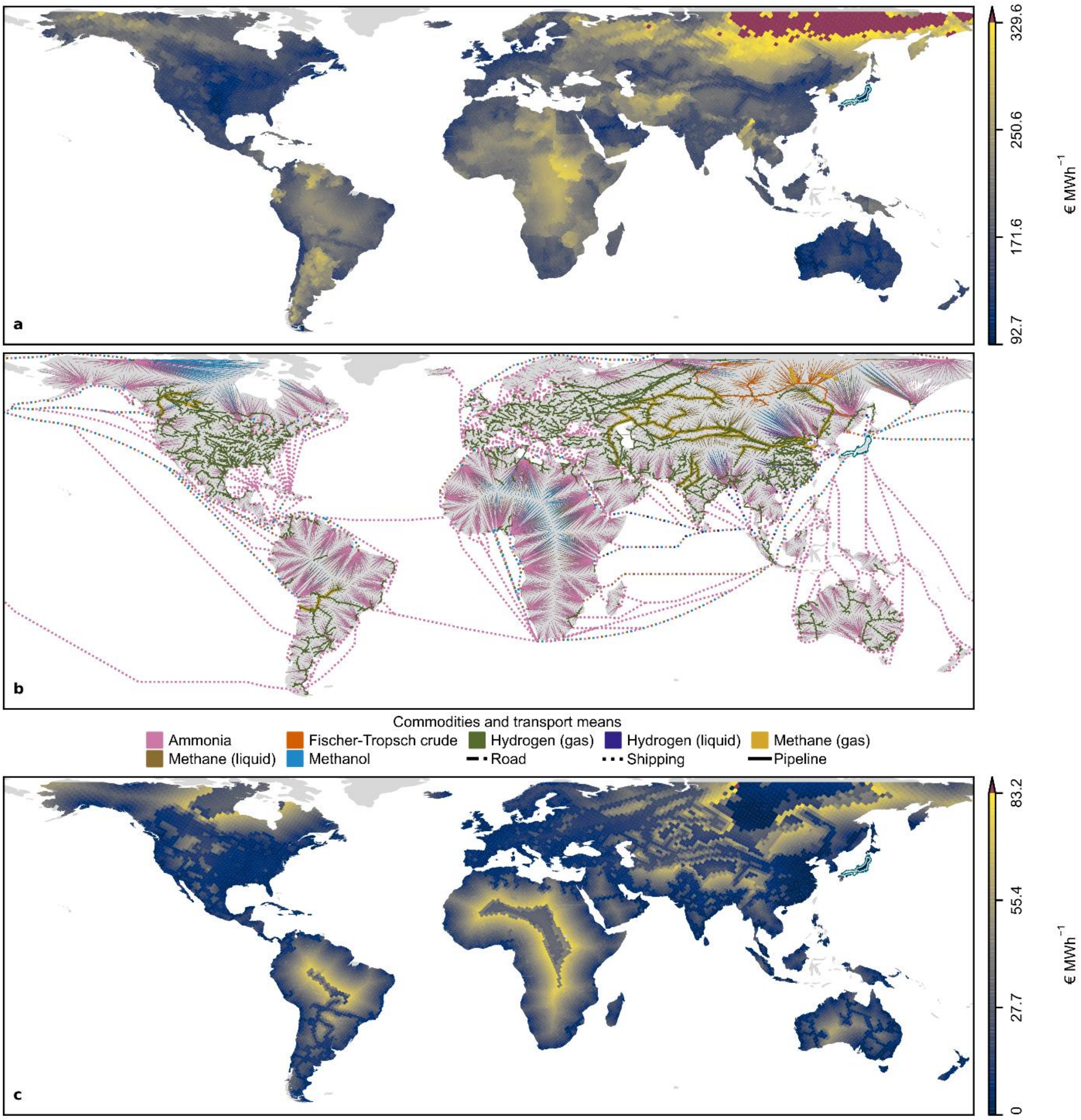

Supplementary Fig. 5. Breakdown of supply costs, logistics routes, and transport economics for the All-Retrofit scenario with the destination Japan. (a) Total supply costs. (b) Routes and transported synthetic energy carriers. (c) Transport costs.

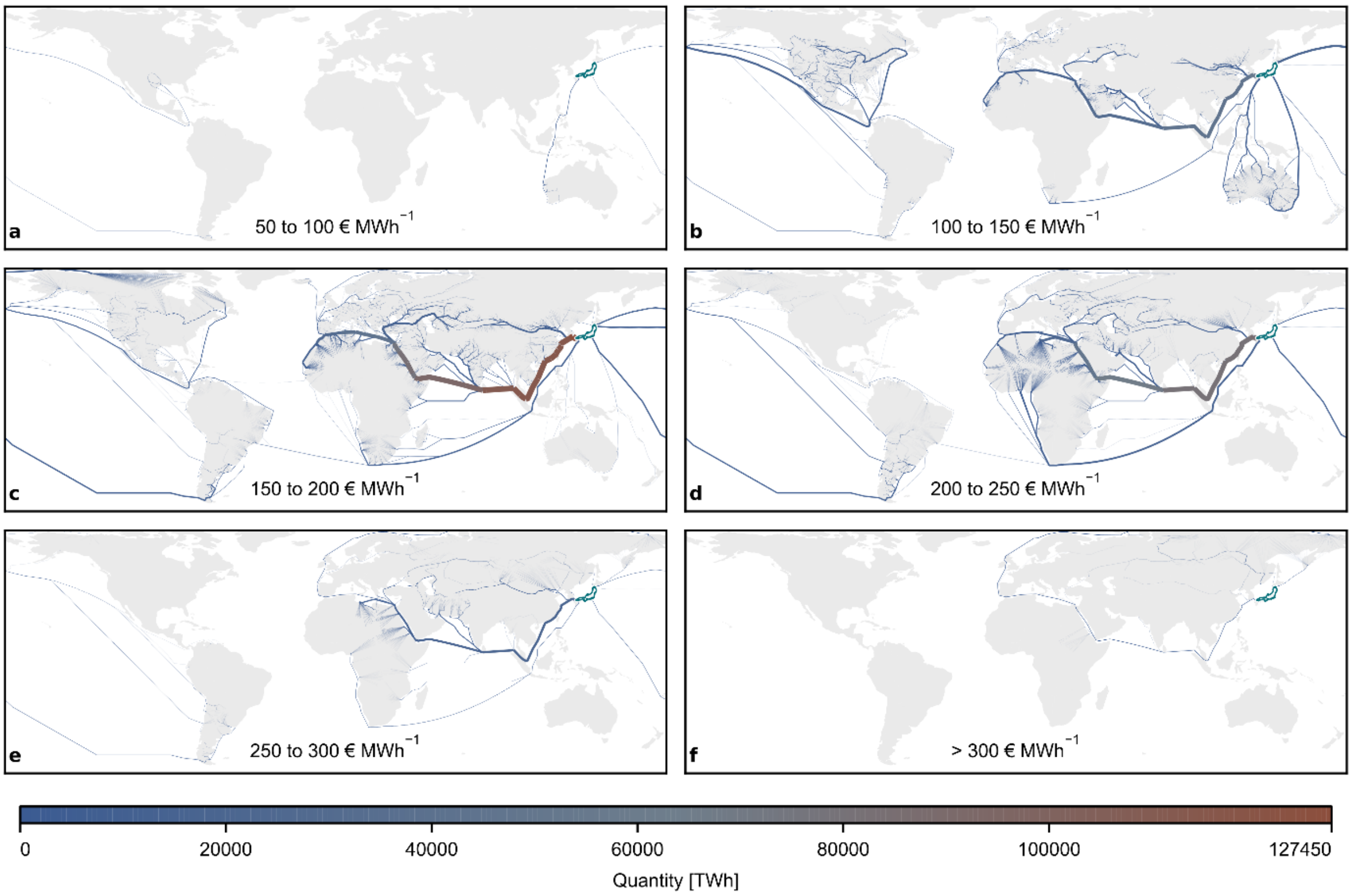

Supplementary Fig. 6. Quantity-weighted transport routes to Japan in the All-Retrofit scenario, categorized by total supply costs. (a) 50 to 100 € per MWh. (b) 100 to 150 € per MWh. (c) 150 to 200 € per MWh. (d) 200 to 250 € per MWh. (e) 250 to 300 € per MWh. (f) More than 300 € per MWh.

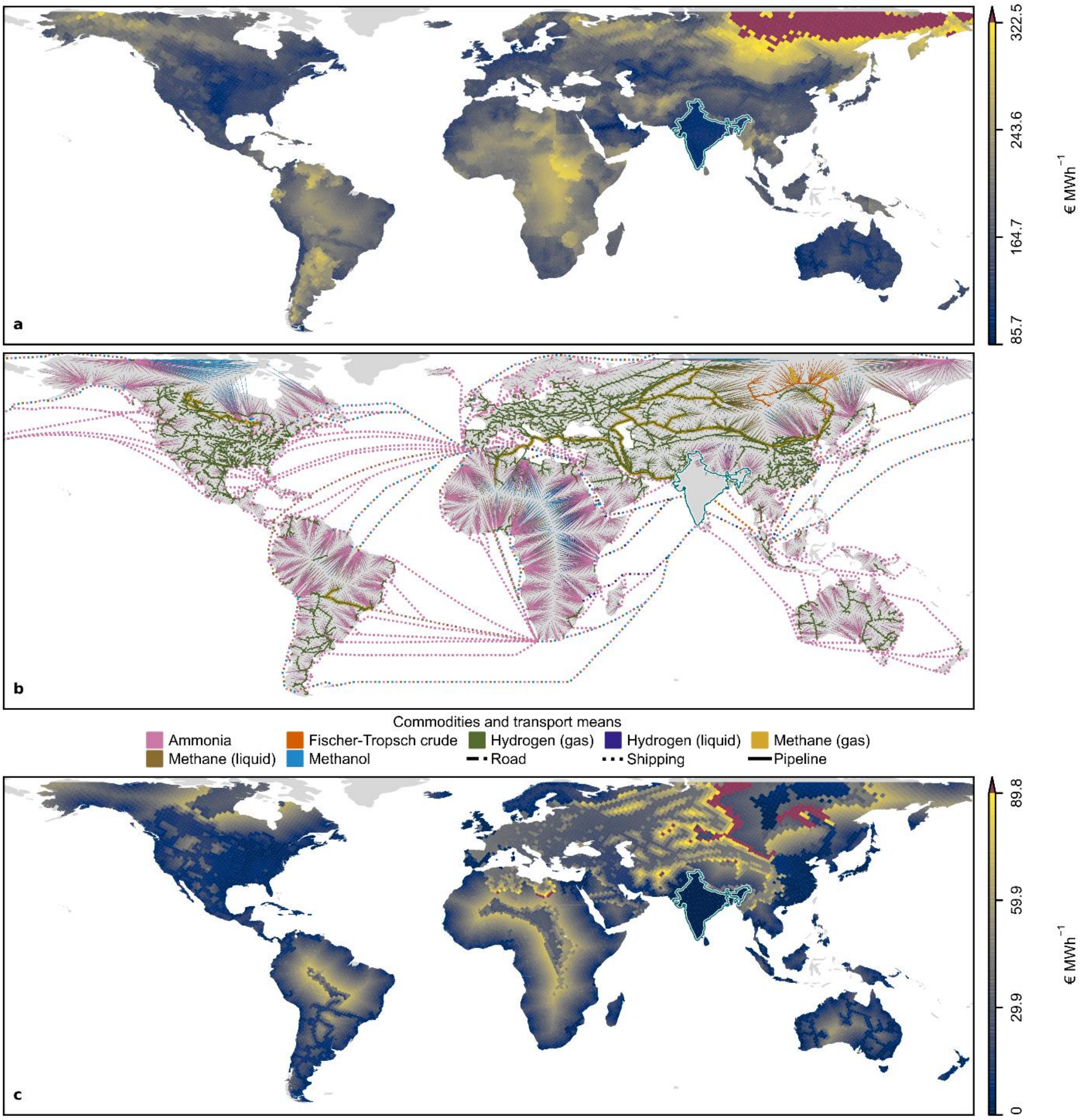

Supplementary Fig. 7. Breakdown of supply costs, logistics routes, and transport economics for the All-Retrofit scenario with the destination India. (a) Total supply costs. (b) Routes and transported synthetic energy carriers. (c) Transport costs.

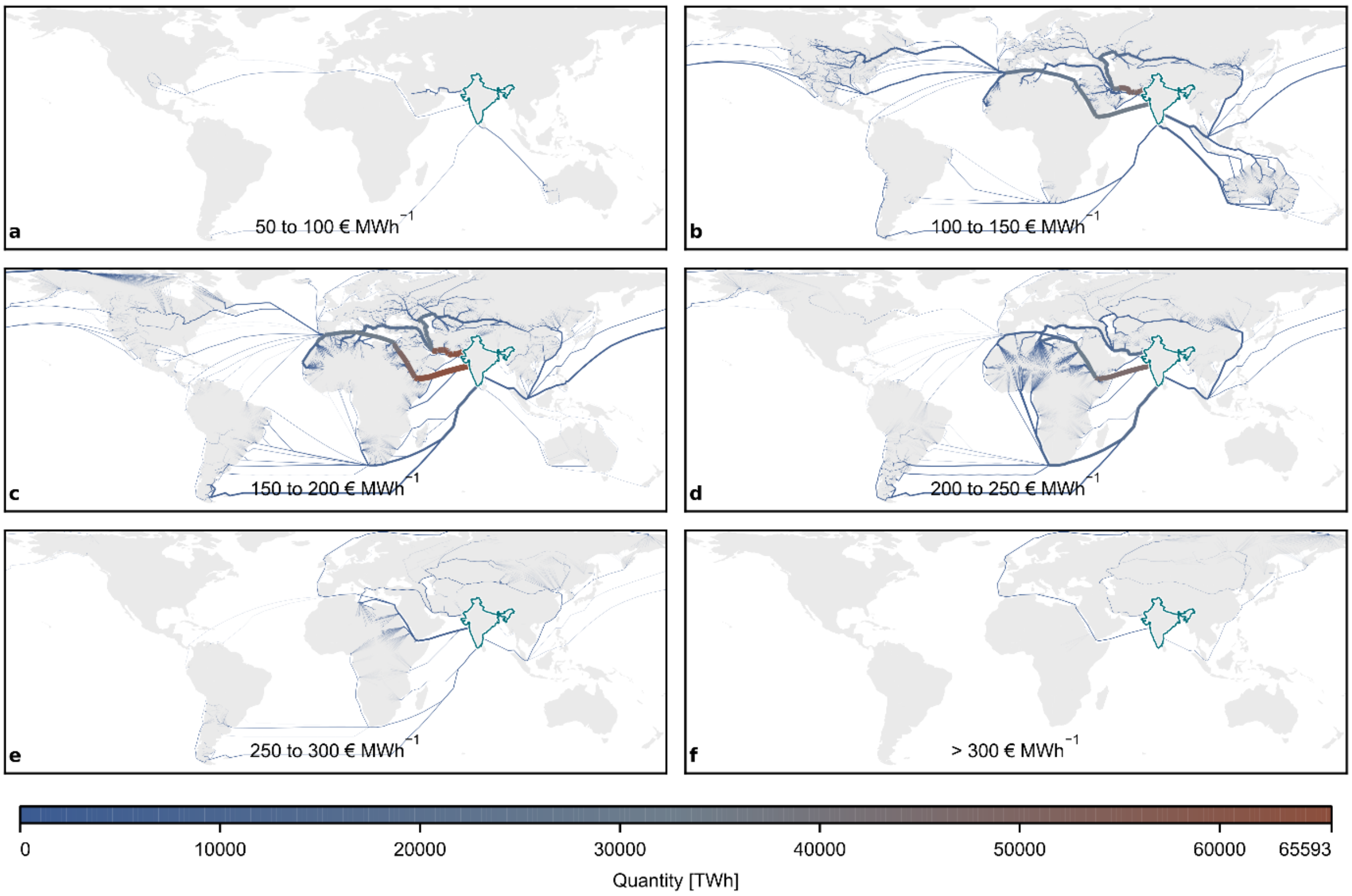

Supplementary Fig. 8. Quantity-weighted transport routes to India in the All-Retrofit scenario, categorized by total supply costs. (a) 50 to 100 € per MWh. (b) 100 to 150 € per MWh. (c) 150 to 200 € per MWh. (d) 200 to 250 € per MWh. (e) 250 to 300 € per MWh. (f) More than 300 € per MWh.

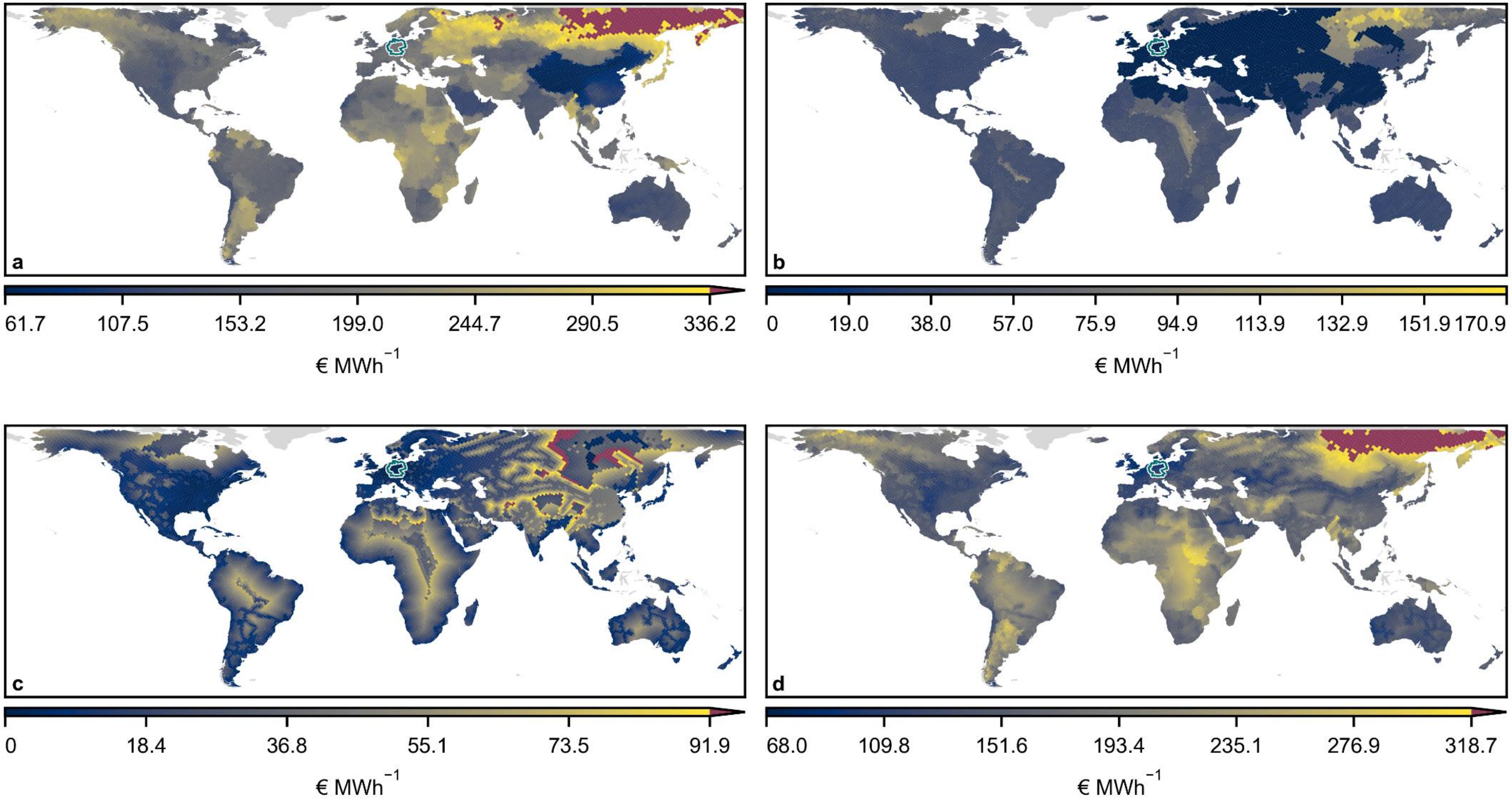

Supplementary Fig. 9. Breakdown of different cost components of the GER-All-Retrofit scenario. (a) Hydrogen production costs. (b) Conversion costs. (c) Transport costs. (d) Total supply costs.

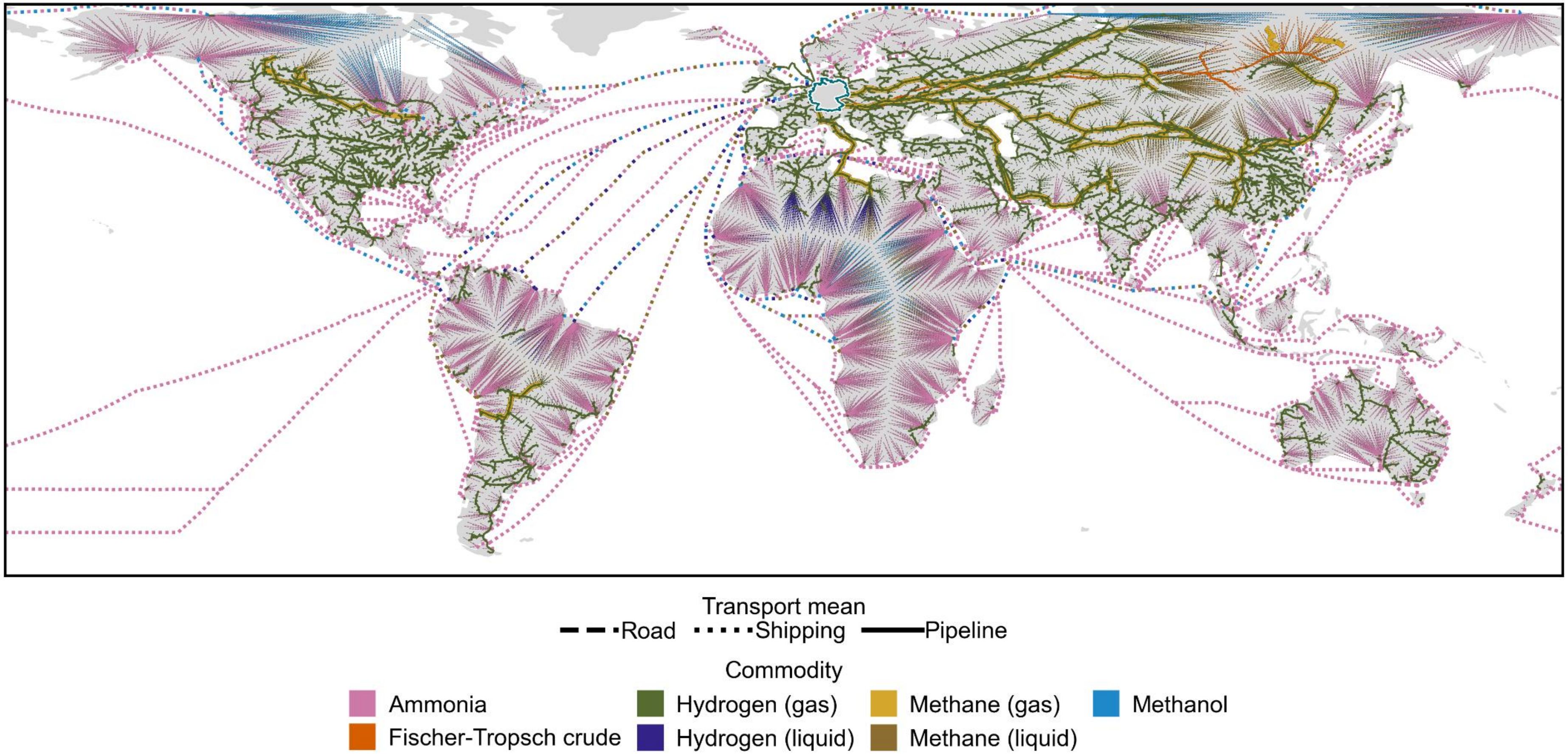

Supplementary Fig. 10. Transport means and transported commodities for all export locations in the GER-All-Retrofit scenario

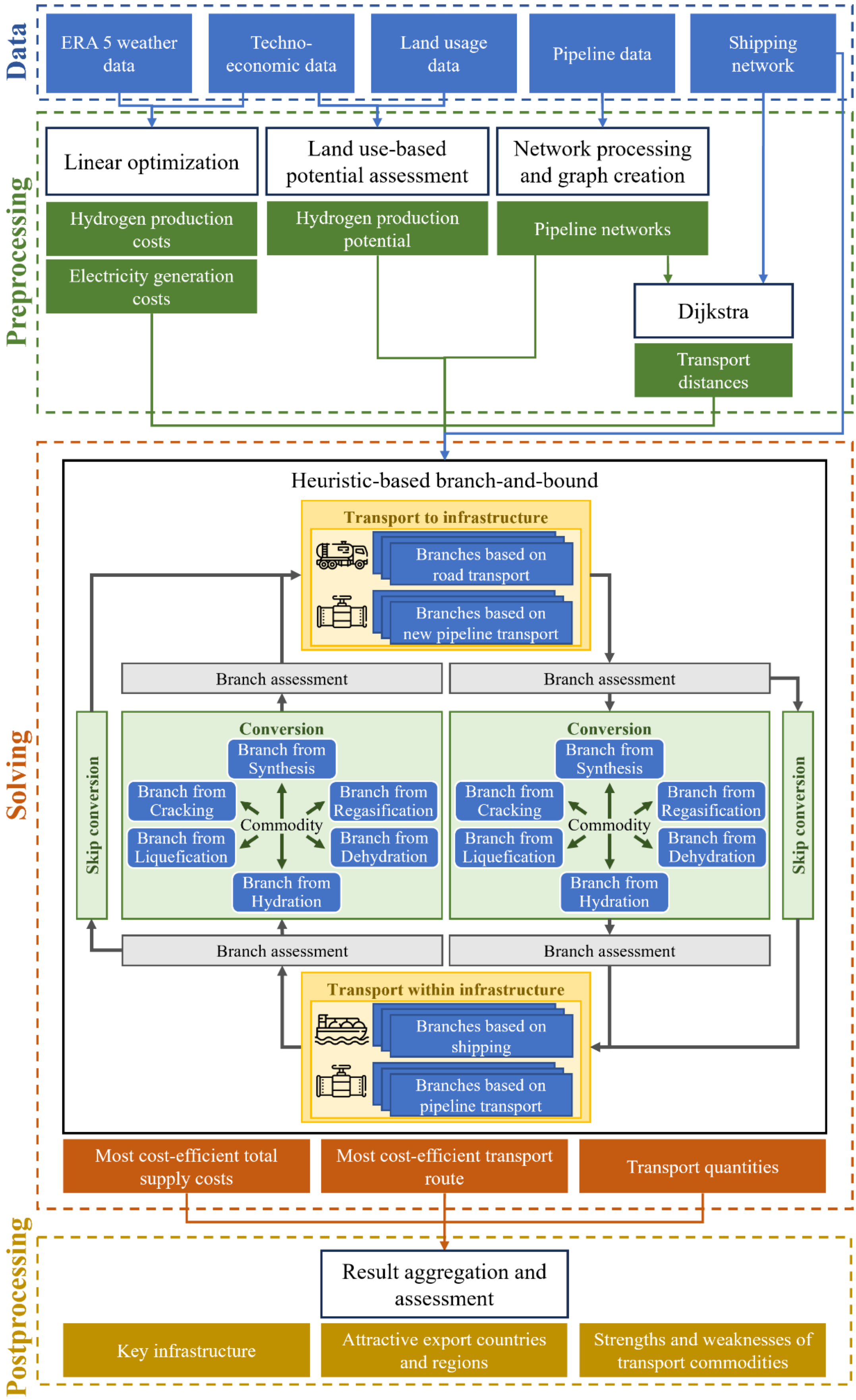

Supplementary Fig. 11. Overview of the modeling methodology[1–4]

Supplementary Note 1

Efficiency of transport routes in the *All-Retrofit* scenario

Transport efficiency of the different routes based on losses of transported commodities. Losses are only calculated as losses of the transported commodity. Energy demands of applications used for transport, which do not consume the transported commodity itself, are not considered (e.g., gas compressor stations, truck fuel, etc.)

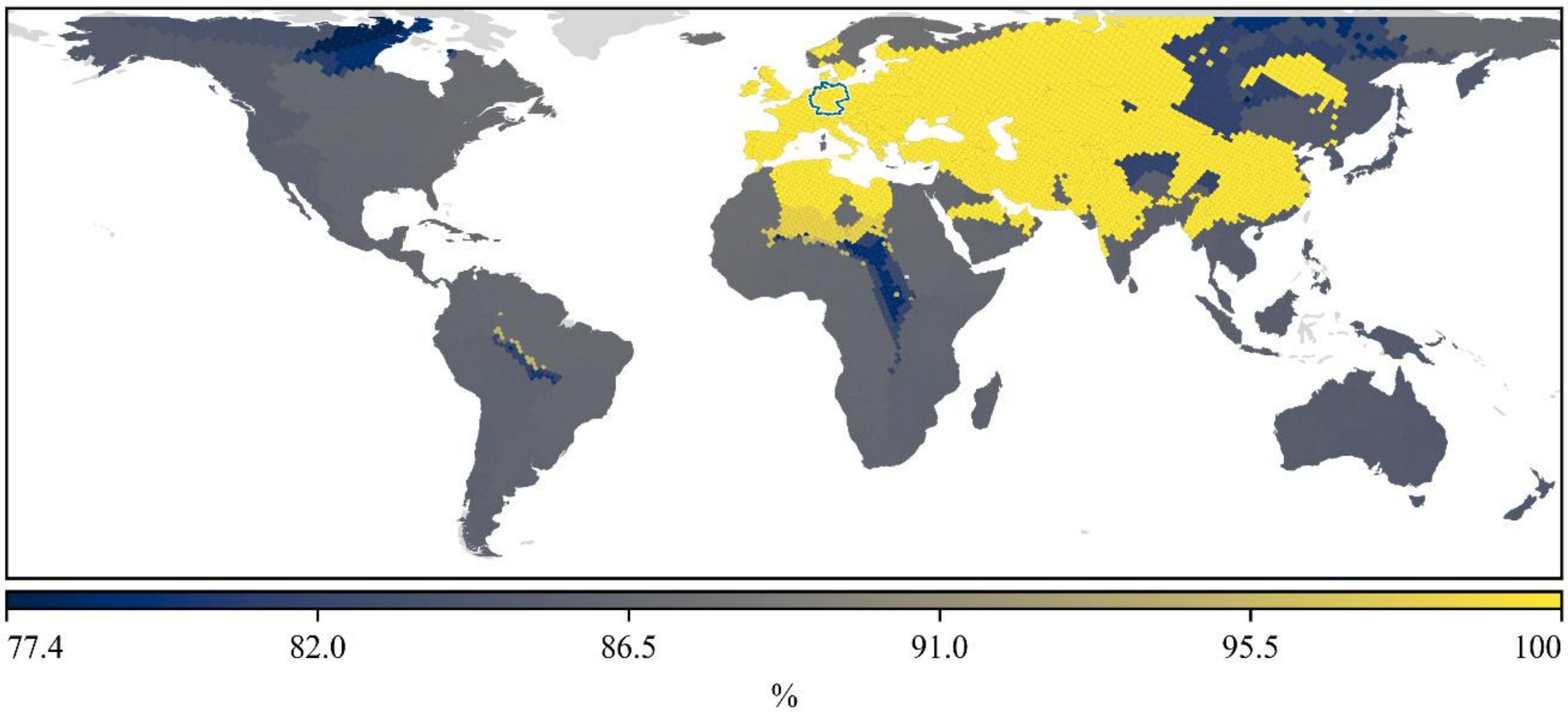

Supplementary Fig. 12. Efficiencies of the supply chains based on conversion and transport losses in the GER-All-Retrofit scenario

The chart below shows the most common combinations of commodities and modes of transport, their frequencies, and the average losses at each stage of the value chain.

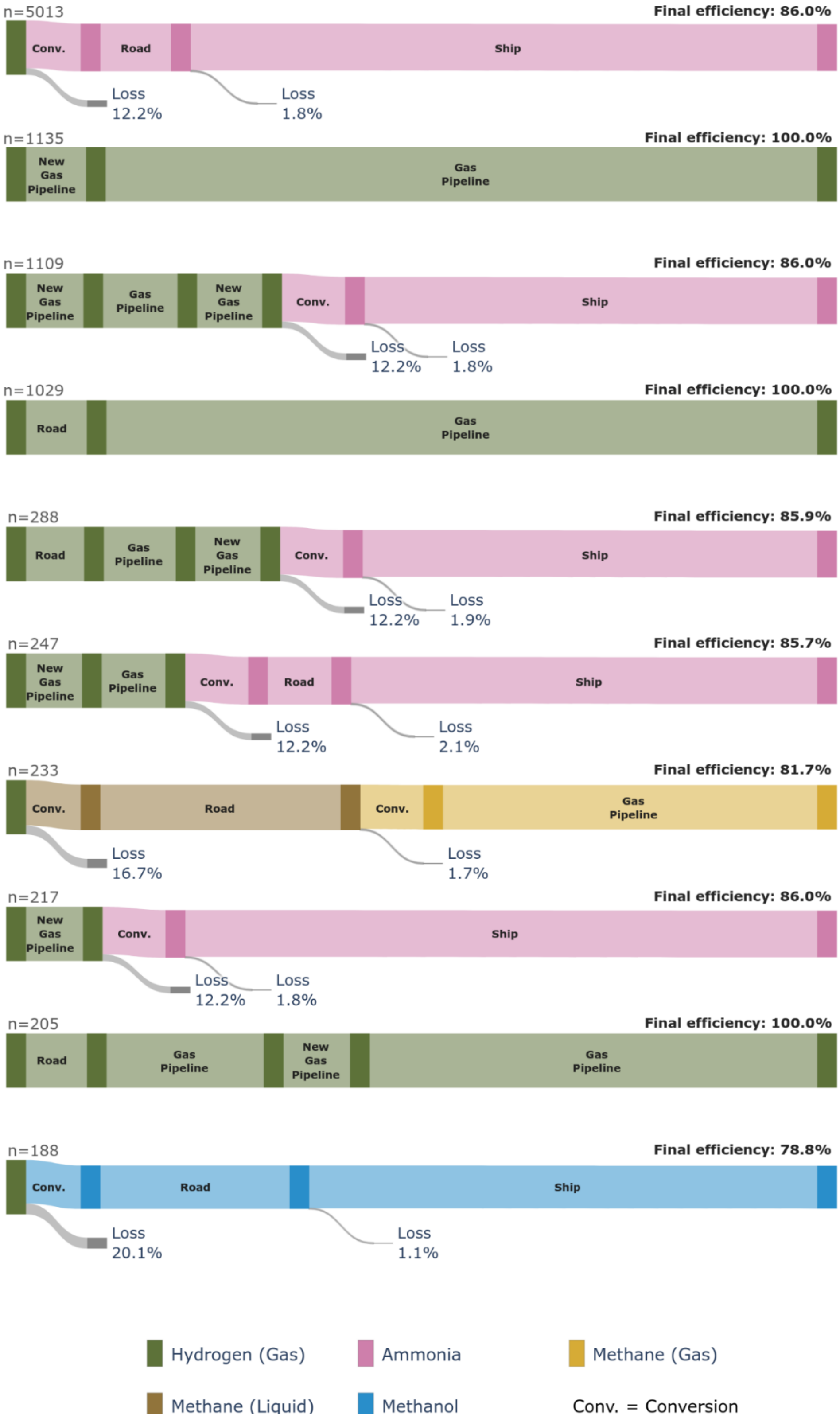

Supplementary Fig. 13. Efficiency losses along the supply chain for the 10 most common routes based on their sequence of conversions and transport. Losses are limited to those of the transported commodity along the supply chain. Hydrogen production efficiency or processes in which the commodity is not used to provide energy (e.g., compression during pipeline transport) are not considered. n shows the number of routes using the same sequence. Losses are averaged across all n routes using the same sequence.

Supplementary Note 2

Considering water availability in the All-Retrofit scenario

We extend the All-Retrofit scenario to account for local water availability at the export locations. To exclude locations with insufficient water availability, we applied two filters:

- Exclude all regions that have a *Baseline Water Stress* indicator above 3, corresponding to the High and Extremely High stress categories defined by World Resource Institute (2025)[5], because these regions already experience severe competition for renewable freshwater resources between municipalities, agriculture, and industry, making additional large-scale electrolysis demand particularly critical. The data is available in World Resource Institute (2026)[6].
- Areas in 50 km proximity to shores and coastlines are included, even when the Baseline Water Stress indicator is above 3, since the option to use saltwater desalination is assumed to be available.

The available and excluded areas are shown in *Supplementary Fig. 14*, and the following figures show the total supply costs, taken routes and transport costs (*Supplementary Fig. 15*), the comparison of the Algerian and Australian supply curves with and without the consideration of the water availability (*Supplementary Fig. 16* and *Supplementary Fig. 17*), and the most used transport routes when considering water availability (*Supplementary Fig. 18*).

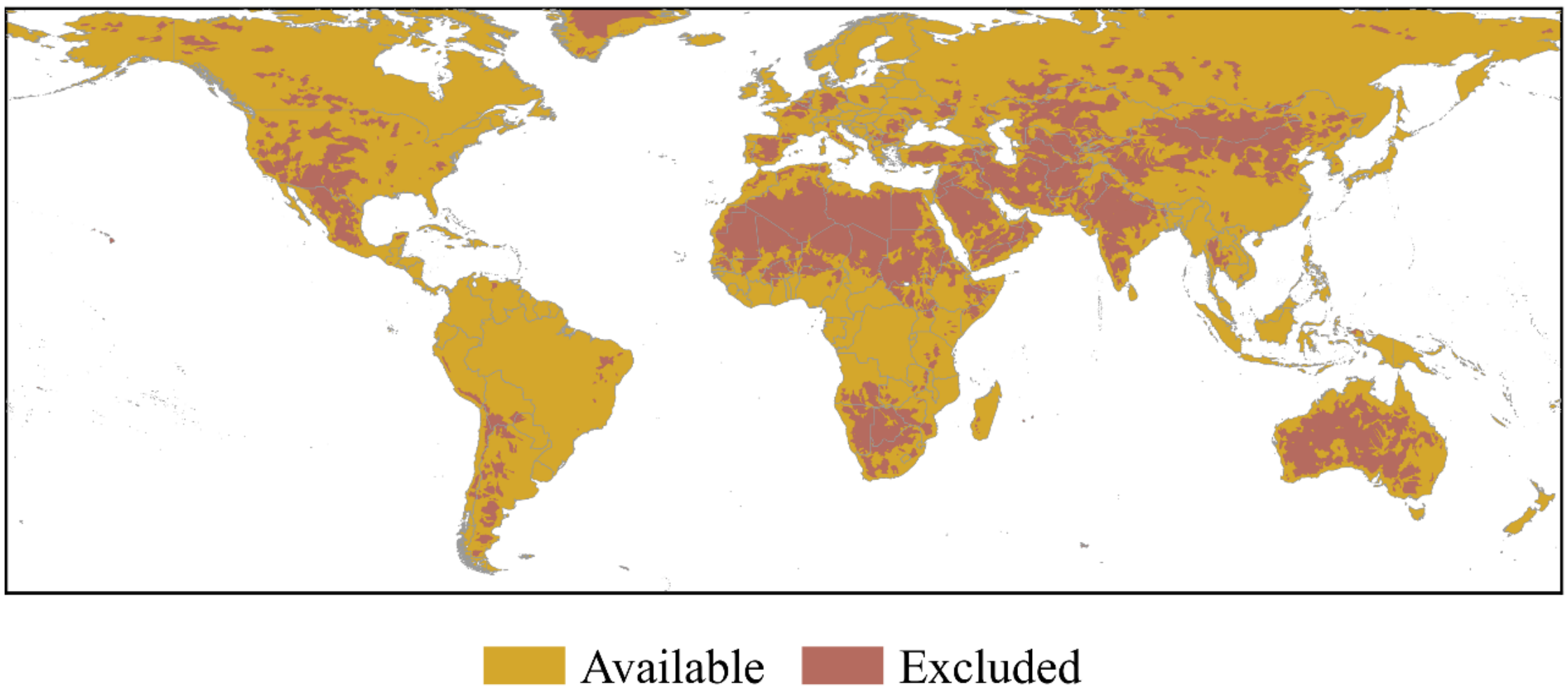

Supplementary Fig. 14. Exclusion of regions in scenarios accounting for local water availability. Areas considered available are shown in yellow, and excluded areas are shown in red, based on water-stress levels and a maximum distance to shore of 50 km

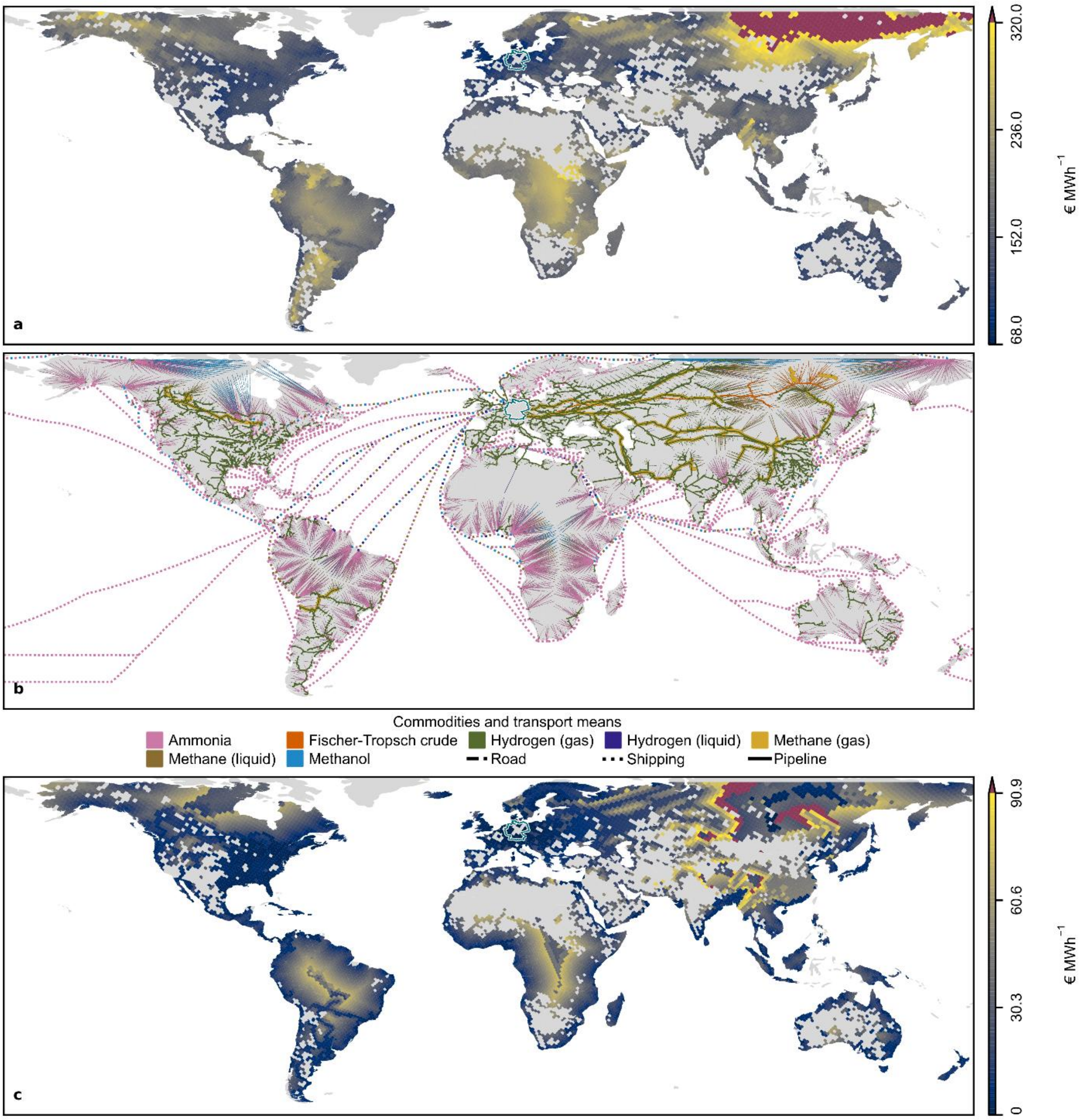

Supplementary Fig. 15. Breakdown of supply costs, logistics routes, and transport economics for the All-Retrofit scenario with the destination Germany and the exclusion of regions with insufficient water supply. (a) Total supply costs. (b) Routes and transported synthetic energy carriers. (c) Transport costs.

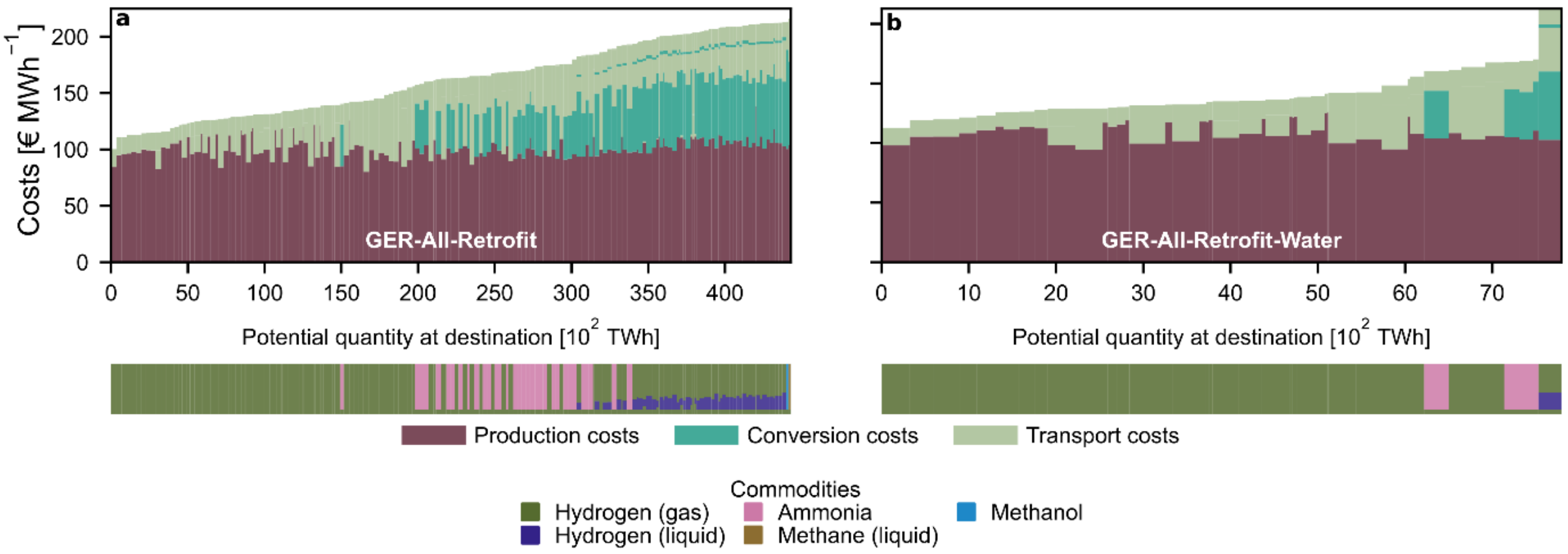

Supplementary Fig. 16. Supply curves and commodity bands for SEC transport from Algeria to Germany. (a), Scenario without consideration of water availability. (b), Scenario with consideration of water availability

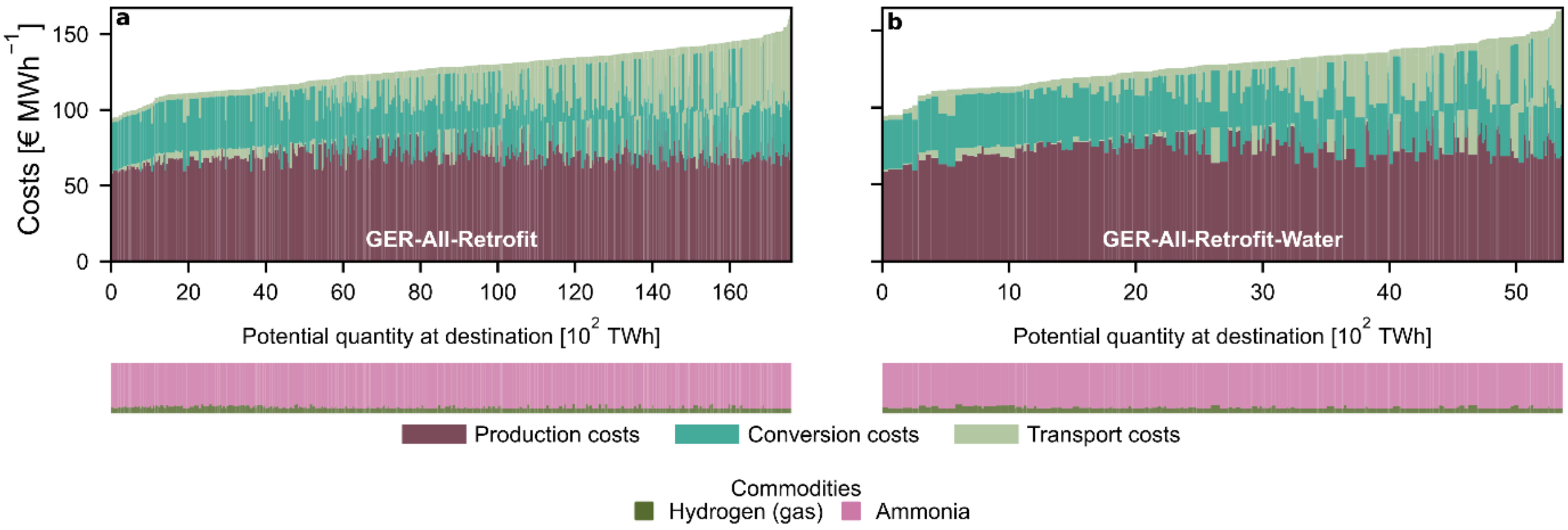

Supplementary Fig. 17. Supply curves and commodity bands for SEC transport from Australia to Germany. (a), Scenario without consideration of water availability. (b), Scenario with consideration of water availability

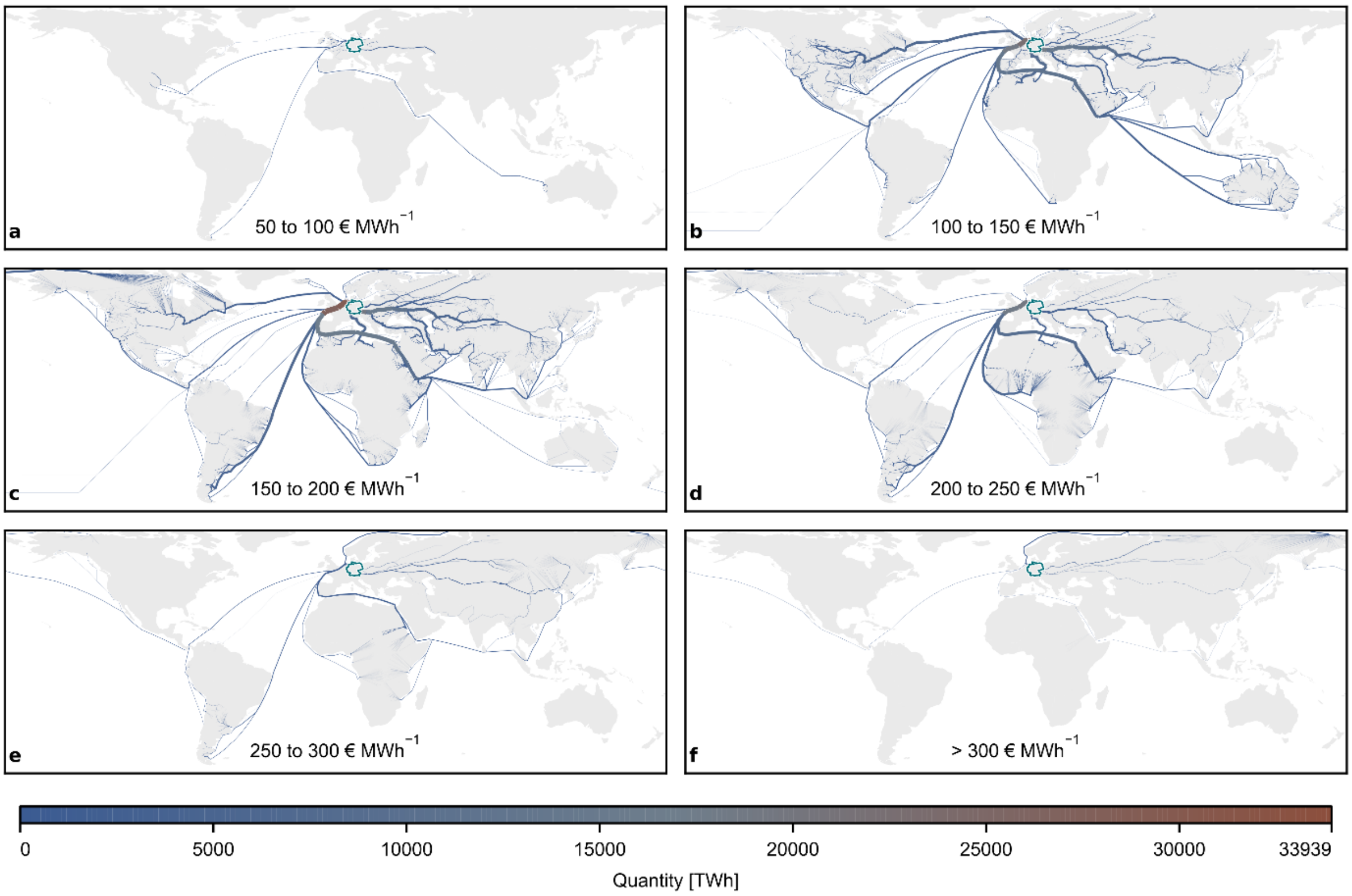

Supplementary Fig. 18. Quantity-weighted transport routes to Germany in the All-Retrofit-Water scenario, categorized by total supply costs. (a) 50 to 100 € per MWh. (b) 100 to 150 € per MWh. (c) 150 to 200 € per MWh. (d) 200 to 250 € per MWh. (e) 250 to 300 € per MWh. (f) More than 300 € per MWh.

Supplementary Note 3

Assessment of the optimality of the calculated solutions

The main disadvantage of the heuristic is its inability to prove the optimality of the most cost-efficient solutions. To address this challenge, we implemented a mixed-integer optimization model and applied it to the All-Retrofit case study, but adjusted the geographical scope to reduce complexity. The case study covered Belgium and Luxembourg as the starting region, split into 29 cells, and the Netherlands as the destination country. Infrastructure, such as ports and pipelines, was considered within the geographical scope of 49°-54° latitude and 2°-8° longitude. We applied the same settings as in the All-Retrofit scenario: all commodities, except the LOHCs, are target commodities, and the pipeline infrastructure can be retrofitted to transport hydrogen. This setting results in a model having between 509,622 and 509,790 edges.

Before solving the mixed-integer problem, we used the heuristic to obtain the most cost-efficient solutions, which served as a warm start for the optimization. The heuristic took 45 seconds to solve for all locations (see *Supplementary Note 4* for the solving environment). In the optimization model, we applied three approaches. First, we ran the optimization problem without a warm start; then, with a warm start based on the heuristic solution; and finally, using the total costs from the warm start to prune the decision tree by removing all edges that would result in total costs higher than the total supply costs of the warm start solution. In addition, we use the total supply costs of the warm start to define a tight BigM.

Using the first approach, the problem didn't find a feasible solution when running for the full 60 minutes. In the second case, the mixed-integer problem accepted the warm-start solution as a feasible solution and began proving optimality. The objective function did not improve within 60 minutes, but neither could the optimality be proved. In the final approach, we used pruning to remove up to 509,732 edges, leaving some models with only 25 edges (in the smallest model). This approach significantly improved the best bound of the MIP problem, leading to a substantially reduced MIP gap and potentially proving optimality within a few seconds using the default MIP gap.

In the next step, we tested the different configurations. In general, the approach showed a similar behavior when gaseous hydrogen is set as the target commodity. However, as soon as other commodities were set

as the target commodity, the MIP's performance decreased. Especially since the warm-start solution's total supply costs were higher, reducing the efficiency of pruning the decision tree before entering the MIP. For instance, when choosing Ammonia as the target commodity, the number of removed edges ranged from 376,738 to 379,234.

We tried to scale up the model to a larger geographical scope. Here, we used Morocco and Algeria as export regions (29 start locations) and Spain as the destination. This model resulted in a range of 1,755,292 to 1,755,340 edges. By applying the warm-start solution, we reduced the number of edges by a range of 499,978 to 1,711,437. Gurobi's presolve further reduced the number of edges, sometimes even resulting in the optimal solution. From the 29 locations, only 11 were solved and optimality was proven. In these cases, the filter and the presolve reduced the number of edges to below 10,000. None of the others found a better solution than the warm-start solution within the one-hour time limit.

The number of edges is the main challenge in both cases, the heuristic and the MIP. However, the heuristic only considers feasible edges in each iteration, whereas the MIP considers all edges simultaneously. This is already an advantage of the heuristic, since the full model does not need to be built. The second advantage is the ability to prune edges while solving the model. With each update of the total supply costs, created branches can be pruned and potential routes excluded.

Altogether, this shows the advantage of the developed heuristic. Even for small data excerpts, the MIP does not solve without a warm-start solution, whereas the heuristic solves the problem reliably. Even with the warm-start solution, the MIP already takes longer to build the problem than the heuristic does to solve it, due to the large number of variables and constraints. We further showed that the MIP does not provide better solutions than our heuristic for the Benelux case and the Northwest Africa-to-Spain case. This was demonstrated by either running the MIP until the optimum was achieved or by terminating the optimization after 60 minutes. In both cases, the MIP resulted in the same objective function and route as the heuristic.

Supplementary Note 4

Description of computation infrastructure and computation time

The heuristic is implemented to achieve high computational efficiency without terminating the most cost-efficient solution. The processor is an AMD Ryzen Threadripper 3990X 64-Core at 2.9 GHz, allowing us to utilize up to 128 CPUs. The system has 256 GB of RAM. The installed operating system is Ubuntu 20.04.6 LTS. This system achieved computation times per export location per scenario, as shown in *Supplementary Fig. 19*.

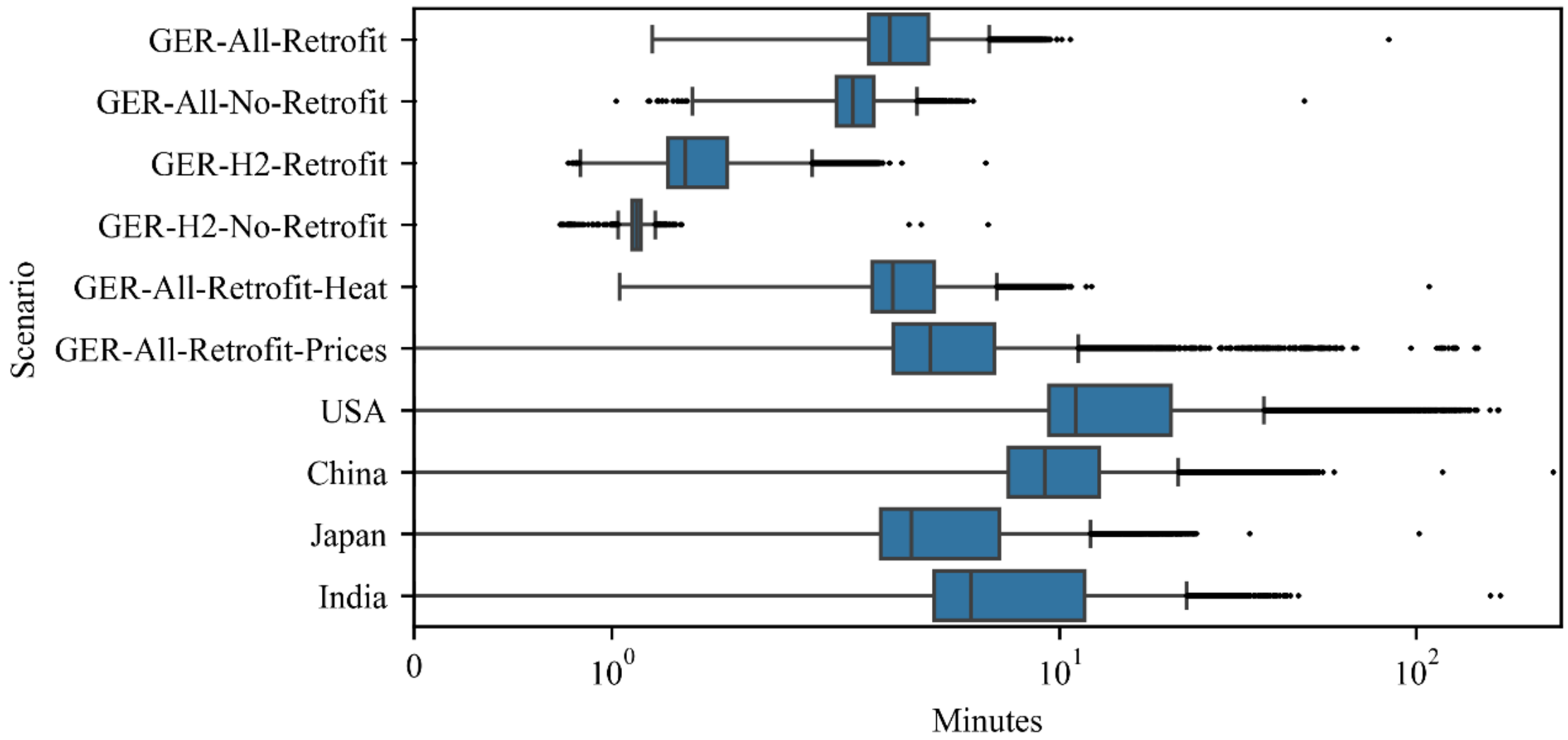

Supplementary Fig. 19. Calculation times per scenario

To solve the MIP, we used Gurobi 12.0.3.[7]

Supplementary Note 5

Global transport infrastructure

All underlying infrastructure data is open data. The *Global Energy Monitor* supplies global oil pipeline[8] and gas pipeline[9] infrastructure data. Sea routes are based on the Python package *SeaRoute*[10], which utilizes *Eurostat* data[11] to calculate international shipping routes. Ports are based on the *World Bank*[12] database. *Supplementary Fig. 20* shows the existing infrastructure and shipping network.

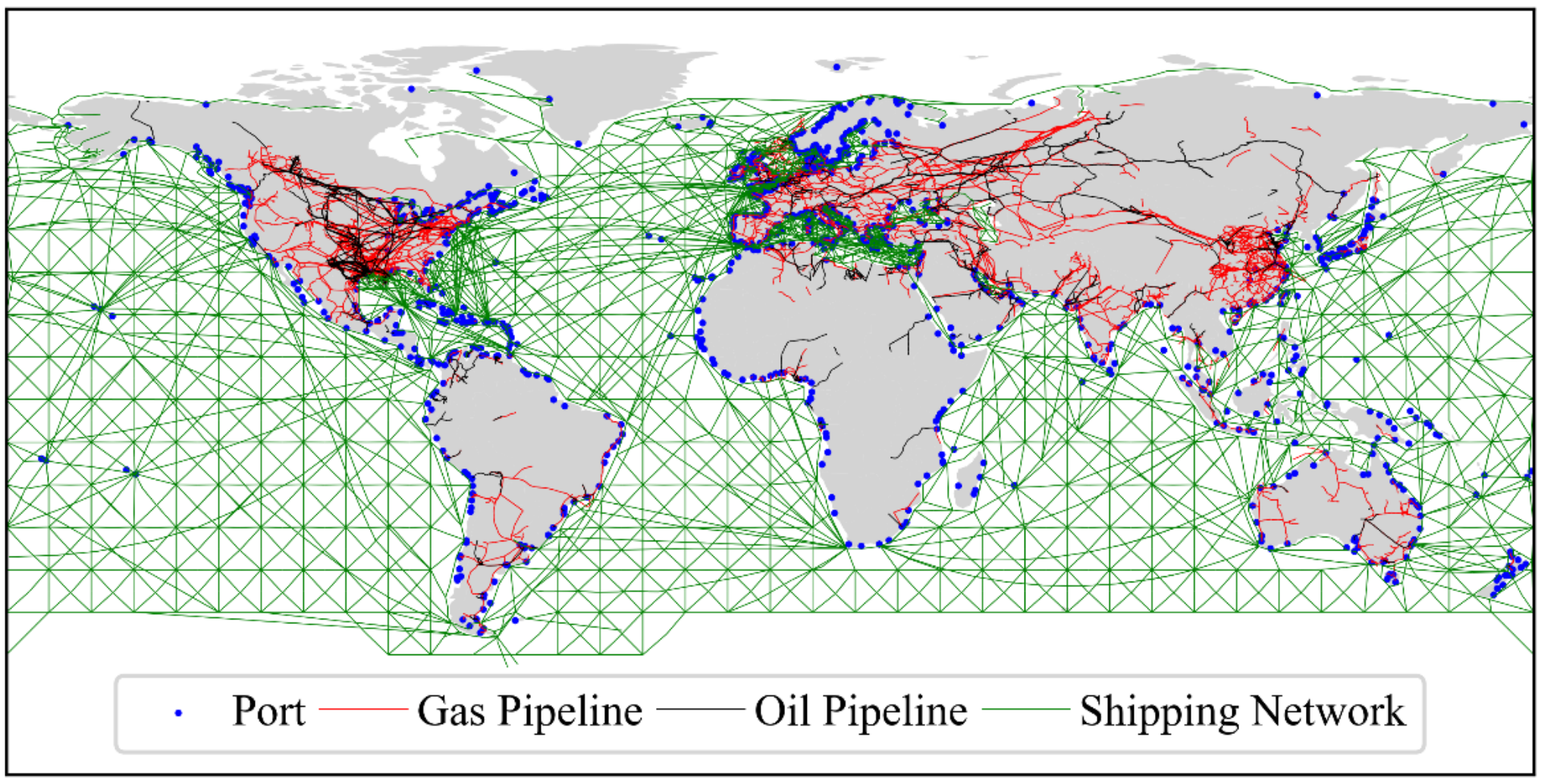

Supplementary Fig. 20. Depiction of port, gas, and oil pipeline data

The data is further processed to apply to the approach. Data exists as independent pipeline segments for pipelines, while more extensive networks are not reported. The individual segments are evaluated for connections and intersections to derive networks from the dataset, and connected segments are grouped into networks. Only such pipeline segments in operation or under construction are considered in this process. We increase the number of access points to the pipeline infrastructure to ensure at least one every 50 kilometers. The resulting networks are then modeled as undirected graphs where the access points and intersections represent the nodes, and the pipelines segment the edges, which are weighted by their length.

The infrastructure itself does not change during the execution of the main algorithm. Therefore, distances and shortest paths between infrastructures are consistent, and the number of shortest path options is limited. Taking advantage of this, the shortest paths within networks are calculated using the Dijkstra algorithm[13]. Distances between ports are derived using the Python package *SeaRoute*[10].

Supplementary Method 1

Pseudocode of the branching algorithm and implementation remarks

Initialize
-Iteration counter i=1
-Start branches $b \in B^i$ including only the node-commodity tuple $(n^s, c^s) \in \Omega_b^{NC}$
-Set the benchmarks for the cumulated costs $u_{n^s,n,c}$ based on pre-heuristic calculations for all $(n, c) \neq (n^s, c^s)$ and the initial costs $u_{n^s,n^s,c^s} = U_{n^s,n^s,c^s}$

---

while $B^i \neq \emptyset$:
  Conversion branching I
  for each branch $b \in B^i$, with $(\tilde{n}, \tilde{c}) \in \Omega_b^{NC}$ denoting the leaf of the current branch
    for each potential conversion edge $(\tilde{c}, c') \in \Omega_{\tilde{n}}^C$ at the current leaf
      if $u_{n^s,\tilde{n},c'} > \frac{u_{n^s,\tilde{n},\tilde{c}}+\vartheta_{\tilde{c},c'}^{CC}}{\eta_{\tilde{c},c'}}$:
        set $u_{n^s,\tilde{n},c'} = \frac{u_{n^s,\tilde{n},\tilde{c}}+\vartheta_{\tilde{c},c'}^{CC}}{\eta_{\tilde{c},c'}}$
      if $u_{n^s,\tilde{n},c'} < \min_{n^e \in N^e} \min_{c \in C_{n^e}^e} u_{n^s,n^e,c}$:
        Create new branch b′ consisting of the transport edges: $\Omega_{n,c,t,b}^a$, $\Omega_{t,c,b}^g$ and conversion edges $\Omega_{n,b}^C$of the existing branch and add to $B^{i,1}$
        Add current conversion edge and leaf to the new branch: $\Omega_{\tilde{n},b'}^C = \Omega_{\tilde{n},b}^C \cup (\tilde{c}, c')$, $\Omega_{b'}^{NC} = \Omega_b^{NC} \cup (\tilde{n}, c'))$
        Delete all existing branches $b \in B^i$, with $(\tilde{n}, c') \in \Omega_b^{NC}$
  Repeat conversion branching for each branch $b' \in B^{i,1}$ and add leaves to $B^{i,2}$
  Transport branching I (for air distance-based modeling of new pipelines and road routes)
  for each branch $b \in (B^i \cup B^{i,1} \cup B^{i,2})$, where $(\tilde{n}, \tilde{c}) \in \Omega_b^{NC}$ is a leaf of the current branch
    for each potential transport edge $(\tilde{n}, n') \in \Omega_{\tilde{n},\tilde{c},t,b}^a$
      if $u_{n^s,n',\tilde{c}} > u_{n^s,\tilde{n},\tilde{c}} + \min_{t \in T^a} \vartheta_{\tilde{n},n',\tilde{c},t}^{aTC}$:
        set $u_{n^s,n',\tilde{c}} = u_{n^s,\tilde{n},\tilde{c}} + \min_{t \in T^a} \vartheta_{\tilde{n},n',\tilde{c},t}^{aTC}$
      if $u_{n^s,\tilde{n},c'} < \min_{c \in C_{n^e}^e} u_{n^s,n^e,c}$:
        Create new branch b′ consisting of the transport edges: $\Omega_{n,c,t,b}^a$, $\Omega_{t,c,b}^g$ and conversion edges $\Omega_{n,b}^C$of the existing branch and add to $B^{i,3}$
        Add current transport edge and leaf to the new branch: $\Omega_{\tilde{n},\tilde{c},t,b'}^a = \Omega_{\tilde{n},\tilde{c},t,b}^a \cup (\tilde{n}, n')$, $\Omega_{b'}^{NC} = \Omega_b^{NC} \cup (n', \tilde{c})$
        Delete all existing branches $b \in (B^i \cup B'^1 \cup B^{i,2})$, with $(\tilde{n}, \tilde{c}) \in \Omega_b^{NC}$
  Conversion branching II for $b \in B^{i,3}$ analog to **Conversion branching I** defining $B^{i,4}$ and $B^{i,5}$
  Transport branching II (for graph-based modeling of existing pipelines and shipping routes)
  for each branch $b \in (B^i \cup B'^1 \cup B^{i,2} \cup B^{i,3} \cup B^{i,4} \cup B^{i,5})$, with $(\tilde{n}, \tilde{c}) \in \Omega_b^{NC}$ denoting the leaf of the current branch
    for each potential transport edge $(\tilde{n}, n') \in \Omega_{t,c}^g$ at the current leaf
      if $u_{n^s,n',\tilde{c}} > u_{n^s,\tilde{n},\tilde{c}} + \min_{t \in T^g} \vartheta_{\tilde{n},n',\tilde{c},t}^{gTC}$:
        set $u_{n^s,n',\tilde{c}} = u_{n^s,\tilde{n},\tilde{c}} + \min_{t \in T^g} \vartheta_{\tilde{n},n',\tilde{c},t}^{aTC}$
      if $u_{n^s,\tilde{n},\tilde{c}} < \min_{n^e \in N^e} \min_{c \in C_{n^e}^e} u_{n^s,n^e,c}$:
        Create new branch b′ consisting of the transport edges: $\Omega_{n,c,t,b}^a$, $\Omega_{t,c,b}^g$ and conversion edges $\Omega_{n,b}^C$of the existing branch and add to $B^{i,6}$: $\Omega_{t,\tilde{c},b'}^g = \Omega_{t,\tilde{c},b}^g \cup (\tilde{n}, n')$, $\Omega_{b'}^{NC} = \Omega_b^{NC} \cup (n', \tilde{c})$
  Set and iteration update: $B^{i+1} = B^{i,6}$ and i=i+1

The pseudocode is an approach to heuristically implementing the mixed-integer optimization program. However, this pseudo-code will still require a huge computation time to solve the problem. To increase efficiency, we implement the approach with parallel computation matrices to allow simultaneous processing of locations.

Constraining potential transport options

We constrain the approach by forbidding consecutive point-to-point transport for two reasons: First, if a maximal distance for point-to-point transport is set, consecutive point-to-point transport would allow breaching this maximal distance by adding connected point-to-point routes in each iteration. Second, if no maximal distance is implemented, point-to-point distance to any node is possible, and using detours via other nodes will not achieve lower costs than direct point-to-point transport. Furthermore, we constrain the approach by allowing only a single utilization of transport systems like pipelines for each branch. Since we apply Dijkstra to calculate the shortest paths through pipeline systems for all nodes of the pipeline system and create one branch for each node, we cover all possible routes at once. Consecutive traversing of the pipeline system will not result in cheaper costs and will only increase the number of necessary iterations.

Upper bounds and local benchmarks

We apply a pre-heuristic to calculate an upper bound for the main heuristic. The upper bound is a valid but not necessarily the most cost-efficient solution of the approach. It allows the termination of branches if they exceed the upper bound, even before they reach the destination. In this pre-heuristic, we use the combinations start + point-to-point + shipping + point-to-point + destination; start + point-to-point + pipeline + point-to-point + destination; start + point-to-point + pipeline + point-to-point + pipeline + point-to-point + destination; start + point-to-point + pipeline + point-to-point + shipping + point-to-point + destination and start + point-to-point + destination to create routes. For all routes, we always use the closest available infrastructure (closest port to start, closest pipeline system to destination, etc.) and all combinations of available commodities to cover these routes. Branches reaching the destination and having lower total supply costs than the upper bound will update the upper bound. Potential branches can be terminated based on the upper bound by using cost approximations. These include, for example,

calculating the cheapest transport costs of the residual route to the destination, or calculating minimal additional costs for using a target infrastructure (based on residual distance to infrastructure, necessary conversions to use infrastructure, etc.). Combined with the total costs of the traveled route, such costs can be compared to the upper bound. Finally, subproblems are processed to assess potential branches. For example, suppose several branches aim for a common infrastructure network, it might be useful to process the closest branch to the infrastructure first to calculate local benchmarks of the network nodes. Other branches might exceed these local benchmarks since their transportation costs might already be higher than the transportation costs of the branch closest to the infrastructure network.

Supplementary Method 2

Methods and data for the calculation of hydrogen production and electricity generation costs on high geographical resolution

ERA 5[14] uses a global grid with a resolution of 0.25°x 0.25° to provide high-resolution weather data. Using the open source tool atlite[15], hourly capacity factor profiles for wind and solar PV electricity generation of 11 years are derived for each grid cell and clustered to obtain representative data. Other electricity generation technologies are not considered since we aim for SECs that meet the requirements to count as renewable fuels of non-biological origin (RFNBO)[16]. These requirements define the electricity origin to stem from renewable generators or low-carbon electricity grids, and the generators must be installed explicitly to produce RFNBOs, excluding already installed RES-E capacities or nuclear power. Large hydrogen storage options are unnecessary since we assume a steady hydrogen supply to conversion units, and the demand itself is implemented as total demand per year without considering temporal volatility. A mixed-integer linear optimization is applied to calculate the minimal production costs of 1 GWh of hydrogen for each ERA 5 cell. The optimization minimizes production costs based on annualized investments, fixed and variable operation and maintenance of each component, and purchase, revenues, and disposal costs. The decision variables include individual capacities, each component's operation (conversions, storage, generation), and decisions on purchasing, selling, and disposal. The constraints represent the technical properties of all components (e.g., ramping constraints, minimal and maximal load, efficiencies of conversions, storage operation) and the exchange with markets (purchase, selling, disposal) and environment (emissions, use of ambient air, RES-E generation capacity factors). The operation of the facility and all exchanges with markets and the environment have an hourly resolution, allowing the consideration of the RES-E generation volatility. We do not consider scaling factors for hydrogen production via water electrolysis, making the model linear. This enables scaling the optimal capacities to capacity potentials to calculate hydrogen production potentials while maintaining minimal production costs. These minimal production costs are integrated into our approach to assess the relation between hydrogen production, conversion to other commodities, and transport, and to provide insights into potential import costs to the destination. A detailed explanation of the calculation of hydrogen production costs can be found in Langenmayr et al.[17]. The country-specific WACCs are based on the work of

Kleinschmitt et al.[18]. Since the data are based on the year 2021, we used the correlation between the given WACC and the worldwide governance indicators[19] to consider the development of 2022 (e.g., Russo-Ukrainian War). The country-specific WACC values used in the analysis are provided in the Supplementary Data file. The production of hydrogen is assumed to take place at the location of electricity generation using a stand-alone production facility, excluding the option of large-scale electricity transmission in our model. The hydrogen production system comprises a proton exchange membrane (PEM) electrolysis, battery storage, and wind and solar PV generator (techno-economic data in *Supplementary Table 1*). The production plant is depicted in *Supplementary Fig. 21*. *Supplementary Fig. 22* shows the global hydrogen production costs.

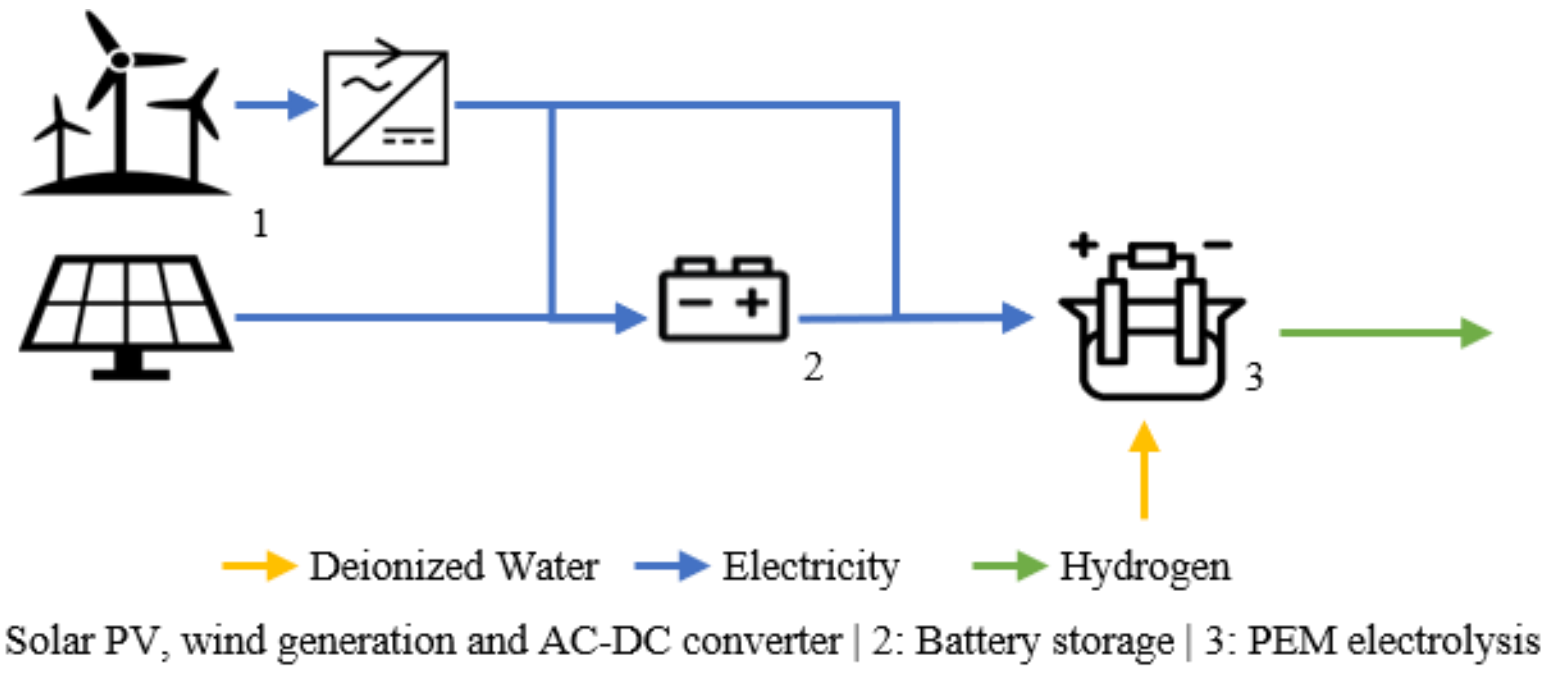

Supplementary Fig. 21. System boundaries of the implemented hydrogen production plant. Figure based on Langenmayr (2025)[17]

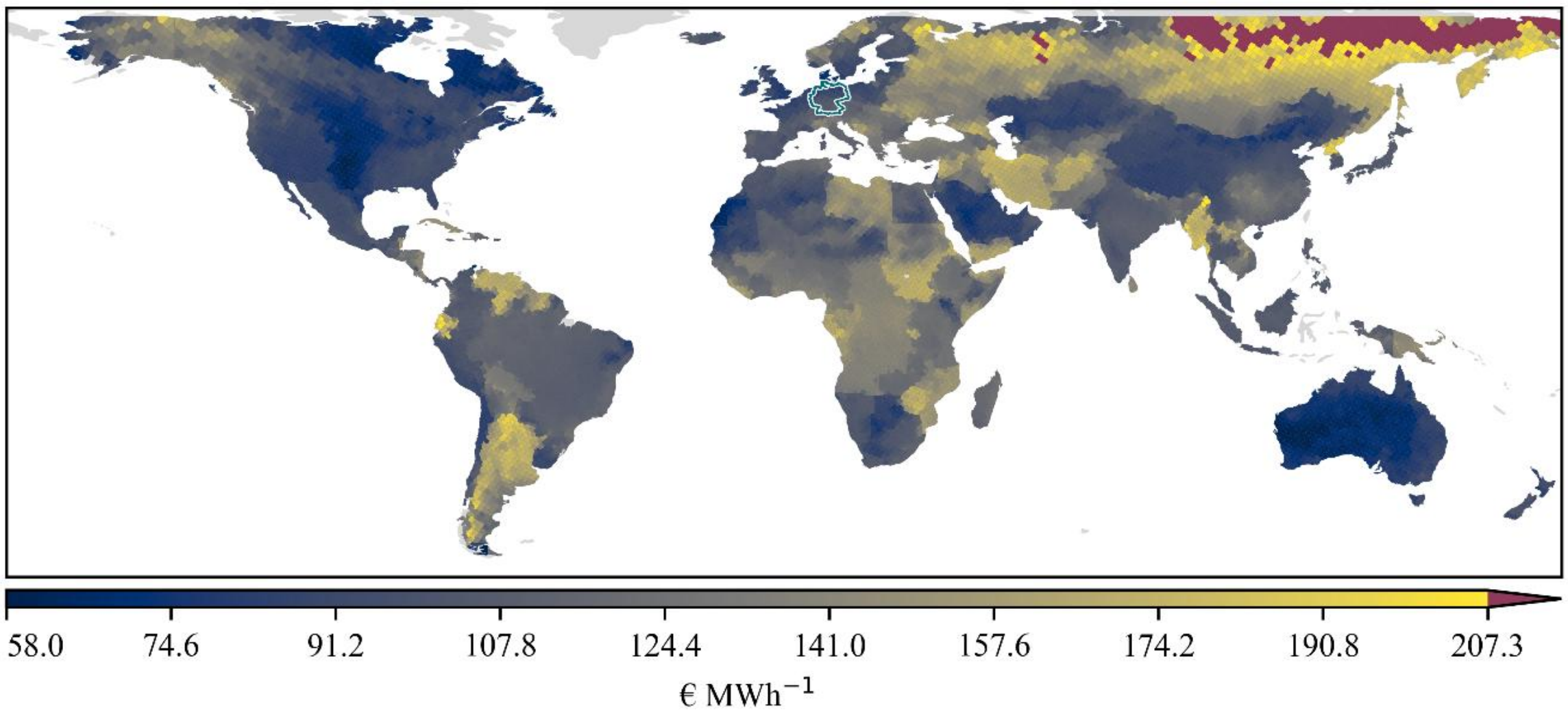

Supplementary Fig. 22. Hydrogen production costs for all grid cells

In addition to hydrogen production, electricity is needed to supply the synthesis processes for the conversion of hydrogen to other SECs. We use the same weather and techno-economic data to calculate electricity generation costs for each grid cell. Since the electricity is only used to supply the synthesis units, we smooth the electricity output to achieve a steady supply, since synthesis units are less flexible and process conditions must be maintained to achieve desired efficiencies and outputs. Smoothed electricity output is achieved by implementing a dummy conversion with an efficiency of 100%, without any investment or operating costs, but with a minimal load of 80%. The optimization model's output electricity curve will operate between 80% and 100%. However, we only use the resulting minimal generation costs to calculate conversion costs. The setup of the electricity generation is shown in *Supplementary Fig. 23*.

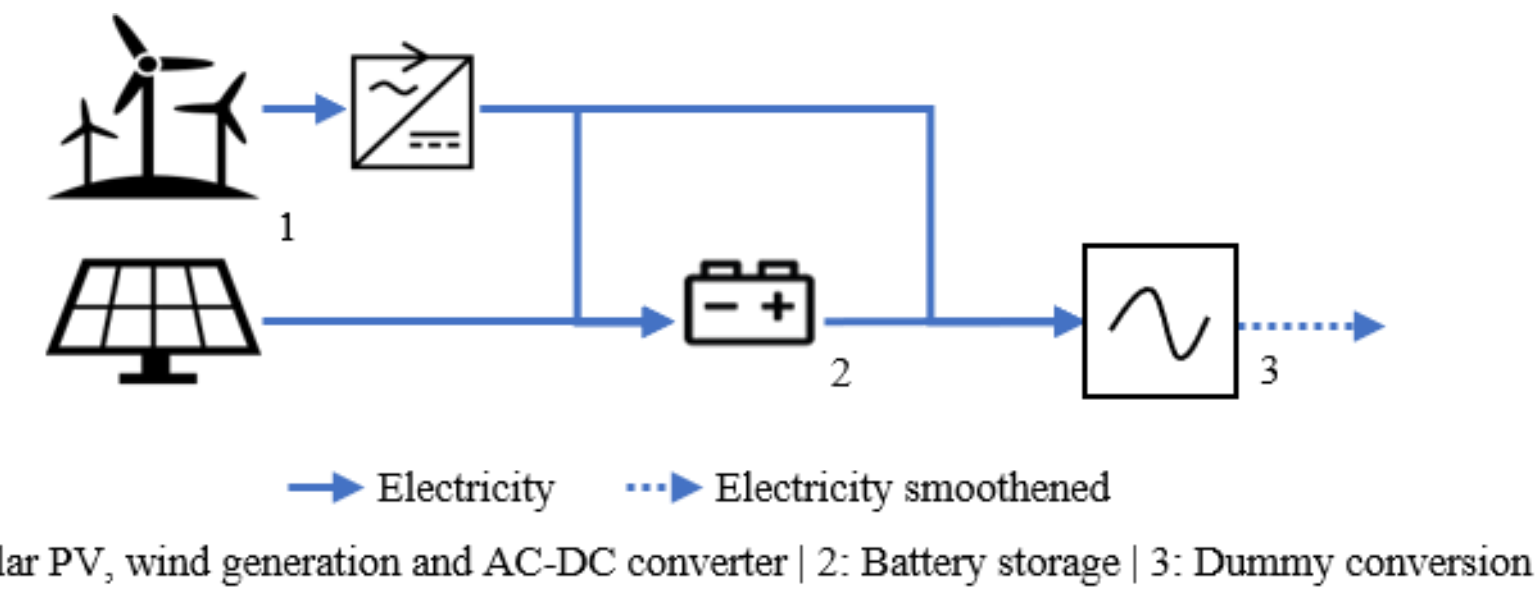

Supplementary Fig. 23. System boundaries of the implemented electricity generation. Figure based on Langenmayr (2025)[17]

Supplementary Table 1. Techno-economic assumptions on wind and solar generators[20], PEM electrolysis[21], and battery storage[22]. Abbreviations: O&M = Operation and Maintenance, PEM = Proton Exchange membrane

Technology	Wind	Solar	PEM electrolysis	Battery storage
Nominal investment	1,040 [€ kW^{-1}]	310 [€ kW^{-1}]	810 [€ kW^{-1}]	330 [€ kWh^{-1}]
Fixed O&M [%]	1.21	1	4	5.645
Variable O&M [€ kWh^{-1}]	0.00135	0	0	0.00112
Lifetime [years]	25	30	20	20
Conversion efficiency [%]	-	-	62	-
Minimal load [%]	-	-	7	-
Minimal state of charge [%]	-	-	-	10
Maximum state of charge [%]	-	-	-	90
Round-trip efficiency [%]	-	-	-	85

The uncertainty of renewable generation concerning volatility is typically considered using stochastic or robust optimization methods. Neither sensitivity analysis nor advanced optimization methods have been

applied in this work, as substantial computation time increases accompany these methods since they intensify the complexity of the optimization.

Supplementary Method 3

Solar PV and wind onshore generation capacity potentials

The definition of the global ground-mounted PV and wind onshore capacity potentials follows the approach described in Slednev et al. (2018)[23] for Europe and extended by Slednev (2024)[24] on a global scale. The general aim of this publication was to define the remaining technical potential for new installations in 2030 concerning land cover suitability restrictions and to exclude areas already exploited for these generation types in 2023. While a detailed description of the various processing steps, the utilized data, and the assumed restrictions are given in Slednev (2024)[24], the main data sources and restrictions for defining the potentials are listed in *Supplementary Table 2*. Regarding excluding existing wind onshore and ground-mounted PV installations, we relied on an internal database of existing power generators, which is parametrized from various sources, including an open street map, Global Energy Monitor, and IRENA 2023, among others. The present study uses the renewable generation capacity potentials reported in that publication without recalculating them.

Supplementary Table 2. Central assumptions and parameters for the calculation of global solar PV and wind generation capacities

Land use-related restrictions based on Copernicus Global Land Cover		
LandCoverClass	Wind: [Buffer [m], Suitability [-]]	PV: [Buffer [m], Suitability [-]]
Closed forest, evergreen needleleaf	[-, 0.100]	[300, 0]
Closed forest, deciduous needle leaf	[-, 0.100]	[300, 0]
Closed forest, evergreen, broadleaf	[-, 0.100]	[300, 0]
Closed forest, deciduous broadleaf	[-, 0.100]	[300, 0]
Closed forest, mixed	[-, 0.100]	[300, 0]
Closed forest, unknown	[-, 0.100]	[300, 0]
Open forest, evergreen needleleaf	[-, 0.100]	[300, 0]
Open forest, deciduous needle leaf	[-, 0.100]	[300, 0]
Open forest, evergreen broadleaf	[-, 0.100]	[300, 0]
Open forest, deciduous broadleaf	[-, 0.100]	[300, 0]
Open forest, mixed	[-, 0.100]	[300, 0]
Open forest, unknown	[-, 0.100]	[300, 0]
Shrubs	[-, 0.650]	[-, 0100]
Herbaceous vegetation	[-, 0.650]	[-, 0100]
Herbaceous wetland	[1000, 0.100]	[1000, 0]
Moss and lichen	[-, 0.650]	[-, 0.325]
Bare / sparse vegetation	[-, 0.800]	[-, 0.400]
Cultivated and managed vegetation/agriculture (cropland)	[-,0.525]	[-,0.262]
Urban/built-up	[1000,0]	[-,0]
Snow and Ice	[-, 0]	[-, 0]
Permanent water bodies	[400,0]	[-,0]
Open sea	[1000,0]	[1000,0]
Topology-related restrictions based on GTOPO30		
Type	Wind	PV
Altitude	>1800m	>2500m
Slope	>20°	>15° & > 3° if not in SW to SE orientated on NH or NW to NE in SH
Infrastructure-related restrictions based on OpenStreetMap		
Type	Wind: Buffer [m]	PV: Buffer [m]
Pipeline	150	150
Highway	200	0
Overhead line	200	0
Rail	150	0
Waterway	400	100
Protected areas related restrictions based on UNEP-WCMC and IUCN (2021)		
Type	Wind: Buffer [m]	PV: Buffer [m]
All	1000	150

derives the optimal relationship between solar PV and wind generator capacities to minimize hydrogen production costs. Using this relation together with the solar PV and wind generator capacity potentials, the hydrogen production potential produced at these optimal costs can be calculated. This potential amounts to approximately 520,000 TWh globally. The distribution of these potentials is shown in *Supplementary Fig. 24*.

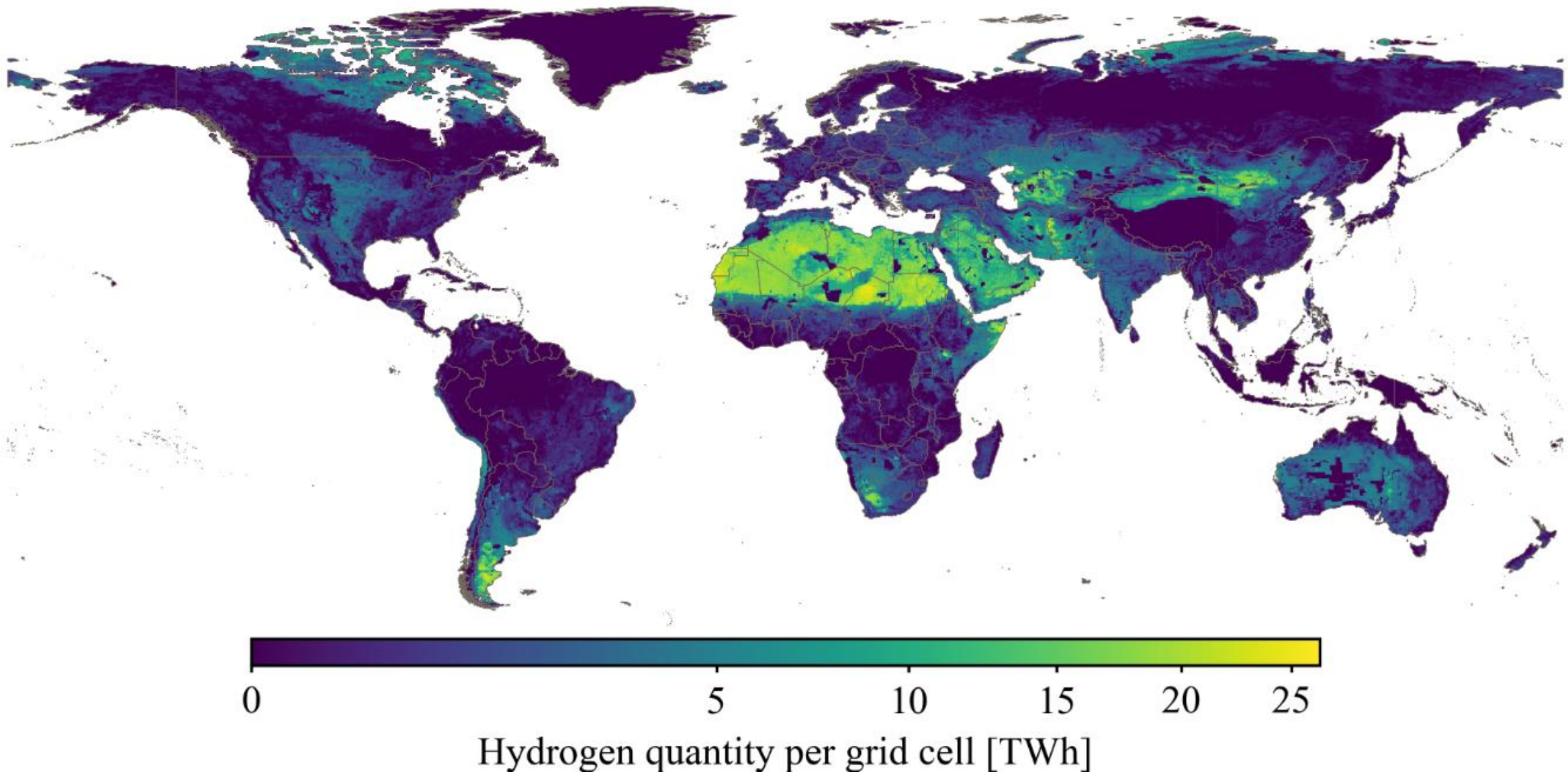

Supplementary Fig. 24: Hydrogen production potential per grid cell. These potentials are based on the optimal relationship between solar PV and wind generator capacities when minimizing hydrogen production costs.

Supplementary Method 4

Methods and data for the calculation of conversion costs

Supplementary Equation (1) shows the method for calculating the specific costs of conversion processes in euros per megawatt hour energy content of the respective product, based on its lower heating value (LHV). Included are investment-related costs, CAPEX, annual operation expenditures, OPEX, as a percentage of CAPEX (*Supplementary Table 4*), and costs for respective precursors (nitrogen, carbon dioxide, and electricity). An annuity factor, ANF, is used to calculate annuities as presented in *Supplementary Equation (2),* where *dp* stands for the depreciation period of the respective infrastructure in years and WACC for the weighted average costs of capital.

$$Conversion\ costs\ [€\ MWh^{-1}] = \frac{CAPEX}{Operating\ hours\ p.a.} * (ANF + OPEX) + (electricity, N_2, CO_2)\ demand * costs \quad (1)$$

$$ANF\ [a^{-1}] = \frac{WACC * (1 + WACC)^{dp}}{(1 + WACC)^{dp} - 1} \quad (2)$$

In all scenarios except for *All-Retrofit-Heat*, the supply of heat required for endothermal dehydrogenation or cracking processes is modeled through autothermal processes by combusting the feedstock/reactant, except in the case of DBT and MCH dehydrogenation, where the product hydrogen is combusted. This results in a proportional reduction in the material stream corresponding to the required heat. *Supplementary Equation (3)* shows the conversion efficiency calculation owing to reaction heat provision for ammonia cracking, methanol and methane steam reforming, or for DBT and MCH dehydrogenation. Pre-heating demands of exothermal synthesis processes are neglected, assuming process-internal heat recovery.

$$\eta_{c,c'} := \begin{cases} 1 - \dfrac{\text{heat demand}}{\text{heating value } H_2} & \text{for DBT and MCH dehydrogenation} \\ 1 - \dfrac{\dfrac{\text{heat demand}}{\text{heating value}}}{\text{stoichiometric ration } + \dfrac{\text{heat demand}}{\text{heating value}}} & \text{else} \end{cases} \quad (3)$$

In the *All-Retrofit-Heat* scenario, waste heat is assumed to be available to all processes at all locations. If this option is set in the configuration, no additional heat is needed from autothermal processes, and the conversion efficiency is 1 for all processes.

Supplementary Table 3 indicates the costs associated with conversion processes between all SECs, based on literature (*Supplementary Table 4*) and calculated as in *Supplementary Equation (1)*, targeting 2030. Since uniform WACC and electricity costs are used to derive the parameters presented in *Supplementary Table 3*, while location-specific values are applied in the heuristic, this table provides an illustrative approximation for a representative hypothetical location. Nitrogen (55 € $t^{-1}_{N_2}$[25]) and carbon dioxide costs (145 € $t^{-1}_{CO_2}$[25]) are uniform for all cases. When converting from one hydrogen derivative to another, it is assumed that it is converted to hydrogen first, and then the synthesis happens in the next step. FTC is not expected to be further converted. All conversion plants have the same annual utilization of 8,000 h. The WACCs are based on the values in the Supplementary Data file. Material costs of the LOHC substances DBT and MCH are neglected following the assumption that they can be recycled indefinitely.

Supplementary Table 3. Approximation of conversion costs in € MWh^{-1}_{Prod} based on uniform electricity generation (45 € $MWh^{-1}_{Electricity}$), nitrogen (55 € $t^{-1}_{N_2}$), and carbon dioxide costs (145 € $t^{-1}_{CO_2}$[25]), as well as a WACC of 6%. Values are calculated using *Supplementary Equation (1)* and parameter assumptions presented in *Supplementary Table 4*. Abbreviations: G = gaseous; L = liquid; MeOH = Methanol; FTC = Fischer-Tropsch crude; DBT = Dibenzyltoluene; MCH = Methylcyclohexane

from	to	Costs [€ MWh^{-1}_{Prod}]	from	to	Costs [€ MWh^{-1}_{Prod}]	from	to	Costs [€ MWh^{-1}_{Prod}]
G-H_2	L-H_2	40.5	G-H_2	MCH	2.8	G-CH_4	G-H_2	6.0
G-H_2	NH_3	18.6	G-H_2	FTC	81.4	DBT	G-H_2	4.6
G-H_2	MeOH	48.9	L-H_2	G-H_2	0.9	MCH	G-H_2	8.1
G-H_2	G-CH_4	32.6	NH_3	G-H_2	5.2	G-CH_4	L-CH_4	12.1
G-H_2	DBT	4.2	MeOH	G-H_2	4.6	L-CH_4	G-CH_4	0.004

The column “Loss of Material” in *Supplementary Table* 4 illustrates the material loss during conversion steps. Bold values are losses due to autothermal processes, calculated using *Supplementary Equation (3)* based on the heat requirements listed in *Supplementary Table* 4, column “Heat Demand”, for ammonia

cracking, methanol, and methane steam reforming (stoichiometric ratios for the respective chemical reactions: 5.63 kg_{NH3} kg^{-1}_{H2}, 5.29 kg_{MeOH} kg^{-1}_{H2}, and 2.65 kg_{CH4} kg_{H2}^{-1}), as well as for DBT and MCH dehydrogenation. Other values represent literature-based losses not related to the provision of reaction heat.

Supplementary Table 4. Literature values and references for the calculation of conversion costs. Abbreviations: G = gaseous; L = liquid; MeOH = Methanol; FTC = Fischer-Tropsch crude; DBT = Dibenzyltoluene; MCH = Methylcyclohexane. Bold values are losses due to autothermal processes.

	Capital costs CAPEX			Operating costs OPEX [$\%_{CAPEX}$ a^{-1}]		Deprec. period [a]		Electricity demand			Heat demand			Loss of material [%]		Precursor demand		
$GH_2 \rightarrow LH_2$	1,497,967	€ $MW^{-1}{}_{H2}$	26	8.00	26	20	26	6.78	MWh $t^{-1}{}_{H2}$	26	none			1.65	26			
$GH_2 \rightarrow NH_3$	15,000	k€ $(t_{H2}\ h^{-1})^{-1}$	25	3.00	27	20	25	1.5	MWh $t^{-1}{}_{H2}$	25	none/exotherm					4.63	$t_{N2}t^{-1}{}_{H2}$	25
$GH_2 \rightarrow$ MeOH	1,090	k€ $MW^{-1}{}_{MeOH}$	28	1.75	26	30	28	0.1	MWh $t^{-1}{}_{MeOH}$	28	none/exotherm					0.25	t_{CO2} $MWh^{-1}{}_{MeOH}$	26
$GH_2 \rightarrow$ G-CH_4	278	k€ $MW^{-1}{}_{CH4}$	26	4.00	26	30	26	none			none/exotherm					0.20	t_{CO2} $MWh^{-1}{}_{CH4}$	26
$GH_2 \rightarrow$ DBT	256	k€ $(30\ kg_{H2}\ h^{-1})^{-1}$	29	4.00	29	20	30	0.1	MWh $t^{-1}{}_{H2}$	29	none/exotherm			0.1	29			
$GH_2 \rightarrow$ MCH	157	k€ $(30\ kg_{H2}\ h^{-1})^{-1}$	29	4.00	29	20	30	0.2	MWh $t^{-1}{}_{H2}$	29	none/exotherm			0.1	29			
$GH_2 \rightarrow$ FTC	1,600	k€ $MW^{-1}{}_{FTC}$	26	3.00	26	25	26	0.02	MWh $MWh^{-1}{}_{FTC}$	26	none/exotherm					0.41	t_{CO2} $MWh^{-1}{}_{FTC}$	26
$LH_2 \rightarrow GH_2$	4,320	€ $MW^{-1}{}_{H2}$	26	3.00	26	10	26	0.6	MWh $t^{-1}{}_{H2}$	26	none							
$NH_3 \rightarrow GH_2$	8,800	k€ $(tH_2\ h^{-1})^{-1}$	25	3.00	27	20	25	0.95	MWh $t^{-1}{}_{H2}$	25	6.65	MWh $t^{-1}{}_{H2}$	27	**18.6**				
MeOH $\rightarrow GH_2$	16,318	€ $MW^{-1}{}_{H2}$	26	4.00	26	20	26	3.2	MWh $t^{-1}{}_{H2}$	26	4.50	MWh $t^{-1}{}_{H2}$	26	**12.7**				
G-$CH_4 \rightarrow GH_2$	470,085	€ $MW^{-1}{}_{H2}$	26	3.00	26	30	26	none			0.8	$kgCH_4$ $kg^{-1}{}_{H2}$	26	**23.2**				
DBT $\rightarrow GH_2$	282	k€ $(30\ kg_{H2}\ h^{-1})^{-1}$	29	4.00	29	20	30	0.1	MWh $t^{-1}{}_{H2}$	29	11.6	MWh $t^{-1}{}_{H2}$	30	**34.8**				
MCH $\rightarrow GH_2$	127	k€ $(30\ kg_{H2}\ h)^{-1}$	29	4.00	29	20	30	4.5	MWh $t^{-1}{}_{H2}$	29	13.5	MWh $t^{-1}{}_{H2}$	30	**40.5**				
GCH4 $\rightarrow$ L-CH_4	740	€ $kW^{-1}{}_{CH4}$	26	3.50	26	25	26	0.5	MWh $t^{-1}{}_{CH4}$	26	none							
L-$CH_4 \rightarrow$ G-CH_4	280	€ $MW^{-1}{}_{CH4}$	26	3.50	26	30	26	none			none			2.0	26			

Supplementary Method 5

Methods and data for the calculation of transport costs

The model includes ship transport for all liquid energy carriers and pipeline transport via existing or new pipelines for gaseous hydrogen, gaseous methane, and FTC. All energy carriers can be transported by truck, except for methane in gaseous form, due to a lack of techno-economic data. While shipping and pipeline transport costs are obtained from the literature, road transport costs *RTC* are calculated using *Supplementary Equation (4)*, with annual operating hours *aoh*, average speed *avs*, trailer capacity *trc*, fuel consumption *fuc*, fuel price *fup*, and hourly operating costs *hoc*. The trailer capacity *trc* is halved to account for the empty return trip.

$$RTC[€\ MWh^{-1}\ (1{,}000\ km)^{-1}] = \frac{[CAPEX \cdot (ANF + OPEX)]_{Truck} + [CAPEX \cdot (ANF + OPEX)]_{Trailer} + aoh \cdot (avs \cdot fuc \cdot fup + hoc)}{aoh \cdot avs \cdot trc/2} \quad (4)$$

For all transport means, we assume the marginal user approach. This means that the supplier of SECs is using existing infrastructure only by paying a certain "fee" without arranging investments. *Supplementary Table 5* displays the transport costs for all SECs and respective eligible modes of transport.

Supplementary Table 5. Transport costs in € MWh^{-1} $(1{,}000\ km)^{-1}$. Abbreviations: G = gaseous; L = liquid; MeOH = Methanol; FTC = Fischer-Tropsch crude; DBT = Dibenzyltoluene; MCH = Methylcyclohexane

	G-H_2	L-H_2	NH_3	MeOH	G-CH_4	L-CH_4	DBT	MCH	FTC
New pipeline	4.9	-	-	-	1.4	-	-	-	0.4
Existing pipeline	3.1	-	-	-	0.9	-	-	-	0.2
Ship incl. invest	-	1.4	0.7	0.5	-	0.9	1.3	1.3	0.1
Truck incl. invest	126.6	23.0	33.6	15.1	-	16.7	42.6	43.3	7.5

Pipeline transport costs are adopted from Saadi et al.[31], with a 36-inch pipeline diameter and flow velocities of 10 m s^{-1} for hydrogen and methane pipelines and 1 m s^{-1} for oil pipelines (used for FTC). The mean of the daily closing exchange rate, averaged over the publication year 2018, 0.85 € $US\$^{-1}$, is used for the conversion from dollar to euro. *Supplementary Table 6* contains literature values used for pipeline transport and liquid methane shipping. Other shipping costs are obtained from the Excel calculation tool provided for download with the publication of Staiß et al.[25] based on a shipping distance of 15,000 kilometers. Ship sizes range from a total tonnage of 75,000 t for chemical tankers handling ammonia,

methanol, DBT, and MCH to 130,000 t for liquid gas tankers for hydrogen and 318,000 t for huge crude carriers (VLCC) for FTC.

Parameters for the calculation of road transport costs *RTC* as in *Supplementary Equation (4)* are adopted from Niermann et al.[29], comprising 3,500 hours annual truck operation *aoh* at an average speed *avs* of 60 km h^{-1}, a 30 L $(100km)^{-1}$ fuel consumption *fuc*, a fuel price *fup* of 1.19 € L^{-1}, and hourly operating costs *hoc* of 35 € h^{-1}. *Supplementary Table 7* provides further literature values for calculating specific *RTC* for gaseous and liquid hydrogen, ammonia, DBT, and MCH. Since they are liquid hydrocarbons with similar handling requirements, methanol and FTC costs are derived from DBT/MCH *RTC* using heating values (*Supplementary Table 8*). An expert interview yielded truck transport costs for liquid methane.

Further assumptions are that the maximum distance for newly built pipelines is 100 km, since infrastructure planning is complex and larger interregional projects are beyond the scope. For road transport, we assume no maximum distance for our model runs. However, it is possible to implement this in the model. Not limiting the maximum distance of road transport allows for calculating transport costs even for remote locations with no infrastructure. For road transport and newly built pipeline infrastructure, distances are multiplied by 150% to account for obstacles in the route. This reduces the newly built pipelines' as-the-crow-flies distance to 2/3 of 100 km.

Supplementary Table 6. Literature values for the calculation of pipeline and methane ship transport costs (all Saadi et al.[31])

Transport costs [US$ km^{-1} J^{-1}]	
G-H_2	
New pipeline	$1.6/10^{12}$
Existing Pipeline	$(1.6-0.58)/ 10^{12}$
G-CH_4	
New pipeline	$0.47/10^{12}$
Existing Pipeline	$(0.47-0.18)/ 10^{12}$
FTC	
New pipeline	$0.12/10^{12}$
Existing Pipeline	$(0.12-0.04)/ 10^{12}$
L-CH_4	
Ship	$0.30/10^{12}$

Supplementary Table 7. Literature values for the calculation of truck transport costs (Niermann et al.[29], unless otherwise specified). Abbreviations: G = gaseous; L = liquid; MeOH = Methanol; FTC = Fischer-Tropsch crude; DBT = Dibenzyltoluene; MCH = Methylcyclohexane

	Capital costs CAPEX [€]		Operating costs OPEX [$\%_{CAPEX}$ a^{-1}]		Life span [a]		Trailer capacity [t]	
Truck	160,000		12		8		-	
Trailer								
G-H_2	550,000		2		12		0.72	
L-H_2	860,000		2		12		4.5	
NH_3	220,000	[32]	2	[32]	20	[32]	14.6	[32]
DBT	150,000		2		12		28.5	[33]
MCH	150,000		2		12		28.5	[33]
MeOH, FTC	analogous to DBT							

Supplementary Table 8. Lower heating values and hydrogen contents. Abbreviations: G = gaseous; L = liquid; MeOH = Methanol; FTC = Fischer-Tropsch crude; DBT = Dibenzyltoluene; MCH = Methylcyclohexane

	Lower heating value [kWh kg^{-1}]	Hydrogen content [wt.-%]
H_2	33.33	100
NH_3	5.17	17.7
MeOH	5.83	12.5
DBT	-	6.2
MCH	-	6.16
FTC	11.75	-
CH_4	13.9	25

Transportation of liquefied SECs implies boil-off losses, whereby part of the cooled cargo evaporates. In the model, this effect is represented for the multi-day maritime transport of liquid hydrogen, methane, and ammonia, as it reduces the quantity of SEC delivered to the import region. Depending on the propulsion system, boil-off may be used as marine fuel. Accordingly, two configurations are considered for ships transporting liquid hydrogen, methane, and ammonia: (i) vessels powered by fossil fuels, with boil-off losses vented, and (ii) vessels using boil-off, supplemented where necessary by cargo withdrawal, for propulsion. In the first case, transport costs result from fossil fuel consumption (*Supplementary Equation (5)*), and transport efficiency is reduced due to boil-off (*Supplementary Equation (6)*).

$$\begin{aligned} &Transport\ costs \\ &= (Commodity\ costs\ before\ transport\,[€\ MWh^{-1}] + Distance_t\,[1000\ km] \\ &\cdot specific\ fuel\ costs\ [€\ MWh^{-1} \cdot (1000\ km)^{-1}]) \cdot (1-\eta_t) \end{aligned} \quad (5)$$

$$\eta_t = 1 - \left(\frac{Distance_t[1000\ km] \cdot specific\ boil\ off\ [MWh\ (1000\ km)^{-1}]}{Transport\ volume\ of\ carrier[MWh]}\right) \quad (6)$$

In the second case, transport costs are driven by commodity losses (*Supplementary Equation (7)*), while efficiency is calculated according to *Supplementary Equations (8)-(10)*, including the case where boil-off exceeds propulsion demand.

$$Transport\ costs = Commodity\ costs\ before\ transport \cdot (1 - \eta_t) \quad (7)$$

$$\eta_{t,consumption} = 1 - \left(\frac{Distance_t[1000\ km] \cdot specific\ fuel\ consumption\ [MWh\ (1000\ km)^{-1}]}{Transport\ volume\ of\ carrier[MWh]}\right) \quad (8)$$

$$\eta_{t,boil-off} = 1 - \left(\frac{Distance_t[1000\ km] \cdot specific\ boil-off\ [MWh\ (1000\ km)^{-1}]}{Transport\ volume\ of\ carrier[MWh]}\right) \quad (9)$$

$$\eta_t = \min(\eta_{t,consumption}, \eta_{t,boil-off}) \quad (10)$$

For SECs that do not imply boil-off losses (FTC, DBT, MCH, methanol), ship propulsion is assumed to rely either on fossil fuels or, where technically feasible (FTC and methanol), on the transported commodity itself. In the latter case, transport costs are determined by the consumed share of the transported commodity, calculated as follows:

$$\eta_t = 1 - \left(\frac{Distance_t[1000\ km] \cdot specific\ fuel\ consumption\ [MWh\ (1000\ km)^{-1}]}{Transport\ volume\ of\ carrier[MWh]}\right) \quad (11)$$

$$Transport\ costs = Commodity\ costs\ before\ transport \cdot (1 - \eta_t) \quad (12)$$

Boil-off rates and fuel consumption are derived from ship parameters reported by Staiß et al.[25] and are summarized in *Supplementary Table 9*.

Supplementary Table 9. Ship parameters, fuel demand, and boil-off losses[25]. Abbreviations: G = gaseous; L = liquid; MeOH = Methanol; FTC = Fischer-Tropsch crude; DBT = Dibenzyltoluene; MCH = Methylcyclohexane

	$L\text{-}H_2$	$L\text{-}CH_4$	FTC	DBT	MCH	NH_3	MeOH
Supposed ship type	Q-Max LNG carrier		VLCC			Chemical tanker	
Capacity [m^3]	160,000		345,000			86,000	
Capacity [GWh]	378	1,001	3,000	651	545	278	401
Annuity, O&M, wages [€ a^{-1}]	22,564,988		9,485,908			6,901,372	
Average ship speed [km h^{-1}]	31		17			26	
Maintenance time [d a^{-1}]	30						
Port time [d $journey^{-1}$]	2						
Average journey [km]	15,000						
Av. yearly roundtrips [a^{-1}]	7		4			6	
Av. yearly outward travel [km a^{-1}]	105,000		60,000			90,000	
Allocated annuity, O&M, wages [€ km^{-1}]	215		158			77	
Port fees [€]	90,000		184,000			62,500	
Allocated port fees [€ km^{-1}]	12		25			8	
Total shipping costs [€ MWh^{-1} $(1000\ km)^{-1}$]	0.60	0.23	0.06	0.28	0.33	0.31	0.21
Cargo reduction for propulsion (assumption: boil-off + cargo is used for propulsion)							
Fuel demand [MWh km^{-1}]	1.36		0.99			0.48	
Fuel demand [MWh d^{-1}]	1,012		404			300	
Fuel demand [$\%_{capacity}$ km^{-1}]	0.00036	0.00014	0.00003	0.00015	0.00018	0.00017	0.00012
Boil-off [% d^{-1}]	0.2	0.1	0			0.01	0
Average boil-off [MWh d^{-1}]	741	991	0			28	0

The electricity transmission infrastructure is not considered as a transport option, which could improve our approach.[26,34] We have taken this decision since the consideration would shift the focus from the transportation and supply of SECs to a broader view of the energy transition across all sectors. In addition, the model and parameter uncertainties of such a large-scale model could further affect the quality of the transport model.

Supplementary Method 6

Methods and data for the calculation of commodity prices

In the *GER-All-Retrofit-Prices* scenario, commodity prices are applied to reflect the value of SECs in the heuristic. We excluded LOHCs since they are not set as target commodities in the case studies. The commodity prices are calculated for all SECs using the following Equation:

$$p_{c\prime} = \frac{\left(Natural\ Gas\ Price + Conversion\ Costs_{Methane,c\prime}\right)}{\eta_{Methane,c\prime}} + CO_2\ Emissions_{Methane,c\prime} \cdot p_{CO2} \quad (13)$$

The process includes converting natural gas to gaseous hydrogen, followed by conversion to hydrogen derivatives. The specific CO_2 emissions are based on life cycle assessment data reported in the literature (*Supplementary Table 10*). They differ because the production of some hydrogen derivatives uses CO_2 to convert hydrogen into hydrocarbons. For liquefied hydrogen, CO_2 emissions are the same as for gaseous hydrogen, since the liquefaction process uses electricity assumed to be fully renewable and no additional emissions occur.

Supplementary Table 10. Specific CO_2 emissions of the conversion from natural gas to the target commodity. Abbreviations: G = gaseous; L = liquid; MeOH = Methanol

Commodity	G-H_2	L-H_2	L-CH_4	MeOH	NH_3
Specific CO_2 emissions [t_{CO2} MWh^{-1}]	0.255[35]	0.255[35]	0.023[36]	0.1[37]	0.503[38]

We use the natural gas and CO_2 prices stated in the ENTSO-E Ten-Year Network Development Plan[39] for the year 2030. Conversion costs and efficiencies are based on our own calculations in *Supplementary Method 4*. *Supplementary Table 11* shows the final commodity prices. Methane and FTC prices are based on their fossil counterparts.

Supplementary Table 11. Final commodity prices in € MWh^{-1} and applied CO_2 price in € t^{-1}_{CO2}. Abbreviations: G = gaseous; L = liquid; MeOH = Methanol; FTC = Fischer-Tropsch crude; DBT = Dibenzyltoluene; MCH = Methylcyclohexane

Commodity	G-CH_4	G-H_2	L-H_2	L-CH_4	MeOH	NH_3	FTC	CO_2
Price	22.68[39]	66.30	66.30	47.90	110.16	119.71	33.12[39]	113.4[39]
Unit	€ MWh^{-1}							€ t^{-1}_{CO2}

Supplementary References

1. Langenmayr, U. Techno-Economic and Environmental Assessment of Electricity-Based Renewable Hydrogen and its Derivatives. (Karlsruher Institut für Technologie (KIT), 2026). https://doi.org/10.5445/IR/1000192602.

2. Magnific. *Tank Truck Icon*. Platform: Flaticon. https://www.flaticon.com/free-icons/tank-truck (last accessed: 21.9.2026).

3. Magnific. *Pipeline Icon*. Platform: Flaticon. https://www.flaticon.com/free-icons/pipeline (last accessed: 21.9.2026).

4. Magnific. *Ship Icon*. Platform: Flaticon. https://www.flaticon.com/free-icons/ship (last accessed: 21.9.2026).

5. World Resources Institute. Water risk atlas. (2025) URL: https://www.wri.org/aqueduct (last accessed: 27.5.2025). Licensed under Creative Commons Attribution 4.0 International Public License.

6. World Resources Institute. Aqueduct 3.0 Current and Future Global Maps Data. (2019) URL: https://wri-projects.s3.amazonaws.com/Aqueduct30/finalData/Y2019M07D12_Aqueduct30_V01.zip (last accessed: 18.5.2026). Licensed under Creative Commons Attribution 4.0 International Public License.

7. Gurobi Optimization, LLC. Gurobi Optimizer Reference Manual. (2026) URL: https://www.gurobi.com.

8. Global Energy Monitor. Global Oil Infrastructure Tracker [Data]. (2023) URL: https://globalenergymonitor.org/projects/global-oil-infrastructure-tracker/ (last accessed: 1.7.2024). Licensed under Creative Commons Attribution 4.0 International Public License.

9. Global Energy Monitor. Global Gas Infrastructure Tracker [Data]. (2023) URL: https://globalenergymonitor.org/projects/global-gas-infrastructure-tracker/ (last accessed: 1.7.2024). Licensed under Creative Commons Attribution 4.0 International Public License.

10. genthalili. SeaRoute [Software]. (2022) URL: https://github.com/genthalili/searoute-py/tree/main (last accessed: 1.7.2024). Licensed under Apache-2.0.

11. Eurostat. SeaRoute [Software]. (2021) URL: https://github.com/eurostat/searoute (last accessed: 1.7.2024). Licensed under EUPL-1.2.

12. The World Bank. Global - International Ports [Data]. (2020) URL: https://datacatalog.worldbank.org/search/dataset/0038118/Global---International-Ports (last accessed: 1.7.2024). Licensed under Creative Commons Attribution 4.0 International Public License.

13. Hagberg, A., Schult, D. & Swart, P. Exploring Network Structure, Dynamics, and Function using NetworkX. *Proceedings of the 7th Python in Science conference (SciPy 2008), G Varoquaux, T Vaught, J Millman (Eds.), pp. 11-15* (2008).

14. Hersbach, H. *et al.* The ERA5 global reanalysis. *Quart J Royal Meteoro Soc* **146**, 1999–2049 (2020) https://doi.org/10.1002/qj.3803.

15. Hofmann, F., Hampp, J., Neumann, F., Brown, T. & Hörsch, J. atlite: A Lightweight Python Package for Calculating Renewable Power Potentials and Time Series. *JOSS* **6**, 3294 (2021) https://doi.org/10.21105/joss.03294.

16. European Union. *Directive (EU) 2018/2001 of the European Parliament and of the Council of 11 December 2018 on the Promotion of the Use of Energy from Renewable Sources (Recast)*. *OJ L 328 21.12.2018* (2024).

17. Langenmayr, U. *et al.* Stand-alone power-to-X production dynamics: A multi-method approach to quantify the emission-cost reduction trade-off. *J of Industrial Ecology* **29**, 1791–1805 (2025) https://doi.org/10.1111/jiec.70085.

18. Kleinschmitt, C. *et al.* Weltweite Potenziale erneuerbarer Energien - HYPAT Working Paper 03/2022. *Fraunhofer ISI* (2022) https://doi.org/10.24406/publica-177.

19. Kaufmann, D. & Kraay, A. *Worldwide Governance Indicators - 2023 Update*. (2023) https://www.worldbank.org/en/publication/worldwide-governance-indicators (last accessed: 2.9.2024).

20. Danish Energy Agency. *Technology Data for Generation of Electricity and District Heating*. (2023) https://ens.dk/en/analyses-and-statistics/technology-data-generation-electricity-and-district-heating (last accessed: 1.7.2024).

21. Smolinka, T. *et al. Studie IndWEDe: Industrialisierung Der Wasserelektrolyse in Deutschland: Chancen Und Herausforderungen Für Nachhaltigen Wasserstoff Für Verkehr, Strom Und Wärme: Im Auftrag von Bundesministerium Für Verkehr Und Digitale Infrastruktur (BMVI)*. (2018) https://www.now-gmbh.de/wp-content/uploads/2020/09/indwede-studie_v04.1.pdf (last accessed: 1.7.2024).

22. Cole, W. & Karmakar, A. *Cost Projections for Utility-Scale Battery Storage: 2023 Update*. (2023) https://www.osti.gov/biblio/1984976 (last accessed: 27.6.2024).

23. Slednev, V., Bertsch, V., Ruppert, M. & Fichtner, W. Highly resolved optimal renewable allocation planning in power systems under consideration of dynamic grid topology. *Computers & Operations Research* **96**, 281–293 (2018) https://doi.org/10.1016/j.cor.2017.12.008.

24. Slednev, V. Development of a techno-economic energy system model considering the highly resolved conversion and multimodal transmission of energy carriers on a global scale. (2024) https://doi.org/10.5445/IR/1000170863.

25. Staiß, F. *et al. Optionen Für Den Import Grünen Wasserstoffs Nach Deutschland Bis Zum Jahr 2030: Transportwege - Länderbewertungen - Realisierungserfordernisse*. (acatech - Deutsche Akademie der Technikwissenschaften, München; Halle (Saale); Mainz, 2022). https://doi.org/10.48669/esys_2022-8.

26. Hampp, J., Düren, M. & Brown, T. Correction: Import options for chemical energy carriers from renewable sources to Germany. *PLoS ONE* **20**, e0314578 (2025) https://doi.org/10.1371/journal.pone.0314578.

27. Collis, J. & Schomäcker, R. Determining the Production and Transport Cost for H2 on a Global Scale. *Front. Energy Res.* **10:909298**, (2022) https://doi.org/10.3389/fenrg.2022.909298.

28. Danish Energy Agency. *Technology Data for Renewable Fuels: Version 10, 08/2023*. (2023) https://ens.dk/en/analyses-and-statistics/technology-data-renewable-fuels (last accessed: 1.7.2024).

29. Niermann, M., Timmerberg, S., Drünert, S. & Kaltschmitt, M. Liquid Organic Hydrogen Carriers and alternatives for international transport of renewable hydrogen. *Renewable and Sustainable Energy Reviews* **135**, 110171 (2021) https://doi.org/10.1016/j.rser.2020.110171.

30. Johnston, C., Ali Khan, M. H., Amal, R., Daiyan, R. & MacGill, I. Shipping the sunshine: An open-source model for costing renewable hydrogen transport from Australia. *International Journal of Hydrogen Energy* **47**, 20362–20377 (2022) https://doi.org/10.1016/j.ijhydene.2022.04.156.

31. Saadi, F. H., Lewis, N. S. & McFarland, E. W. Relative costs of transporting electrical and chemical energy. *Energy Environ. Sci.* **11**, 469–475 (2018) https://doi.org/10.1039/C7EE01987D.

32. International Energy Agency (IEA). *The Future of Hydrogen: Seizing Today's Opportunities*. (2019) https://www.iea.org/reports/the-future-of-hydrogen (last accessed: 1.7.2024).

33. Reuß, M. E. *Techno-Economic Analysis of Hydrogen Infrastructure Alternatives*. (RWTH Aachen University, 2019). https://doi.org/10.18154/RWTH-2019-07432.

34. Neumann, F., Zeyen, E., Victoria, M. & Brown, T. The potential role of a hydrogen network in Europe. *Joule* **7**, 1793–1817 (2023) https://doi.org/10.1016/j.joule.2023.06.016.

35. Cho, H. H., Strezov, V. & Evans, T. J. Environmental impact assessment of hydrogen production via steam methane reforming based on emissions data. *Energy Reports* **8**, 13585–13595 (2022) https://doi.org/10.1016/j.egyr.2022.10.053.

36. International Energy Agency (IEA). *Assessing Emissions from LNG Supply and Abatement Options*. (2025) https://iea.blob.core.windows.net/assets/df9b1bed-4e16-4db8-bec5-a5fa117a9130/AssessingemissionsfromLNGsupplyandabatementoptions.pdf (last accessed: 12.6.2026).

37. Methanol Institute. *Carbon Footprint of Methanol*. (2022) https://www.methanol.org/wp-content/uploads/2022/01/CARBON-FOOTPRINT-OF-METHANOL-PAPER_1-31-22.pdf (last accessed: 12.6.2026).

38. Liu, X., Elgowainy, A. & Wang, M. Life cycle energy use and greenhouse gas emissions of ammonia production from renewable resources and industrial by-products. *Green Chem.* **22**, 5751–5761 (2020) https://doi.org/10.1039/D0GC02301A.

39. European Network of Transmission System Operators for Electricity. *Ten-Year Network Development Plan - Commodity Prices*. (2024) https://2024-data.entsos-tyndp-scenarios.eu/files/scenarios-inputs/Prices.zip (last accessed: 12.5.2026).
