# Supplementary material for: Mapping Future Energy Journeys Through Modeling the Global Transport of Synthetic Energy Carriers
Source: Nat Commun. 2026 Nov 7;17:11500. doi: 10.1038/s41467-026-78710-7 (PMC13743743; doi:10.1038/s41467-026-78710-7)
Supplement: Supplementary file 2 [file 41467_2026_78710_MOESM2_ESM.pdf]

Description of Additional Supplementary Files

Supplementary Data 1: weighted average cost of capital (WACC) for all countries or territories.
