# Supplementary material for: Mapping Future Energy Journeys Through Modeling the Global Transport of Synthetic Energy Carriers
Source: Nat Commun. 2026 Nov 7;17:11500. doi: 10.1038/s41467-026-78710-7 (PMC13743743; doi:10.1038/s41467-026-78710-7)
Supplement: Supplementary file 3 [file 41467_2026_78710_MOESM4_ESM.pdf]

nature portfolio

Corresponding author(s): Uwe Langenmayr
uwe.langenmayr@kit.edu

Last updated by author(s): Aug 24, 2026

Reporting Summary

Nature Portfolio wishes to improve the reproducibility of the work that we publish. This form provides structure for consistency and transparency in reporting. For further information on Nature Portfolio policies, see our Editorial Policies and the Editorial Policy Checklist.

Statistics

For all statistical analyses, confirm that the following items are present in the figure legend, table legend, main text, or Methods section.

n/a	Confirmed	
☒	☐	The exact sample size (n) for each experimental group/condition, given as a discrete number and unit of measurement
☒	☐	A statement on whether measurements were taken from distinct samples or whether the same sample was measured repeatedly
☒	☐	The statistical test(s) used AND whether they are one- or two-sided<br>*Only common tests should be described solely by name; describe more complex techniques in the Methods section.*
☒	☐	A description of all covariates tested
☒	☐	A description of any assumptions or corrections, such as tests of normality and adjustment for multiple comparisons
☒	☐	A full description of the statistical parameters including central tendency (e.g. means) or other basic estimates (e.g. regression coefficient) AND variation (e.g. standard deviation) or associated estimates of uncertainty (e.g. confidence intervals)
☒	☐	For null hypothesis testing, the test statistic (e.g. F, t, r) with confidence intervals, effect sizes, degrees of freedom and P value noted<br>*Give P values as exact values whenever suitable.*
☒	☐	For Bayesian analysis, information on the choice of priors and Markov chain Monte Carlo settings
☒	☐	For hierarchical and complex designs, identification of the appropriate level for tests and full reporting of outcomes
☒	☐	Estimates of effect sizes (e.g. Cohen's d, Pearson's r), indicating how they were calculated

Our web collection on statistics for biologists contains articles on many of the points above.

Software and code

Policy information about availability of computer code

Data collection	No primary experimental data were collected. Publicly available spatial data were assembled and preprocessed using the custom HERMES software (Hydrogen Economy Routing Model for Cost-Efficient Supply), version 1.0.0, implemented in Python 3.11. Oil- and gas-pipeline data were obtained from the Global Energy Monitor, port data from the World Bank, geographical boundaries from Natural Earth, and water-stress data from the World Resources Institute Aqueduct database. Maritime routes and distances were generated using SeaRoute v1.3.1. Hydrogen production costs and potentials were derived from spatially resolved solar photovoltaic and onshore-wind potentials using atlite v0.2.10 and ERA5 reanalysis data, following the methodology described in the Supplementary Information. Transport and conversion parameters were compiled from the literature; some shipping-cost parameters were obtained from the spreadsheet calculation tool accompanying Staiß et al.

Data analysis

All computational analyses and visualizations were performed using the custom HERMES software (Hydrogen Economy Routing Model for Cost-Efficient Supply), version 1.0.0, implemented in Python 3.11. HERMES was used to preprocess spatial infrastructure data, generate transport networks, calculate shortest paths, identify cost-efficient multimodal transport and conversion routes, aggregate supply potentials, and produce the reported figures. The software environment included NumPy v1.26.4, pandas v2.2.x, SciPy v1.15.3, Joblib v1.3.2, NetworkX v3.2.1, PyTables v3.9.2, openpyxl v3.1.5, Shapely v2.0.3, GeoPandas v0.14.3, Fiona v1.9.6, PyProj v3.7.1, Rtree v1.4.0, GeoPy v2.4.1, geovoronoi v0.4.0, GeoJSON v3.1.0, vincenty v0.1.4, SeaRoute v1.3.1, Matplotlib v3.8.3, Seaborn v0.13.2, Plotly v6.7.0, CairoSVG v2.7.1, tqdm v4.66.2, psutil v5.9–7.x, PyYAML v6.x, gurobipy v12.0.3, and Gurobi Optimizer v12.0.3. OpenAI Codex and ChatGPT GPT-5.5 were used to support code development and language editing; all AI-assisted outputs were critically reviewed, validated, and revised by the authors. The complete dependency specification is provided with the archived source code. The main calculations were conducted on Ubuntu 20.04.6 LTS.

The raw input data are available on Zenodo at https://doi.org/10.5281/zenodo.22031725, while the HERMES v1.0.0 source code is archived on Zenodo at https://doi.org/10.5281/zenodo.22077166 and maintained on GitHub at https://github.com/ulicious/hermes.

For manuscripts utilizing custom algorithms or software that are central to the research but not yet described in published literature, software must be made available to editors and reviewers. We strongly encourage code deposition in a community repository (e.g. GitHub). See the Nature Portfolio guidelines for submitting code & software for further information.

Data

Policy information about availability of data

All manuscripts must include a data availability statement. This statement should provide the following information, where applicable:

- Accession codes, unique identifiers, or web links for publicly available datasets
- A description of any restrictions on data availability
- For clinical datasets or third party data, please ensure that the statement adheres to our policy

No primary experimental data were collected. The analysis used oil- and gas-pipeline data from the Global Energy Monitor, port data from the World Bank, geographical boundaries from Natural Earth, water-stress data from the World Resources Institute Aqueduct database, and ERA5 reanalysis data. Spatially resolved solar-photovoltaic and onshore-wind potential data were processed with atlite v0.2.10 to derive hydrogen-production potentials and costs. Transport and conversion parameters were compiled from the literature, and shipping-cost parameters were obtained from the spreadsheet calculation tool accompanying Staß et al. Maritime routes and distances were generated with SeaRoute v1.3.1. HERMES generated the processed spatial infrastructure datasets, transport-network representations, shortest-path results, cost-efficient multimodal transport and conversion routes, and aggregated supply-potential results used in the analysis and figures.

The data generated and used in this study have been deposited in Zenodo (https://doi.org/10.5281/zenodo.22031725). The data underlying all manuscript figures are provided in the accompanying Source Data file. Additional figures, notes, tables, methods, and data required for the analyses are provided in the Supplementary Information and Supplementary Data files. No private or restricted-access data were used in this study.

Research involving human participants, their data, or biological material

Policy information about studies with human participants or human data. See also policy information about sex, gender (identity/presentation), and sexual orientation and race, ethnicity and racism.

Reporting on sex and gender	n/a
Reporting on race, ethnicity, or other socially relevant groupings	n/a
Population characteristics	n/a
Recruitment	n/a
Ethics oversight	n/a

Note that full information on the approval of the study protocol must also be provided in the manuscript.

Field-specific reporting

Please select the one below that is the best fit for your research. If you are not sure, read the appropriate sections before making your selection.

☐ Life sciences ☐ Behavioural & social sciences ☒ Ecological, evolutionary & environmental sciences

For a reference copy of the document with all sections, see nature.com/documents/nr-reporting-summary-flat.pdf

Ecological, evolutionary & environmental sciences study design

All studies must disclose on these points even when the disclosure is negative.

Study description

This computational modeling study evaluates cost-efficient global supply routes for hydrogen and hydrogen-derived synthetic energy

Study description	carriers for the target year 2030. HERMES combines spatially resolved production costs and potentials with ports, shipping routes, oil and gas pipelines, road transport, and commodity-conversion options. The analysis covers 10,883 globally distributed potential export locations. Seven scenarios vary the eligible target commodities, pipeline retrofitting, waste-heat availability, commodity prices, and water-availability restrictions. Germany is the principal destination; China, India, Japan and the United States are examined as additional destinations.
Research sample	n/a
Sampling strategy	n/a
Data collection	n/a
Timing and spatial scale	n/a
Data exclusions	Pipeline segments were excluded if they were not classified as operating or under construction or lacked usable geographical information. Candidate export locations outside eligible land areas or without the required input data were excluded. In the water-restricted scenario, locations with a Baseline Water Stress indicator above 3 were excluded. No observations were excluded based on calculated transport costs or model outcomes.
Reproducibility	n/a
Randomization	n/a
Blinding	n/a

Did the study involve field work? ☐ Yes ☒ No

Reporting for specific materials, systems and methods

We require information from authors about some types of materials, experimental systems and methods used in many studies. Here, indicate whether each material, system or method listed is relevant to your study. If you are not sure if a list item applies to your research, read the appropriate section before selecting a response.

Materials & experimental systems

n/a	Involved in the study	
☒	☐	Antibodies
☒	☐	Eukaryotic cell lines
☒	☐	Palaeontology and archaeology
☒	☐	Animals and other organisms
☒	☐	Clinical data
☒	☐	Dual use research of concern
☒	☐	Plants

Methods

n/a	Involved in the study	
☒	☐	ChIP-seq
☒	☐	Flow cytometry
☒	☐	MRI-based neuroimaging

Plants

Seed stocks	n/a
Novel plant genotypes	n/a
Authentication	n/a
