# Supplementary material for: Mapping Future Energy Journeys Through Modeling the Global Transport of Synthetic Energy Carriers
Source: Nat Commun. 2026 Nov 7;17:11500. doi: 10.1038/s41467-026-78710-7 (PMC13743743; doi:10.1038/s41467-026-78710-7)
Supplement: Supplementary file 4 [file 41467_2026_78710_MOESM5_ESM.pdf]

nature portfolio

Peer Review File

Mapping Future Energy Journeys Through Modeling the Global Transport of Synthetic Energy Carriers

Corresponding Author: Dr Uwe Langenmayr

This file contains all reviewer reports in order by version, followed by all author rebuttals in order by version.

Version 0:

Reviewer comments:

Reviewer #1

(Remarks to the Author)
This manuscript presents a comprehensive, high-resolution global modeling framework for assessing the most cost-effective routes for transporting synthetic energy carriers (SECs), integrating spatially detailed data on renewable energy potential, infrastructure, and techno-economics of Power-to-X (PtX) supply chains. The study contributes a valuable open-source model and data platform that fills critical gaps in existing literature by providing granularity at the subnational level and incorporating multimodal transport and infrastructure retrofitting scenarios. It stands out for its methodological rigor, relevance to current energy transition debates, and potential utility for policymakers and infrastructure planners. However, despite these strengths, the paper is limited by a lack of full techno-economic validation for newer SEC technologies. Furthermore, the optimization is heuristic in nature, and no formal optimality guarantees are provided. Considering Nature Communications' high bar for methodological novelty, empirical robustness, and scientific generality, I recommend major revision. The work is promising, but it requires stronger engineering and modeling validations, clearer articulation of uncertainties, and expanded analysis of cross-regional impacts.

Specific Comments :
1. The authors use a heuristic mixed-integer optimization with a branch-based algorithm. However, there is no comparative validation against an exact solver on a simplified test case to assess solution accuracy or computational trade-offs.
2. The model assumes fixed capacities for transport infrastructure and no congestion or bottlenecks at ports or pipelines. Future-scale SEC deployment will likely induce such effects and should be at least discussed or modeled as constraints.
3. A cumulative energy demand breakdown across the supply chain would provide a more engineering-grounded comparison of the actual energy losses involved in SEC transformations and transports.
4. The model assumes any SEC (methanol, ammonia, etc.) is equally acceptable at the destination. In reality, end-user compatibility, re-conversion infrastructure, and chemical feedstock demand vary greatly by country and sector.
5. The 2030 snapshot does not account for time-dependent factors such as seasonal renewable availability, ramping of infrastructure, or load-following behavior. A dynamic time-series optimization (e.g., hourly resolution) could offer more realism.
6. The focus is entirely on cost optimization, with no quantification of GHG emissions, water use, or environmental trade-offs. Even indicative results would strengthen the policy relevance.
7. Liquefaction of hydrogen and SEC conversion efficiencies are stated but not integrated into a full energy balance that tracks yield loss over the supply chain. Boil-off gas from LH2 should also be modeled.
8. Where applicable, SEC transport cost estimates should be benchmarked against current fossil carrier logistics (e.g., LNG, methanol, ammonia trades) for realism.
9. Although the spatial resolution is higher and the number of export nodes is larger than prior models, the core modeling framework—minimizing transport and conversion cost—is conceptually incremental. There is limited novelty beyond scaling and dataset integration, and no clear theoretical or methodological innovation.
10. The study focuses heavily on Germany and a few importing countries, and while it claims global applicability, the model is not demonstrated across broader energy systems or regional policy contexts. Additionally, potential demand-side constraints, geopolitical factors, and institutional feasibility are omitted entirely.

(Remarks on code availability)

Reviewer #2

(Remarks to the Author)
The manuscript presents HERMES, a heuristic model designed to identify the least-cost transport routes for a range of hydrogen-based end-use commodities. The model notably includes the option of converting the commodity form to minimize transport costs and features a detailed spatial analysis with high granularity. The study draws general and insightful conclusions on trade-offs between commodity types and export locations. The results are well-aligned with existing literature, and from a spatial perspective, the work represents the most detailed analysis I have encountered in this domain. Overall, I find the study highly relevant and a valuable contribution to the hydrogen transport literature.
Before publication, however, I recommend that the following issues be addressed:
1. Clarification of Research Gaps:
In the introduction, the manuscript states: "To contribute to the existing literature and to close still existing research gaps." Please specify these research gaps more explicitly to position the study more clearly within the existing body of literature.
2. Ambiguity in Citation (p.4):
The phrase "In the main paper," is unclear. Please clarify which document is being referenced.
3. Invalid References (p.5 & p.13):
There are several invalid references in the manuscript. Please carefully check and correct all citations.
4. Figure Clarifications (Maps):
o Figure 1&3: Indicate the lowest and highest values of the colorbar for better interpretability.
5. Comparison of Commodities:
The manuscript compares the cost-effectiveness of different hydrogen carriers on the basis of transport costs. However, these carriers may serve different end-use applications (e.g., industrial feedstock, power generation, mobility), which affects their overall viability. Please explicitly state that your comparison refers to energetic use only, to avoid misleading conclusions.
6. Literature Context:
The sentence "These results align with recent research.20,21,24,27,36" could be expanded by also citing source 30, which arrives at similar conclusions and would strengthen the connection to related work.
7. Boil-off Losses in LH_2 Transport (p.17):
While I understand the global scope limits the inclusion of detailed local constraints such as port capacities, the exclusion of boil-off losses in LH_2 transport is critical. Boil-off can account for up to 10% of total supply costs, depending on distance and storage conditions. I strongly recommend that the model incorporate boil-off effects for LH_2 transport to more accurately reflect real-world economics.
8. Transparency and Assumptions:
The availability of the model and input data on GitHub is commendable. However, I recommend that a table summarizing the key techno-economic assumptions (including parameter units) be added to the Methods section. This would substantially improve the readability of the work. The current documentation in techno_economic_data_conversion.yaml is insufficient, as most parameters lack unit specification.
9. Production Cost Basis:
The methodology for calculating the production cost component of the total supply cost is unclear. Please elaborate on the data sources, assumptions, and calculation methods used.
10. Infrastructure Assumptions:
It is not clear whether the model assumes only existing infrastructure or if investment in new infrastructure (e.g., construction of new hydrogen pipelines) is also considered. Given the nascent stage of global hydrogen sector, I believe the possibility of new infrastructure development is highly relevant and should be discussed, if not modeled.

If these comments are adressed, I deem the paper ready for publication.

(Remarks on code availability)
I did review the code, but did not try to run it.
I noticed, that the documentation in techno_economic_data_conversion.yaml is insufficient, as most parameters lack unit specification.

Reviewer #3

(Remarks to the Author)
Langenmayr et al. introduce HERMES, a novel global modeling framework for optimizing the transport of synthetic energy carriers (SECs). The model incorporates geographically detailed infrastructure and seven hydrogen-based SECs to identify cost-effective trade routes between over 10,000 global export locations and major importing economies. The study is a valuable and timely contribution to the literature on hydrogen economy logistics and infrastructure planning.

The integration of high-resolution infrastructure data with multimodal transport and conversion pathways is a key advancement over region-aggregated energy models. The publication of both the model and data enhances transparency and enables replication and further exploration. The scale and detailed resolution of the geographic modeling allow localized insights across continents.

However, there are still some open questions that need clarification.
1. Which fuels are included as SECs? The text introduces Methylcyclohexane (MCH) and Dibenzyltoluene (DBT) as SECs, but provides no substantive discussion or quantitative results on their use. These carriers are not clearly represented in the

figures or scenario breakdowns, and lack sufficient introduction for readers unfamiliar with their properties or technological readiness. If these SECs are included in the model, their roles and selection contexts should be clearly explained. Furthermore, Two scenarios are constructed with gaseous hydrogen fixed as the import commodity, but no compelling rationale is provided for this assumption. The authors should explain why gaseous hydrogen, rather than ammonia or methanol, was prioritized, especially given the widespread view that derivatives will dominate long-distance trade in the medium term. Please explain what is meant with “In contrast, hydrogen lacks a fossil counterpart in international trade”; as far as I know hydrogen can be liquefied and transported. internationally.

2. Lack of fossil fuel displacement estimates: The paper would benefit from estimating how much fossil fuel use could be displaced under each scenario; either within the model itself or as a supplementary analysis. These estimates are essential for situating the findings within broader decarbonization efforts and assessing the real climate impact of the modeled supply chains.

3. Import country selection needs clarification: The rationale for selecting Germany, Japan, China, India, and the United States as import destinations is unclear. While Germany and Japan are well-established energy importers, the United States is now a net LNG exporter, and China and India may develop domestic hydrogen capacity. Meanwhile, South Korea—a highly import-dependent industrial nation with strong hydrogen strategies—is omitted. The paper should clarify whether these countries are selected based on modeled demand, illustrative purposes, or strategic energy forecasts.

4. Short time horizon (2030) limits policy relevance: Most hydrogen infrastructure, including liquefaction, ammonia cracking, and LOHC deployment, will not reach commercial scale by 2030. A 2050 scenario would align better with international decarbonization timelines (e.g., IMO, EU Green Deal) and would allow the inclusion of longer-term trends such as technology learning and global coordination mechanisms.

5. No carbon pricing or emissions constraints: The exclusion of carbon pricing and emissions intensity differentiation is a critical gap. Even a single scenario with a moderate carbon price would greatly enhance the model's policy relevance and provide insights into how price signals shape optimal trade flows and SEC choices.

6. Boil-off gases and potentially other losses omitted: Despite the global scope, important technical details, such as boil-off losses in liquid hydrogen transport, could still be incorporated in the analysis, in the same way that the authors considered other energy losses. While detailed modeling is understandably out of scope, the authors could assume standard uniform boil-off factors from the literature to improve realism.

7. Figures lack commodity-specific transparency: Figures 3 and 4 would benefit from overlaying information about which SECs dominate at different cost levels and volumes. For example, the absence of conversion costs in the initial volumes under GER-H2-Retrofit appears to reflect direct gaseous hydrogen delivery, but this is not made explicit. Adding this layer would clarify interpretation and support policy translation.

8. Organization of results and readability for general audiences: Given the journal's generalist readership, the presentation of results should be clearer and better structured. For example, some headline results could be included in the abstract. Instead of highlighting main insights, the abstract currently rather highlights the technical accomplishments of the analysis. In addition, the results section should state the main results more succinct and clearly. The authors have done a good job already but it can still be improved to make sure the readers clearly grasp the most important take-home messages.

9. Include line numbers in next revision: For transparency and to facilitate peer review, the authors should include line numbers in future manuscript revisions.

(Remarks on code availability)
I glanced over the code and it looked fine to me but I acknowledge that my familiarity with Python is very limited.

Version 1:

Reviewer comments:

Reviewer #2

(Remarks to the Author)

(Remarks on code availability)

Reviewer #3

(Remarks to the Author)
Generally, the responses to my comments are satisfactory, while the text added to the manuscript is not always sufficient.
1. Satisfied.

2. The response is fine, but I cannot find the supplementary information on the technical RFNBO/H_2 potentials anywhere in the manuscript or the SI. Please indicate more clearly where this information can be found.
3. What report did you base your selection on? The report needs to be properly cited, and a bit more justification is needed for why you ultimately selected these regions. I am not saying that other regions should have been selected, but the current justification still seems insufficient.
4. Fine.
5. Fine.
6. Addressed.
7. Addressed.
8. Partially addressed; see comments 2 and 3.
9. Addressed.

(Remarks on code availability)

Open Access This Peer Review File is licensed under a Creative Commons Attribution 4.0 International License, which permits use, sharing, adaptation, distribution and reproduction in any medium or format, as long as you give appropriate credit to the original author(s) and the source, provide a link to the Creative Commons license, and indicate if changes were made.
In cases where reviewers are anonymous, credit should be given to 'Anonymous Referee' and the source.
The images or other third party material in this Peer Review File are included in the article's Creative Commons license, unless indicated otherwise in a credit line to the material. If material is not included in the article's Creative Commons license and your intended use is not permitted by statutory regulation or exceeds the permitted use, you will need to obtain permission directly from the copyright holder.
To view a copy of this license, visit https://creativecommons.org/licenses/by/4.0/

Response to the Reviewers

Dear reviewers,

We sincerely appreciate your recognition of the novelty of our research in addressing important questions and for allowing us to refine our work to meet the standards of Nature Communications. We have put considerable effort into addressing all your comments, and we hope our revision meets your expectations.

We also want to apologize for the delay in providing our revision. The review required significant adjustments to the code, the implementation of an optimization model to validate our heuristic, the recalculation of all scenarios, and the addition of new scenarios. In addition, our main author was in the process of completing his PhD, which consumed additional time and energy.

Before going into a detailed response, we want to mention the main adjustments to the manuscript, code, and supplementary information:

1. We have implemented a mixed-integer problem to assess the optimality of our found solutions and to compare the computational efficiency between the developed heuristic and the mixed-integer problem
2. We included boil-off losses and self-consumption of transported commodities as marine fuels. These were included in a novel efficiency assessment of the full supply chain
3. We included commodity prices in one scenario to compare the different commodities not just based on an energy basis, but to consider their economic value
4. We restructured our Results section in the main manuscript to increase readability and to consider new results. We have further improved readability by sharpening the research gap, improving references, and revising the manuscript and supplementary information.
5. We have checked the code and made sure that sufficient information, such as units, is available.
6. We have updated figures to make them more accessible

Of course, we have addressed all other reviewer comments as well, and you will find the detailed responses to your review below.

Editor Remarks

Your remark: Abbreviations should be defined at their first use, instead of providing a table
Our answer: We have deleted the table, and abbreviations are introduced when first used.

Your remark: There should be no punctuation in the title
Our answer: We have changed the title to: Mapping Future Energy Journeys Through Modeling the Global Transport of Synthetic Energy Carriers

Your remark: Section order is: Title, Abstract, Introduction, Results, Discussion, Methods, Data Availability, Code Availability, References, End Notes, Figure Legends, Tables
Our answer: We have adjusted the order of the Sections to fit the requirements of Nature Communications.

Your remark: Section results should be divided by subheadings less than 60 characters (incl spaces) that do not contain punctuation

Our answer: We have adjusted all subheadings in the result sections to comply with your formatting style

Your remark: Excel file containing the data of plots → name: “Source Data”, and include a brief description in your cover letter. The “Data Availability” section should also include the statement “Source Data are provided with this paper.”
Our answer: We have created the Source Data file, which contains all the data of the plots of the main manuscript. We have further added the required phrase to the data availability section in the main manuscript and added a brief description in the cover letter.

Your remark: Include line numbers in next revision: For transparency and to facilitate peer review, the authors should include line numbers in future manuscript revisions.
Our answer: We have added line numbers to the manuscript

Reviewer 1

Your remark

The authors use a heuristic mixed-integer optimization with a branch-based algorithm. However, there is no comparative validation against an exact solver on a simplified test case to assess solution accuracy or computational trade-offs.

Our answer

Thank you very much for this important remark. We have conducted comprehensive changes and analysis of our model to investigate the solution accuracy and computational trade-offs.

First, we have implemented additional data processing to generate the data required for a mixed-integer model. This data generation uses the exact same configuration as the heuristic to ensure an exact comparison.

Second, we have conducted two case studies using the *All-Retrofit* scenario with different geographical scopes to assess the ability to scale the mixed-integer problem. The first case considers only Belgium, the Netherlands, and Luxembourg (~500,000 binaries), while the second case considers the transport of commodities from Morocco and Algeria to Spain (~1.75 Mio. binaries). In combination with these two case studies, we implemented three settings. The first one only sets up the mixed-integer problem without any additional input. The second approach uses the heuristic's calculated results as a warm-start solution. The third approach uses, in addition to the solution as a warm start, the solution's total supply costs to filter out impossible edges based on cost, reducing the size of the mixed-integer problem. The full approach and results are shown in *Supplementary Note 3*.

In short, the mixed-integer problem resulted in the following insights:

- Both case studies did not calculate any feasible solution if no warm start was provided
- If the warm start was provided, it was always accepted as a feasible solution. However, the gurobi solver did not achieve a better solution than this warm start in all cases. Here, we set a 60-minute time limit. We even ran a single optimization overnight without any improvement, just for testing.
- If a warm start was provided and the total supply costs were used to filter the edges, we had the following behavior:

- If sufficient edges were removed, the solver terminated the calculations and achieved optimality within the default MIP gap. In all these cases, no better solution was found. In the Netherlands-Belgium-Luxembourg case, this often required only a few seconds. In the Morocco-Algeria-Spain case, the required calculation time ranged from a few seconds to a few minutes, depending on the number of removed edges.
- If insufficient edges were removed, the solver could not prove optimality within the time limit of 60 minutes. In all these cases, no better solution than the warm start was found either.

Based on this result and the behavior of the solver, we are confident that our approach achieves optimality while outperforming the Gurobi solver regarding required computational time resources.

Your remark

The model assumes fixed capacities for transport infrastructure and no congestion or bottlenecks at ports or pipelines. Future-scale SEC deployment will likely induce such effects and should be at least discussed or modeled as constraints.

Our answer

Thank you for this valuable remark. We agree that infrastructure capacities and bottlenecks may become highly relevant under large-scale SEC deployment scenarios.

We added this aspect to the discussion and limitations section of the manuscript. At the current stage, incorporating realistic infrastructure capacities is challenging for two main reasons. First, globally consistent and sufficiently detailed capacity data for ports, pipelines, and related infrastructure are unavailable.

The adjustment can be found in the manuscript, starting in line 298: *Our global scope further limits the possibility of including more detailed transport and conversion data, such as capacities of ports, pipelines, and ships, loading and unloading processes, waste heat availability in ports, canal and port fees, or economies of scale for conversion plants*

Second, our approach is primarily designed as a what-if assessment of potential supply routes rather than a detailed representation of existing infrastructure utilization. By assuming unconstrained capacities, the model identifies the theoretical potential of transport pathways under the assumption that sufficient infrastructure expansion is realized. In addition, the applied transport costs are based on marginal transport costs rather than investment-driven expansion costs. Consequently, the underlying cost assumptions are consistent with the assumption of scalable infrastructure capacities.

Your remark

A cumulative energy demand breakdown across the supply chain would provide a more engineering-grounded comparison of the actual energy losses involved in SEC transformations and transports.

Our answer

Thank you for this important suggestion. To provide a more engineering-oriented assessment of the transport pathways, we added an evaluation of the supply-chain efficiency.

Based on this analysis, we included two additional figures in the supplementary information (*Supplementary Note 1*): (i) a visualization of the efficiency of the most cost-efficient route for each origin location and (ii) the average efficiency of the most frequently used transport routes.

We would like to note that the reported efficiencies describe the losses associated with transporting and converting the selected commodity along the supply chain. They do not represent a full cumulative primary energy demand assessment, including all upstream energy inputs.

Your remark

The model assumes any SEC (methanol, ammonia, etc.) is equally acceptable at the destination. In reality, end-user compatibility, re-conversion infrastructure, and chemical feedstock demand vary greatly by country and sector.

Our Answer

We agree that end-use compatibility, reconversion requirements, and commodity-specific applications differ substantially across sectors and countries. To address this aspect, we extended the model by introducing commodity prices in an additional scenario (*GER-All-Retrofit-Prices*). This allows the optimization to consider not only total supply costs, but also the economic value of individual commodities. The methodological implementation of this extension is described in the Methodology/General Problem section, with corresponding results presented in the final paragraph of the Results section. The calculation of commodity values is provided in *Supplementary Method 5*.

At the same time, the primary focus of this work remains the assessment of how different energy carriers are selected under varying production and infrastructure conditions. This includes scenarios in which conversions serve only as intermediate steps to supply hydrogen to the importing region (our -H2 scenarios), as well as scenarios in which all commodities are assumed to be acceptable at the destination (our -ALL scenarios). The latter reflects cases where multiple commodities are expected to play a role, for example, in regions such as Germany, where future demand may encompass renewable hydrogen, ammonia, methanol, and FT fuels, particularly given ongoing uncertainty regarding final energy carriers in sectors like power generation and transport. Despite potential non-energetic (material) uses of these commodities, we compare them based on their heating value to maintain a consistent metric.

Your remark

The 2030 snapshot does not account for time-dependent factors such as seasonal renewable availability, ramping of infrastructure, or load-following behavior. A dynamic time-series optimization (e.g., hourly resolution) could offer more realism.

Our answer

Thank you for this important remark. We fully agree that dynamic time-series optimization with high temporal resolution would provide a more detailed representation of renewable availability, infrastructure utilization, and operational flexibility.

We would like to clarify that temporal variability is considered in the calculation of hydrogen production and electricity generation costs. These costs are derived using an optimization framework for stand-alone PtX systems with hourly renewable generation profiles (see *Supplementary Method 2*).

However, the transport optimization itself is based on a single representative time snapshot. This modeling choice was made to ensure computational tractability while maintaining the global scope and high spatial and infrastructural resolution of the transport network.

In general, there is a trade-off between temporal detail and spatial/infrastructural detail in large-scale hydrogen system models. While integrated energy system models can capture dynamic behavior at high temporal resolution, they often require substantial simplifications in spatial resolution and in the

representation of transport infrastructure. In contrast, the strength of our approach lies in the detailed representation of global transport routes and infrastructure configurations.

To clarify these modeling choices, we expanded the limitations discussion accordingly and added a discussion on potential soft-coupling approaches between our framework and integrated energy system models.

The adjustment can be found in the manuscript, starting in line 301: *Compared with hydrogen supply models using dynamic time-series optimization, our approach cannot assess infrastructure operation and, therefore, cannot answer questions about temporal aspects such as loading and unloading, storage, or infrastructure occupancy. This limitation is necessary to handle the high spatial resolution of production and infrastructure data.*

Your remark

The focus is entirely on cost optimization, with no quantification of GHG emissions, water use, or environmental trade-offs. Even indicative results would strengthen the policy relevance.

Our answer

We agree that cost is not the only relevant dimension for assessing future hydrogen supply chains, particularly in the context of low-carbon Power-to-X systems.

To partially address this aspect, we extended the analysis by incorporating water availability constraints in an additional scenario ("All-Retrofit-Water"). Specifically, locations with high water stress and locations more than 50 km from the nearest coastline were excluded from the assessment. The corresponding methodology and results are presented in the supplementary information.

At the same time, comprehensive quantification of GHG emissions and broader environmental assessment were beyond the scope of the present work, as such an assessment would require extensive life-cycle and location-specific data across all considered commodities and transport pathways.

We explicitly address this limitation in the manuscript and highlight environmental assessment as an important direction for future research.

The adjustment can be found in the manuscript, starting in line 296: *Greenhouse gases are not considered, but would enrich the analysis and help derive more accurate carbon footprints, which could further support the analysis of carbon pricing mechanisms.*

Your remark

Liquefaction of hydrogen and SEC conversion efficiencies are stated but not integrated into a full energy balance that tracks yield loss over the supply chain. Boil-off gas from LH2 should also be modeled.

Our answer

Thank you for this important remark. We have extended the model to incorporate boil-off losses during transport.

In addition, we introduced the possibility of using transported commodities for ship propulsion to reflect the potential future use of hydrogen, methanol, and ammonia as marine fuels. These effects are now included in the supply chain's efficiency assessment.

The methodological implementation and the corresponding assumptions are described in *Supplementary Method 4*, while the effects on transport efficiency and the supply chain's overall efficiency are discussed in *Supplementary Note 1*.

Your remark

Where applicable, SEC transport cost estimates should be benchmarked against current fossil carrier logistics (e.g., LNG, methanol, ammonia trades) for realism.

Our answer

Thank you for this valuable suggestion. The techno-economic assumptions used in our transport model are largely derived from existing fossil carrier logistics, including established transport systems for LNG, methanol, and ammonia. Therefore, many of the applied transport cost assumptions are already implicitly benchmarked against current industrial practice.

To improve transparency, we clarified this aspect in the supplementary information and provided the detailed assumptions, calculations, and transport cost parameters in *Supplementary Method 4*.

Your remark

Although the spatial resolution is higher and the number of export nodes is larger than prior models, the core modeling framework—minimizing transport and conversion cost—is conceptually incremental. There is limited novelty beyond scaling and dataset integration, and no clear theoretical or methodological innovation.

Our answer

We agree that the underlying optimization objective, minimization of transport and conversion costs, is conceptually related to existing network flow approaches. However, we believe that the methodological contribution of this work lies in enabling the solution of a highly resolved global transport network problem that exceeds the practical capabilities of conventional exact optimization approaches.

To support this point, we implemented a mixed-integer benchmark formulation for reduced problem sizes and demonstrated that exact solution approaches become computationally prohibitive when scaling toward the full spatial and infrastructural resolution considered in this work (see *Supplementary Note 3*). In contrast, the proposed heuristic framework enables the solution of the large-scale problem within practical computational times.

We therefore position our contribution primarily as a methodological advancement in large-scale hydrogen transport network modeling, particularly in terms of computational tractability, spatial detail, and infrastructure representation. We clarified this contribution more explicitly in the revised manuscript.

The description can be found in the manuscript, starting in line 72: *Our methodological contribution represents an advancement in large-scale hydrogen transport network modeling, particularly in terms of computational tractability, spatial resolution, and infrastructure representation.*

Your remark

The study focuses heavily on Germany and a few importing countries, and while it claims global applicability, the model is not demonstrated across broader energy systems or regional policy contexts. Additionally, potential demand-side constraints, geopolitical factors, and institutional feasibility are omitted entirely.

Our answer

Thank you for this important remark. We agree that the present study focuses on a limited set of importing countries (Germany, the US, China, Japan, and India) and does not explicitly assess demand-side constraints, geopolitical factors, or institutional feasibility.

While the framework itself is globally applicable, we selected a limited number of import countries for the manuscript in order to keep the analysis focused and interpretable. The selected countries are based on major potential SEC demand regions identified in the literature. Since the required global input data are already implemented, the analysis can be extended to other destination countries by specifying the target country in the configuration file.

The adjustment can be found in the manuscript, starting in Table 1 in the Tables section (scenario description): *Moreover, the underlying database covers all countries, enabling users to run these calculations without additional data collection by specifying the desired import country in the model configuration.*

We also agree that demand-side dynamics, geopolitical considerations, and institutional feasibility are highly relevant for future hydrogen trade. However, these aspects require market or energy system models that endogenously determine demand, export availability, and regional constraints under socio-political and economic conditions. Our model is therefore best understood as a transport-focused framework that identifies potential cost-efficient supply routes and infrastructure configurations.

We clarified this scope in the revised discussion and added the omitted aspects as important limitations and directions for future model coupling.

The adjustment can be found in the manuscript, starting in line 304: *Nevertheless, we see significant potential to combine the two modeling approaches. For example, our approach could be used to analyze distinct locations and provide data, such as supply curves, for energy system models with a regional focus, models analyzing the crowding-out effects of conventional energy carriers, or models identifying congestion and bottlenecks in transport infrastructure.*

Reviewer 2

Your remark

Clarification of Research Gaps: In the introduction, the manuscript states: "To contribute to the existing literature and to close still existing research gaps." Please specify these research gaps more explicitly to position the study more clearly within the existing body of literature.

Our answer

Thank you for this helpful remark. We revised the introduction to more explicitly define the research gaps this study addresses. In particular, we now highlight the limited spatial resolution, simplified infrastructure representation, and restricted consideration of multiple SEC transport pathways in previous work, thereby clarifying the specific contribution and positioning of our transport-focused approach within the existing literature.

The adjustment can be found in the manuscript, starting in line 57: *Based on the literature, we identify a research gap: existing studies on global PtX commodity trade and SEC transport are limited by*

aggregated spatial representations, narrow case-study perspectives, or restricted consideration of transport and conversion options. Most approaches neglect detailed intra-national renewable energy potentials, local infrastructure availability, and geographically explicit multimodal transport networks, while studies addressing transport optimization often rely on abstract regions without real-world spatial context. Consequently, there remains a lack of an integrated, high-resolution modeling framework that combines detailed infrastructure representation, multiple SEC pathways, and realistic global transport dynamics to comprehensively assess future large-scale SEC trade.

Your remark

Ambiguity in Citation (p.4):
The phrase "In the main paper," is unclear. Please clarify which document is being referenced.

Our answer

Thank you for pointing out this ambiguity. We revised the wording to clearly specify the referenced document and improve readability.

Your remark

Invalid References (p.5 & p.13): There are several invalid references in the manuscript. Please carefully check and correct all citations.

Our answer

Thank you for mentioning this issue. We have adjusted the document accordingly and checked for all references to ensure completeness.

Your remark

Figure Clarifications (Maps):
o Figure 1&3: Indicate the lowest and highest values of the colorbar for better interpretability.

Our answer

We have added the lowest and highest values to all colorbars.

Your remark

Comparison of Commodities: The manuscript compares the cost-effectiveness of different hydrogen carriers on the basis of transport costs. However, these carriers may serve different end-use applications (e.g., industrial feedstock, power generation, mobility), which affects their overall viability. Please explicitly state that your comparison refers to energetic use only, to avoid misleading conclusions.

Our answer

Thank you for this important remark. We agree that the suitability of individual SECs strongly depends on their specific end-use applications and that an energy-based comparison alone may not fully capture their role as chemical feedstocks or industrial intermediates.

We therefore clarified in the manuscript that while our comparison primarily refers to energetic use and hydrogen supply applications, we acknowledge the upcoming material use of SECs in an additional scenario (*GER-All-Retrofit-Prices*) by introducing commodity prices to account for the economic value of the transported commodity. The methodological implementation and corresponding results are now included in the revised method section (manuscript line 357) and the *Supplementary Method 5*.

To address your remark in the manuscript, we further adjusted the scenario description as follows (in Table 1 in Tables section): *As the model does not cover the end-use application of the SECs at the destination, supply costs are calculated on an energy content basis to ensure comparability across pathways. To acknowledge potential non-energetic, material uses of these commodities, commodity prices are included in the All-Retrofit-Prices scenario to compare their value at the destination.*

Your remark

Literature Context: The sentence "These results align with recent research.20,21,24,27,36" could be expanded by also citing source 30, which arrives at similar conclusions and would strengthen the connection to related work.

Our answer

Thank you for this attentive remark. We have adjusted the references accordingly.

Your remark

Boil-off Losses in LH_2 Transport (p.17): While I understand the global scope limits the inclusion of detailed local constraints such as port capacities, the exclusion of boil-off losses in LH_2 transport is critical. Boil-off can account for up to 10% of total supply costs, depending on distance and storage conditions. I strongly recommend that the model incorporate boil-off effects for LH_2 transport to more accurately reflect real-world economics.

Our answer

Thank you for this important remark. We agree that boil-off losses are a relevant factor for realistic LH_2 transport assessments and should be considered in the economic evaluation of long-distance transport routes.

To address this point, we extended the model by incorporating boil-off losses for liquid hydrogen transport. In addition, we included the possibility of consuming transported commodities as marine fuel in order to reflect the potential future use of hydrogen, methanol, and ammonia in maritime transport. Both effects are now incorporated into the supply chain efficiency assessment.

The corresponding methodological adjustments, assumptions, and calculations were added to *Supplementary Method 4,* while the updated MIP formulation is described in the Methods section of the main manuscript (below line 326). The resulting impact of boil-off losses and marine fuel consumption on route efficiencies is presented in *Supplementary Note 1*.

Your remark

Transparency and Assumptions: The availability of the model and input data on GitHub is commendable. However, I recommend that a table summarizing the key techno-economic assumptions (including parameter units) be added to the Methods section. This would substantially improve the readability of the work. The current documentation in techno_economic_data_conversion.yaml is insufficient, as most parameters lack unit specification.

Our answer

We have added all assumptions and parameters to the supplementary information (*Supplementary Method 1 to 5*). We have further improved the code by defining the units in the configuration files (yaml files) and the documentation as well.

Your remark

Production Cost Basis: The methodology for calculating the production cost component of the total supply cost is unclear. Please elaborate on the data sources, assumptions, and calculation methods used.

Our answer

We have revised the corresponding section to provide a clearer description of how the hydrogen production cost component is calculated.

In addition, in a revised description provided in *Supplementary Method 2*, we now describe the underlying data sources, key assumptions, and calculation procedure used to derive global hydrogen production costs, electricity generation costs, and hydrogen production potentials.

Your remark

Infrastructure Assumptions: It is not clear whether the model assumes only existing infrastructure or if investment in new infrastructure (e.g., construction of new hydrogen pipelines) is also considered. Given the nascent stage of global hydrogen sector, I believe the possibility of new infrastructure development is highly relevant and should be discussed, if not modeled.

Our answer

Thank you for this important remark. We agree that the potential expansion of hydrogen infrastructure is highly relevant for future global hydrogen trade.

In the current model setup, new hydrogen pipeline segments are considered to a limited extent by allowing the repurposing of existing natural gas pipelines and their extension over short distances, which can be adjusted by the user. We selected a limit of 100 km for additional pipeline segments as mentioned in section *Different infrastructure settings create carrier niches*, as longer distances would require more detailed geographical and infrastructural planning considerations.

We improved the description of these assumptions in *Supplementary Method 4* and expanded the discussion of this limitation in the manuscript. Furthermore, we outline how future work could incorporate more comprehensive infrastructure expansion approaches.

The adjustment can be found in the manuscript, starting in line 308: *Road transport routes and new pipeline segments are calculated using as-the-crow-flies distances adjusted by a detour factor, since more detailed information like geographical or street map data would substantially increase computational complexity. However, this additional effort would enable the consideration of larger infrastructure investments.*

Your remark

I noticed, that the documentation in techno_economic_data_conversion.yaml is insufficient, as most parameters lack unit specification.

Our answer

Thank you for pointing out this inaccuracy. We have adjusted the code accordingly.

Reviewer 3

Your remark

Which fuels are included as SECs? The text introduces Methylcyclohexane (MCH) and Dibenzyltoluene (DBT) as SECs, but provides no substantive discussion or quantitative results on their use. These carriers are not clearly represented in the figures or scenario breakdowns, and lack sufficient introduction for readers unfamiliar with their properties or technological readiness. If these SECs are included in the model, their roles and selection contexts should be clearly explained.

Our answer

Thank you for this important remark. LOHC-based transport pathways are currently underrepresented in the presented results because they are generally not identified as the most cost-efficient option under the assumptions applied in the main scenarios; however, they are considered in the heuristic.

A key reason is the heat demand associated with the dehydrogenation process. While this heat can either be externally supplied or partially generated through autothermal processes, we do not assume universally available low-cost heat sources in the main scenarios, as industrial waste heat is typically already utilized elsewhere. Under these assumptions, the dehydrogenation efficiency decreases substantially, which increases the resulting hydrogen supply costs from LOHC pathways.

To better illustrate the role and potential advantages of LOHCs, we reintroduced a dedicated scenario in the main manuscript, assuming suitable heat sources are available at relevant infrastructure locations. Under these conditions, LOHCs become competitive, particularly for short-distance maritime transport. We further expanded the discussion to better explain the technological characteristics, advantages, and limitations of LOHC-based transport systems.

This new result section starts in line 208 of the main manuscript using the following heading: *LOHC viability driven by heat available for dehydrogenation*

Your remark

Furthermore, Two scenarios are constructed with gaseous hydrogen fixed as the import commodity, but no compelling rationale is provided for this assumption. The authors should explain why gaseous hydrogen, rather than ammonia or methanol, was prioritized, especially given the widespread view that derivatives will dominate long-distance trade in the medium term. Please explain what is meant with “In contrast, hydrogen lacks a fossil counterpart in international trade”; as far as I know hydrogen can be liquefied and transported. internationally.

Our answer

Thank you for this important remark. Although hydrogen derivatives such as ammonia or methanol are expected to play a major role in long-distance transport, gaseous hydrogen itself remains highly relevant for several future applications, including steel production, refining, dispatchable power supply, and specific industrial heat applications.

By defining gaseous hydrogen as the target commodity, the model evaluates the cost of supplying hydrogen independently of the intermediate transport carrier used along the route. This allows ammonia, methanol, and other SECs to be assessed as hydrogen transport vectors within a consistent comparison framework.

To further address application-specific differences between commodities, we additionally introduced commodity prices into the model (*GER-All-Retrofit-Prices* scenario (description in Table 1 in Tables section) and the calculation of commodity prices in *Supplementary Method 5*). This extension allows

the optimization to consider not only total supply costs but also the economic value of individual commodities. In addition, users can specify the target commodity at the destination, allowing commodity-specific assessments.

Regarding the statement "hydrogen lacks a fossil counterpart in international trade," we clarified the wording in the revised manuscript. The intended meaning was that, unlike ammonia or methanol, there is currently no globally established large-scale fossil-based hydrogen trade infrastructure comparable to existing fossil commodity markets, even though hydrogen can technically be liquefied and transported internationally.

We adjusted the statement as follows (line 37 in the main manuscript): *Unlike ammonia or methanol, there is currently no globally established large-scale fossil-based hydrogen trade infrastructure comparable to existing fossil commodity markets*.

Your remark

Lack of fossil fuel displacement estimates: The paper would benefit from estimating how much fossil fuel use could be displaced under each scenario; either within the model itself or as a supplementary analysis. These estimates are essential for situating the findings within broader decarbonization efforts and assessing the real climate impact of the modeled supply chains.

Our answer

We thank the reviewer for this important comment. We agree that fossil fuel displacement is highly relevant for assessing the broader decarbonization implications of RFNBO supply chains. However, we would like to clarify that our model is primarily designed as a supply-side assessment of techno-economic RFNBO potentials and supply costs. It does not endogenously model sectoral demand, market uptake, fossil fuel substitution, or policy-driven adoption pathways. Therefore, translating the modeled RFNBO supply potentials into direct fossil fuel displacement estimates would require additional assumptions on end-use allocation, conversion efficiencies, sector-specific substitution options, and the fossil counterfactual.

To better contextualize our results, we have added a supplementary discussion comparing the global technical RFNBO/H_2 potentials considered in the case study with current global fossil fuel use. The global H_2 potential included in the case study amounts to approximately 519,000 TWh per year. This exceeds current global fossil fuel demand of roughly 140,000 TWh per year by a factor of 3.7. This comparison indicates that, from a purely technical resource perspective, the modeled RFNBO potentials are sufficiently large to contribute substantially to fossil fuel substitution.

At the same time, actual fossil fuel displacement will depend less on techno-economic potentials alone and more on the socio-economic and political framework conditions under which RFNBOs are deployed. Relevant factors include the ambition and design of decarbonization targets, whether net-zero pathways are pursued, the role and cost of CCS/CCU, sector-specific mandates and carbon pricing, infrastructure availability, and the willingness or ability of industry and transport sectors to pay higher energy prices or pass these costs on to consumers under conditions of international competition. We therefore interpret our results as an assessment of the technical and economic supply potential of RFNBOs, rather than as a direct estimate of realized fossil fuel displacement. This limitation and its implications for interpreting the climate impact of the modeled supply chains have now been clarified in the revised manuscript.

We adjusted the manuscript as follows (line 304 in the main manuscript): *Nevertheless, we see significant potential to combine the two modeling approaches. For example, our approach could be used to analyze distinct locations and provide data, such as supply curves, for energy system models*

with a regional focus, models analyzing the crowding-out effects of conventional energy carriers, or models identifying congestion and bottlenecks in transport infrastructure.

Your remark

Import country selection needs clarification: The rationale for selecting Germany, Japan, China, India, and the United States as import destinations is unclear. While Germany and Japan are well-established energy importers, the United States is now a net LNG exporter, and China and India may develop domestic hydrogen capacity. Meanwhile, South Korea—a highly import-dependent industrial nation with strong hydrogen strategies—is omitted. The paper should clarify whether these countries are selected based on modeled demand, illustrative purposes, or strategic energy forecasts.

Our answer

Thank you for pointing out this aspect. We have chosen these countries based on an report that states they may be major importers of renewable commodities. Our model does not assess a country's ability to self-supply its own demand. How global commodity trade will evolve is outside the scope of this model, as we focus only on potential routes for future hydrogen trade based on costs and quantities. However, since import (select country as destination) and export (select country not as destination) are modeled, both cases can be assessed, and the resulting data can be used to model a potential future hydrogen and hydrogen derivative market.

To address your remark, we have added references to the choice of the countries considered. We want to state that users of the fully accessible model can easily change the destination by specifying the import region in the configuration. Additional data collection is not required, making the code already applicable to all countries.

We adjusted the manuscript as follows (In Table 1 in Tables section): *Moreover, the underlying database covers all countries, enabling users to run these calculations without additional data collection by specifying the desired import country in the model configuration.*

Your remark

Short time horizon (2030) limits policy relevance: Most hydrogen infrastructure, including liquefaction, ammonia cracking, and LOHC deployment, will not reach commercial scale by 2030. A 2050 scenario would align better with international decarbonization timelines (e.g., IMO, EU Green Deal) and would allow the inclusion of longer-term trends such as technology learning and global coordination mechanisms.

Our answer

Thank you for this important remark. We agree that long-term scenarios, such as 2050, are highly relevant for assessing the future evolution of global hydrogen infrastructure and trade systems.

However, the objective of this study is not to predict the fully developed future hydrogen economy, but rather to assess near- to medium-term transport potentials based on current infrastructure availability and plausible retrofit assumptions. Our approach, therefore, follows a what-if perspective that aims to identify promising transport pathways and infrastructure requirements under early market conditions.

We clarified this objective more explicitly in the revised manuscript and added a discussion on how the framework could be extended toward longer-term scenarios in future work.

We adjusted the manuscript as follows (line 301 in the main manuscript): *Compared with hydrogen supply models using dynamic time-series optimization, our approach cannot assess infrastructure operation and, therefore, cannot answer questions about temporal aspects such as loading and unloading, storage, or infrastructure occupancy. This limitation is necessary to handle the high spatial resolution of production and infrastructure data.*

Your remark

No carbon pricing or emissions constraints: The exclusion of carbon pricing and emissions intensity differentiation is a critical gap. Even a single scenario with a moderate carbon price would greatly enhance the model's policy relevance and provide insights into how price signals shape optimal trade flows and SEC choices.

Our answer

Thank you for this important remark. We agree that carbon pricing schemes and emission constraints are likely to play a major role in shaping future hydrogen markets and transport pathways.

In the present work, however, all considered energy carriers are assumed to fulfill the Renewable Fuels of Non-Biological Origin (RFNBO) criteria of the European Union, implying comparatively low carbon intensities across all pathways. Consequently, the analysis focuses on transport and conversion economics within a low-carbon supply framework rather than competition with fossil-based alternatives.

We nevertheless acknowledge that carbon pricing mechanisms and differentiated emission intensities could significantly influence future trade flows and technology choices. We therefore added this aspect to the limitations discussion and highlighted it as an important avenue for future research.

We adjusted the manuscript as follows (line 296 in the main manuscript): *Greenhouse gases are not considered, but would enrich the analysis and help derive more accurate carbon footprints, which could further support the analysis of carbon pricing mechanisms.*

Your remark

Boil-off gases and potentially other losses omitted: Despite the global scope, important technical details, such as boil-off losses in liquid hydrogen transport, could still be incorporated in the analysis, in the same way that the authors considered other energy losses. While detailed modeling is understandably out of scope, the authors could assume standard uniform boil-off factors from the literature to improve realism.

Our answer

Thank you for this important remark. We agree that boil-off losses and other transport-related losses are important for improving the realism of LH_2 transport pathways.

To address this point, we extended the model by incorporating boil-off losses for liquid hydrogen transport. In addition, we included the possibility of consuming transported commodities as marine fuels, reflecting the potential future use of hydrogen, methanol, and ammonia in maritime transport. These effects are now included in the supply chain efficiency assessment (*Supplementary Note 1*).

The corresponding assumptions and calculations for boil-off losses and commodity consumption during transport are described in *Supplementary Method 4.* Furthermore, Equation 4 in the manuscript was revised to include transport efficiency effects resulting from boil-off and fuel consumption.

Your remark

Figures lack commodity-specific transparency: Figures 3 and 4 would benefit from overlaying information about which SECs dominate at different cost levels and volumes. For example, the absence of conversion costs in the initial volumes under GER-H2-Retrofit appears to reflect direct gaseous hydrogen delivery, but this is not made explicit. Adding this layer would clarify interpretation and support policy translation.

Our answer

Thank you for this helpful remark. We agree that making the commodity composition of the supply curves more explicit improves the interpretability of Figures 4 and 5 (previously Figures 3 and 4).

To address this point, we added a commodity band to the figures, indicating which SEC is transported for the corresponding segments of the supply curve. This additional layer makes it possible to identify the sequence of transported commodities and to distinguish direct hydrogen delivery from routes involving intermediate conversion steps.

In addition, we further disaggregated conversion and transport costs to more clearly show the sequence and contribution of the individual supply chain steps. This improves the transparency of the figures and supports a more direct interpretation of the modeled transport pathways.

Your remark

Organization of results and readability for general audiences: Given the journal's generalist readership, the presentation of results should be clearer and better structured. For example, some headline results could be included in the abstract. Instead of highlighting main insights, the abstract currently rather highlights the technical accomplishments of the analysis. In addition, the results section should state the main results more succinct and clearly. The authors have done a good job already but it can still be improved to make sure the readers clearly grasp the most important take-home messages.

Our answer

We have restructured the Results section and revised the complete manuscript, all figure and table captions, and the supplementary information for better readability.

Your remark

Include line numbers in next revision: For transparency and to facilitate peer review, the authors should include line numbers in future manuscript revisions.

Our response

We have added line numbers to the manuscript.

We would like to express our sincere gratitude to the reviewers for their valuable feedback, constructive suggestions, and the considerable time devoted to evaluating our work. Their comments have significantly improved the manuscript. We have carefully addressed each point and incorporated the suggested changes wherever possible. We believe that the revised manuscript has been substantially strengthened as a result of this process, and we hope that our responses and revisions adequately address all concerns.

On behalf of all authors, sincerely,
Uwe Langenmayr

Response to the Reviewers

Dear reviewers,

We thank you for your careful assessment of our revised manuscript and for your constructive suggestions. We have addressed the remaining points raised in the review and believe these changes have further improved the manuscript's clarity. We have addressed all open comments of the Editor and Reviewer 3. Please find the detailed responses below.

Editor Remarks

Your remark: Furthermore, please revise the “branching procedure” (in section methods) to either normal text with formulas, or move them to the supplementary information as a part of supplementary methods.

Our answer: We decided to move the branching procedure to the Supplementary Information (Supplementary Method 1) as suggested (see page 16, line 381-382 in the manuscript):

More information on the branching procedure can be found in Supplementary Method 1.

Your remark: We request that you review and follow the formatting instructions provided in the Nature Communications guidelines

Our answer: We double-checked and re-edited the manuscript according to the formatting instructions. However, we have not used the term “in this work” or a similar sentence in the last sentence of the abstract since the last 3 sentences of our abstract already describe the main findings of our work. If this is not intended, please let us know.

Reviewer 3

Your remark:

The response is fine, but I cannot find the supplementary information on the technical RFNBO/H_2 potentials anywhere in the manuscript or the SI. Please indicate more clearly where this information can be found.

Our answer:

We have added a short description of the methodology, the final potentials, and a figure showing the distribution of hydrogen production potentials in Supplementary Method 3, with further illustration in Supplementary Fig. 24.

Your remark:

What report did you base your selection on? The report needs to be properly cited, and a bit more justification is needed for why you ultimately selected these regions. I am not saying that other regions should have been selected, but the current justification still seems insufficient.

Our answer:

The selection of import countries was based on the Hydrogen Council report *Global Hydrogen Flows: Hydrogen Trade as a Key Enabler for Efficient Decarbonization* (October 2022), which is cited both in the Introduction and in the legend of Table 1. The countries are identified in this report as major prospective hydrogen importers, see the figure from the report below. Moreover, these countries are widely discussed as importers in further hydrogen literature, e.g., Galimova et al. (2023): *Global trading of renewable electricity-based fuels and chemicals to enhance the energy transition across all sectors towards sustainability*, Renewable and Sustainable Energy Reviews, DOI: 10.1016/j.rser.2023.113420.

To clarify this rationale, we revised the manuscript and added an explicit reference to the Hydrogen Council report in the Introduction, page 3, line 65, together with additional text explaining that the selected countries represent key anticipated hydrogen importers according to the report's analysis:

We develop a heuristic based on a mixed-integer optimization model to calculate the most cost-efficient transport routes and transport costs from 10,883 globally distributed energy export locations to the import countries Germany, Japan, China, India, and the United States by 2030, representing major prospective importers of renewable hydrogen products according to the Hydrogen Council.[34]

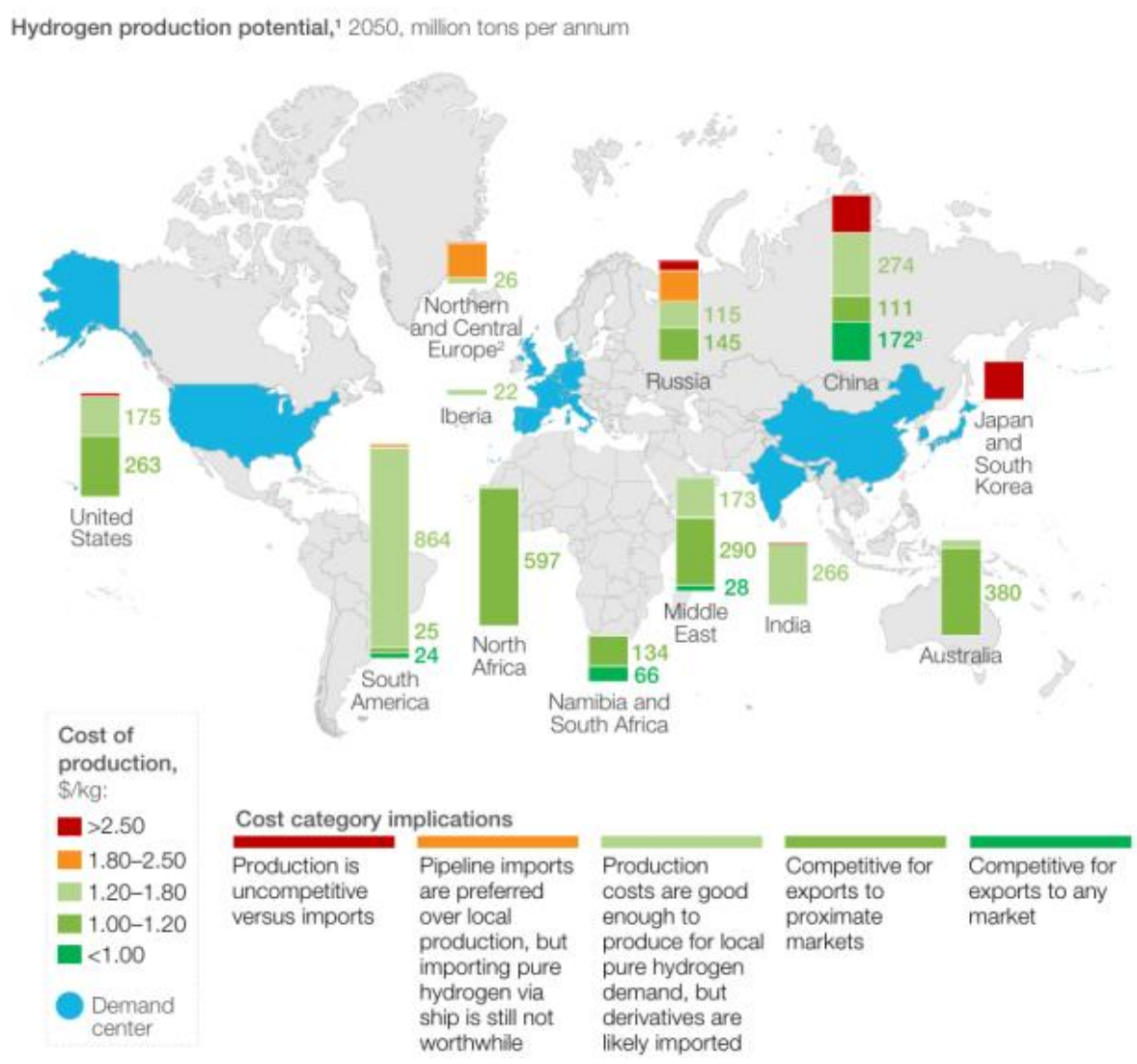

Your remark:

Partially addressed; see comments 2 and 3.

Our answer:

We have addressed your review comments 2 and 3 thoroughly.

We thank the reviewer for their helpful comments and suggestions. We have carefully addressed all points raised and incorporated the corresponding revisions into the manuscript. We hope that these changes satisfactorily address the reviewer's comments.

On behalf of all authors, sincerely,
Uwe Langenmayr
